# Silver-Free Gold-Catalyzed Heterocyclizations through Intermolecular H-Bonding Activation

**DOI:** 10.1021/acs.joc.2c02932

**Published:** 2023-01-27

**Authors:** Pilar Elías-Rodríguez, Esteban Matador, Manuel Benítez, Tomás Tejero, Elena Díez, Rosario Fernández, Pedro Merino, David Monge, José M. Lassaletta

**Affiliations:** † Facultad de Química. Departamento de Química Orgánica, Universidad de Sevilla and Centro de Innovación en Química Avanzada (ORFEO−CINQA), C/Prof. García González, 1, 41012 Sevilla, Spain; ¶ Instituto de Síntesis Química y Catálisis Homogénea (ISQCH), Universidad de Zaragoza-CSIC, 50009 Zaragoza, Spain; § Instituto de Biocomputación y Física de Sistemas Complejos (BIFI), Universidad de Zaragoza, 50009 Zaragoza, Spain; ‡ Instituto de Investigaciones Químicas (CSIC-US) and Centro de Innovación en Química Avanzada (ORFEO−CINQA), Avda. Américo Vespucio, 49, 41092 Sevilla, Spain

## Abstract

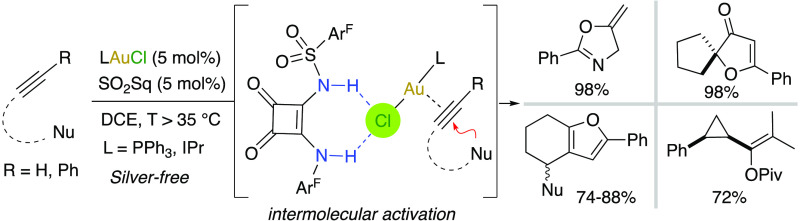

Modulable monosulfonyl squaramides have been shown to exert activation of gold(I) chloride complexes through H-bonding in an intermolecular way. Combinations of (PPh_3_)AuCl or IPrAuCl complexes and an optimal sulfonyl squaramide cocatalyst bearing two 3,5-bis(trifluoromethyl)phenyl groups efficiently catalyzed diverse heterocyclizations and a cyclopropanation reaction, avoiding in all cases undesired side reactions. Computational studies indicate that the Au–Cl bond breaks by transligation to the triple bond in a ternary complex formed by the actual AuCl···HBD catalyst and the substrate.

## Introduction

**Figure 1:**
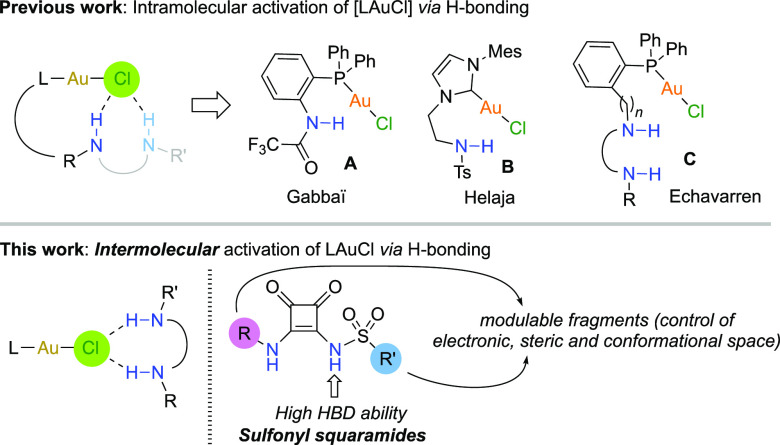
Silver-free activations of [LAuCl] via H-bonding in intra- and intermolecular fashion.

Gold catalysis has become a powerful tool for the synthesis of complex organic molecules.^1^ Gold(I) chloride complexes [LAuCl] are readily available and stable precatalysts that generally require activation to undergo useful catalytic activities. Typically, silver salts have played the role of chloride scavengers, although their light instability, hygroscopic nature, and the “silver effect” in catalysis often represent major drawbacks.^2^ Several approaches have been developed to overcome these practical issues,^3^ including the use of alternative alkali metal borates and copper salts,^4^ self-activation of gold(I) chloride complexes bearing specially designed ancillary ligands,^3^ and, more recently, H-bonding activation by certain solvents such as hexafluoroisopropanol (HFIP)^5^ as well as via halogen-bonding catalysis.^6^ Great efforts have been focused on the design and synthesis of [L–Au-Cl] complexes possessing multifunctional phosphine or NHC ligands bearing H-bond donor (HBD) groups (Figure 1, top), such as trifluoroacetamido (**A**),^7^*p*-tolyl-sulfonamido (**B**),^8^ and bidentate HBD groups (**C**).^9^ These reports constitute an excellent proof of concept of a synergistic Au(I)/ion-pairing strategy based on chloride abstraction from an electrophilic metal center by classical H-bond donors. In particular, Echavarren and co-workers have demonstrated that acidic HBD derivatives such as squaramides, with a proper linker length to the Au–Cl position, induce the higher activities.^9^ However, *intermolecular* activation of (PPh_3_)AuCl with untethered ureas/squaramides was unsuccessfully tested. We envisaged that the use of more acidic HBDs might overcome this limitation, providing a versatile approach which might benefit of the multiple combinations of ligands and HBD scaffolds (Figure 1, bottom). It is clear that the common use of more acidic thioureas/thiosquaramides is not an option in this case due to the high affinity of cationic gold(I) for the basic thiocarbonyl groups in these species, which would surely result in catalyst deactivation. On the other hand, an alternative to increase the acidity of NH-type bond donors is the introduction of electron-withdrawing scaffolds such as 3,5-bis(trifluoromethyl)phenyl, sulfinyl, and other moieties directly attached to NH groups.^10^ Although a few sulfinyl or sulfonyl squaramides have been reported to exhibit high potential in supramolecular^11^ and medicinal chemistry,^12^ their applications as catalysts or cocatalysts in chemical transformations remain underdeveloped.^13^ The presence of a sulfonyl group SO_2_R′ in monosulfonyl squaramides (SO_2_Sq) (Figure 1, bottom, right) ensures strong HBD abilities while the fragment R on the other NH group provides structural variability, thereby enabling the modulation of electronic, steric, and conformational properties, essential for catalysis. Additionally, the tetrahedral geometry at sulfur atom provides nonplanar conformations which might help prevent undesired self-aggregations and, in turn, enhance solubility in less polar organic solvents. In this article, we present the implementation of sulfonyl squaramides in the challenging intermolecular activation of gold chloride complexes for silver-free gold(I) catalyzed transformations.

## Results and Discussion

**Scheme 1:**
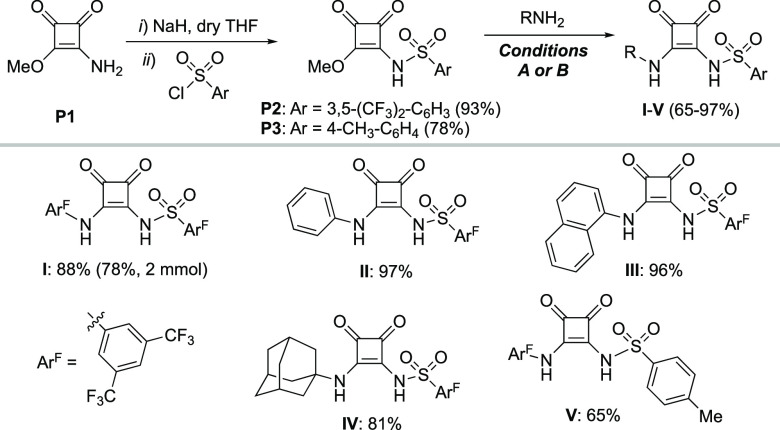
Synthesis of Sulfonyl Squaramides The synthesis of **I**–**V** was performed at 0.5 mmol scale.

Sulfonyl squaramides **I**–**V** were synthesized in 2 steps from known 3-amino-4-methoxycyclobut-3-ene-1,2-dione (**P1**)^14^ (Scheme 1). Installation of the sulfonamide was accomplished using sodium hydride and the sulfonyl chloride of choice, affording key intermediates **P2** and **P3** in 93% and 78% yield, respectively. The introduction of a second HBD moiety was achieved by displacement of the methoxy group in **P2**/**P3** with a primary amine. Thus, the corresponding sulfonyl squaramides **I**–**V** were obtained in good-to-excellent yields (65–97%). The reaction with aromatic amines required activation by Zn(OTf)_2_ in dry toluene at high temperatures (60–100 °C) (conditions A), while adamantyl amine reacted smoothy in CH_2_Cl_2_ at room temperature (conditions B). Additionally, the synthesis of **I** could be performed at 2 mmol scale in 78% yield.

It is well-known that squaramides form self-aggregates that often limit their applicability.^15^ Designs incorporating bulky groups on the squaramide core have been found to promote disaggregation,^16^ which can be alternatively forced at higher temperatures.

With the aim of evaluating self-aggregation of sulfonyl squaramides, molecular dynamics (MD) simulations were performed on **I** as a model representative.^17^ A clear preference for hydrogen-bonded structures was observed over π-stacked aggregates, in contrast with that described for *N*-methyl squaramides.^18^ Aggregation studies with dimers of **I** show that at 300 K three different aggregates might be formed in similar amounts without the minimal presence of monomers, a fact that explains the reproducibility problems observed at this temperature (see discussion below). At ca. 325 K, however, appreciable disaggregation is observed, which is again in agreement with the experimentally observed reaction reproducibility. These facts also suggest a high degree of molecular disorder, which hampered the efforts to obtain suitable crystals for XRD studies in different types of solvents.

The cyclization of *N*-propargyl benzamide **1** to oxazoline **2** was selected as a first benchmark reaction to check the performance of SO_2_Sq cocatalysts **I**–**IV**, since it has emerged as an established test case for silver-free intramolecular H-bonding activations.^7−9^ Moreover, isomeric oxazole **2**′ is reported to be obtained in the presence of Au(III) complexes or acidic additives,^19^ providing relevant warning signs of the following: (*i*) The presence of impurities in the Au(I) complexes or undesired degradation/disproportionation events during the activation process. (*ii*) The eventual action of SO_2_Sq or any other species as Brønsted acid catalysts.

A proof-of-concept was rapidly obtained using 5 mol % of both (PPh_3_)AuCl (**Au1**) as the catalyst and sulfonyl squaramide **I** as the activator in dry CH_2_Cl_2_ at 25 °C. ^1^H NMR monitoring showed a moderate conversion to oxazoline **2** after 16 h (entry 1, Table 1). In accordance with previous studies,^9^**Au1** in combination with conventional Schreiner’s urea **VI** or squaramides **VII**–**VIII** did not promote the reaction (entries 2–4), highlighting the difficulties to carry out this activation with well-established H-bond donors in intermolecular fashion. Irreproducibility issues observed in reactions carried out at 25 °C were attributed to the formation of self-aggregates, an assumption supported by the above-mentioned molecular dynamics (MD) studies. Moreover, increasing the reaction temperature up to 35 °C overcame the problem and the collected results became consistently better (entry 5). Best results were observed in reactions carried out in DCE, leading to oxazoline **2** in 98% yield in 10 h (entry 6). Ethereal solvents (Et_2_O, DME, and THF) were not suitable for this catalytic system, suggesting that coordination to the active gold center might prevent substrate activation. Other solvents such as toluene or α,α,α-trifluorotoluene (TFT) were tolerated, albeit with lower efficiency.^17^ Next, different gold complexes (**Au2**–**Au6**) were evaluated. Increasing steric hindrance on the phosphine ligand (e.g., in JohnPhos or SPhos) led to lower catalytic activities (entries 7 and 8). Phosphoramidite-based complex **Au4** was the less active among the phosphorus-ligated series (entry 9). Of note, prolonged reaction times or higher temperatures led to the formation of variable amounts of byproduct **2**′.^17^ The catalytic performance of NHC-based complexes SIPrAuCl (**Au5**) and IPrAuCl (**Au6**) were also competitive, the latter behaving similar to **Au1** (entry 11 vs entry 6). Next, the remaining sulfonyl squaramides were evaluated as activators under optimized reaction conditions. Substitution of the 3,5-bis(trifluoromethyl)phenyl group by phenyl (**II**) or 1-naphthyl (**III**) groups reasonably maintained the catalyst activation, albeit conversions were lower than that obtained with the most acidic SO_2_Sq **I** (entries 12 and 13). However, formation of oxazole **2′** (13%) was detected in the case of promoter **III** bearing the bulkiest aryl moiety. The use of 1-adamantyl-substituted activator **IV** had a marked impact in the catalytic efficiency (entry 14), revealing that a relatively strong H-bond donor ability of this position is also essential for the Au–Cl bond labilization process. The acidity of the sulfonamide fragment is also important, as revealed by the lower yield (60%) obtained by employing *p*-tosyl group-containing catalyst **V** (entry 15). In conclusion, stronger bidentate H-bond donor moieties induce higher catalytic activities, in accordance with a better stabilization of the ion pair through chloride binding by H-bonds. Importantly, the absence of oxazole **2′** in most of the cases rules out a behavior of SO_2_Sq as a Brønsted acid. It is worth noting that, in the 3,5-bis(trifluoromethyl)phenyl group, *ortho*-protons might also participate in the above-mentioned stabilization while additional noncovalent interactions (NCIs) between ligand scaffolds and aryl rings of sulfonyl squaramides might also be involved.

**Table 1:**
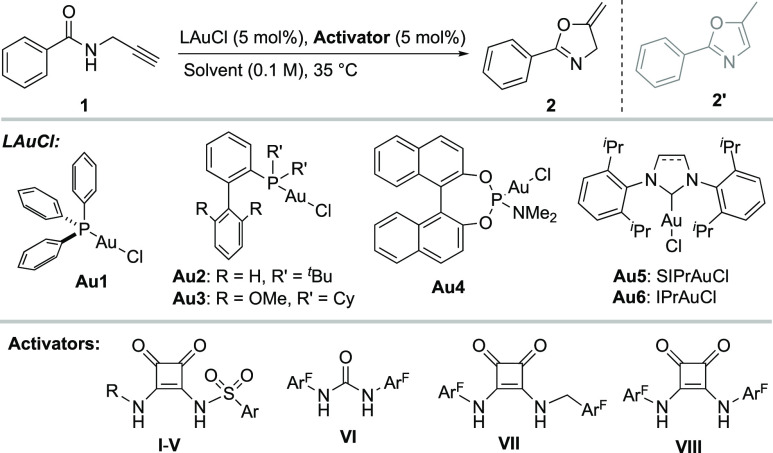
Optimization of Reaction Conditions^a^

Entry	Solvent	*T* (°C)	LAuCl	Activator	Yield (%)^b^
1	CH_2_Cl_2_	25	Au1	**I**	58
2	CH_2_Cl_2_	25	Au1	**VI**	<5
3	CH_2_Cl_2_	25	Au1	**VII**	<5
4	CH_2_Cl_2_	25	Au1	**VIII**	<5
5	CH_2_Cl_2_	35	Au1	**I**	93
6	DCE	35	Au1	**I**	>95^c^ (98)^d^
7	DCE	35	Au2	**I**	79
8	DCE	35	Au3	**I**	63
9	DCE	35	Au4	**I**	50
10	DCE	35	Au5	**I**	73
11	DCE	35	Au6	**I**	95
12	DCE	35	Au1	**II**	70
13	DCE	35	Au1	**III**	71 [13]^e^
14	DCE	35	Au1	**IV**	21
15	DCE	35	Au1	**V**	60

a Reactions were performed at 0.2 mmol scale. Reaction time: 16 h.

b Estimated by ^1^H NMR.

c Reaction time: 10 h.

d In parentheses, isolated yield after column chromatography.

e In brackets, yield of **2**′.

The performance of the designed catalytic system was further assessed in the heterocyclization/1,2-migration cascade of alkynyl carbonyl compound **3** leading to spirocyclic 3(2*H*)-furanone **4**. This is also a challenging reaction, since it has been reported that the use of cationic gold(I) or silver(I) complexes leads to significant decomposition of the starting material.^20^ Initial control experiments were conducted employing **3** at 40 °C in CH_2_Cl_2_ [0.2 M]. Importantly, neither (PPh_3_)AuCl (**Au1**) nor promoter **I** by itself independently catalyze the reaction.^17^ In agreement with literature, the use of typical chloride scavengers such as AgNTf_2_ or NaBAr^F^_4_ revealed the catalytic activity of the cationic ( PPh_3_)Au(I) complex, albeit with extensive decomposition of starting material (Table 2, entries 1 and 2). Optimal catalytic system **Au1**/**I** afforded spirocyclic compound **4** in only 22% yield, but without appreciable decomposition of **3** (entry 3). The solvent, temperature, and concentration had a marked influence on the catalytic performance. Thus, toluene provided a quite unproductive reaction,^17^ while employing DCE at higher temperatures allowed a progressive increase in the yield of **4** (entries 4 and 5), with a maximum of 81% at 70 °C. To our delight, dilution at 0.03 M improved the yield up to 98% (entry 6). Interestingly, SIPrAuCl (**Au5**) and IPrAuCl (**Au6**) were also competent gold(I) precatalysts (entries 7 and 8). Employing sulfonyl squaramide **II**, a significant drop of catalytic activity was observed (entries 9 and 10), thereby highlighting again the essential role of the NH-donor ability of the activator. The catalyst loading could be reduced to 2.5 mol % without compromising the chemical yield. To further assess the usefulness of the optimal catalytic system, spirocycle **4** was prepared in 93% yield at 1 mmol scale.^17^

**Table 2:**
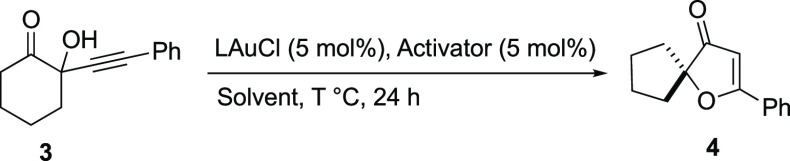
Optimization of Reaction Conditions^a^

Entry	Solvent [M]	*T* (°C)	LAuCl	Activator	**4** (%)^b^	**3** (%)^b^
1	CH_2_Cl_2_ [0.2]	40	Au1	AgNTf_2_	66	<5
2	CH_2_Cl_2_ [0.2]	40	Au1	NaBAr^F^_4_	49	<5
3	CH_2_Cl_2_ [0.2]	40	Au1	I	22	73
4	DCE [0.2]	50	Au1	I	37	52
5	DCE [0.2]	70	Au1	I	81	8
6	DCE [0.03]	70	Au1	I	>95 (98)^c^	<5
7	DCE [0.03]	70	Au5	I	92	<5
8	DCE [0.03]	70	Au6	I	>95 (98)^c^	<5
9	DCE [0.03]	70	Au1	II	11	89
10	DCE [0.03]	70	Au5	II	13	83

a Reactions were performed at 0.2 mmol scale.

b Yields of **4** and unreacted **3** estimated by ^1^H NMR.

c In parentheses, isolated yield after column chromatography.

**Scheme 2:**
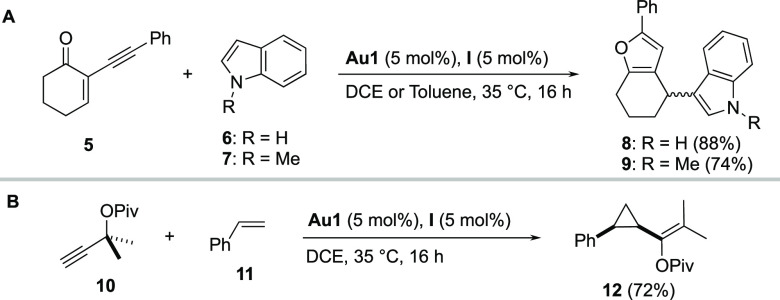
(A) Tandem Cycloisomerization/Nucleophilic Addition; (B) Intermolecular Cyclopropanation Reactions were performed at 0.2 mmol scale. Isolated yields after column chromatography.

The optimized combination of **Au1** and **I** was also catalytically competent, in either DCE or toluene, in the tandem cycloisomerization/nucleophilic addition reaction of 2-alkynylenone **5** (Scheme 2A).^21^ Indole (**6**) and *N*-methyl-indole (**7**) were tested as nucleophiles, affording the furans **8** and **9** in 88% and 74% yield, respectively. Finally, we performed a challenging intermolecular cyclopropanation employing propargyl pivalate (**10**) and styrene (**11**) (Scheme 2B).^22^ Satisfactorily, product **12** was obtained in 72% yield (*cis*/*trans*, 7:1). This last result reinforces the potential applicability of this intermolecular activation, beyond intramolecular cyclization processes.

**Scheme 3:**
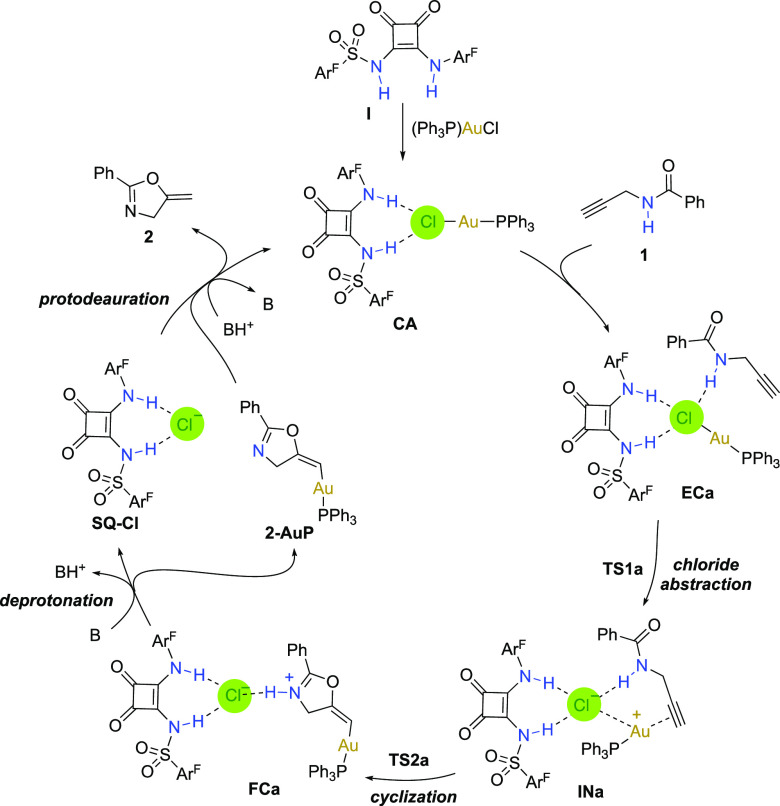
Catalytic Cycle for the Cyclization of *N*-Propargyl Benzamide **1** to Oxazoline **2**

Computational studies were performed to shed light over the actual catalytic system. For the reaction of **1** to give **2** the catalytic cycle illustrated in Scheme 3 is proposed. Initially, the squaramide **I** binds to (PPh_3_)AuCl forming the complex **CA**. Incorporation of amide **1** yields the starting encounter complex **ECa**. Next, a reactive intermediate **INa** is formed through a transition state **TS1a** with an energy barrier of 3.0 kcal/mol (7.1 kcal/mol from **ECa**). In this intermediate the Au–Cl bond could be considered broken, even though an interaction between both atoms still remains (see below). This value is very similar to that reported by Echavarren and co-workers (6.7 kcal/mol) for the similar activation in intramolecular fashion.^9^ These authors, however, did not locate the transition structure corresponding to the cyclization. In our case, that transition structure (**TS2a**) showed a barrier of 10.5 kcal/mol to form the final complex **FCa**. Next, the catalytic cycle follows deprotonation and protodeauration steps to give the product **2**. These data point to the cyclization step as the rate determining stage, providing the squaramide is disaggregated. Starting from the aggregated squaramide, the disaggregation step should be the rate-limiting stage since ca. 35 °C is required. At that temperature, the barrier of 10.5 kcal/mol found for the cyclization step would be amply surpassed.^23^

**Figure 2:**
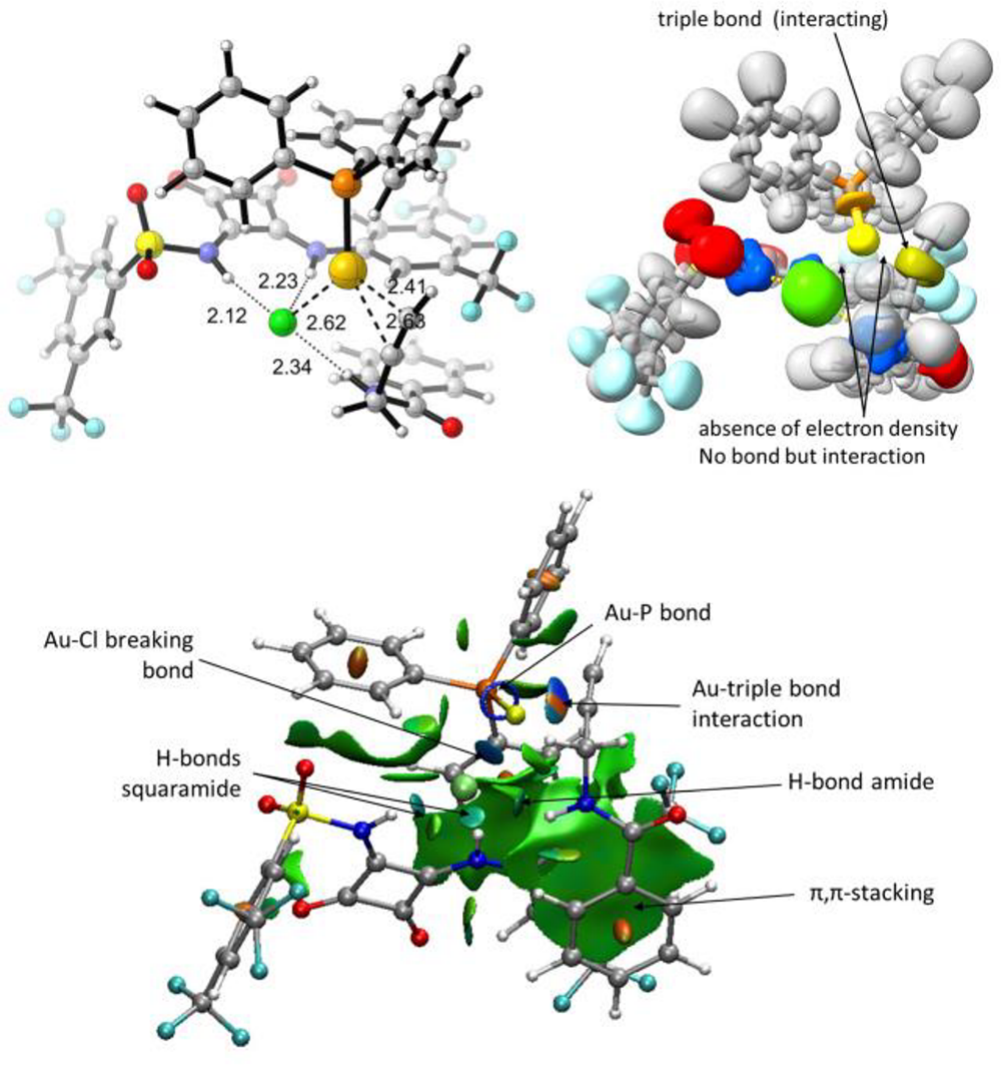
Transition strucure **TS1a** for the reaction of **1**. Top left: Optimized (b3lyp-gd3bj/def2spv/SMD = DCE) geometry. Top right: ELF analysis. Au is colored in yellow, chlorine in green, nitrogen in blue and oxygens in red. Note the typical toroidal form for a basin corresponding to a triple bond. Bottom: NCI analysis showing main noncovalent interactions. Green area corresponds to weak van der Waals interactions. Blue area corresponds to strong interactions and red area corresponds to repulsive forces.

**Figure 3:**
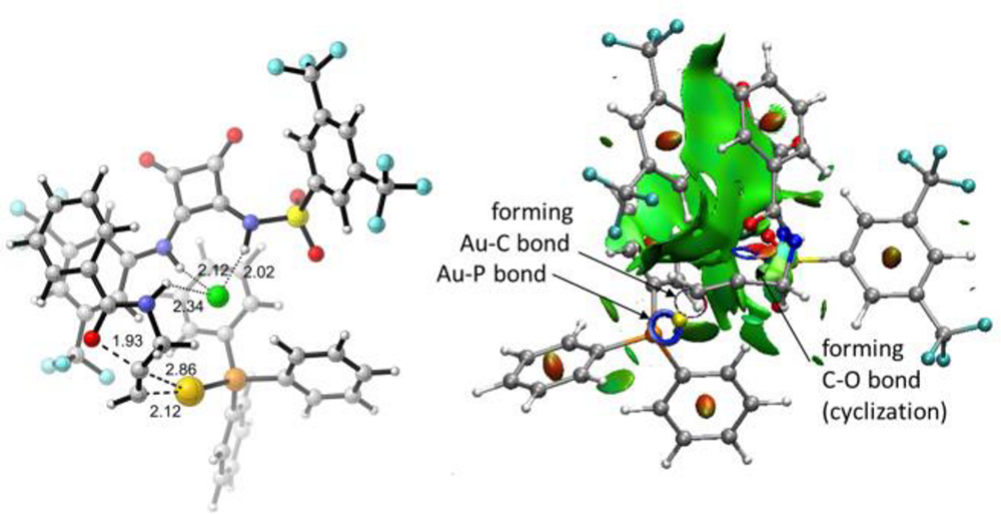
Transition strucure **TS2a** for the reaction of **1**. Left: Optimized (b3lyp-gd3bj/def2spv/SMD = DCE) geometry. Right: NCI analysis showing main noncovalent interactions.

Transition structure **TS1a** corresponds to the abstraction of the chloride to yield an intermediate in which the Au is forming a complex with the triple bond which remains as such, as confirmed by an analysis of the electron localization function (Figure 2, top).^24^ The chloride anion is coordinated by the squaramide and an additional H-bond of the amide, rendering a situation similar to that described in the intramolecular approach since the complex squaramide-chloride remains close to the reaction center and some interaction gold–chloride is still present as confirmed by NCI analysis (Figure 2, bottom).^25^ In fact, such an interaction can be also observed in **INa** (distance Au···Cl = 2.7 Å). Transition structure **TS2a** corresponds to the cyclization leading to the oxazoline moiety, and at this stage there are no Au–Cl interactions of any type as corroborated by the NCI analysis (Figure 3). These results suggest that the abstraction of the chloride in the first step is partial, and it is only completed after the second transition structure when complex **FCa** is liberated. We also calculated the transformation of **3** into **4**. In this reaction, a very similar catalytic cycle is proposed.^17^ However, after heterocyclization, an additional 1,2-migration would be necessary, this step being the rate determining stage of the process.^23^

## Conclusions

In summary, readily available sulfonyl squaramides are competent cocatalysts for the challenging intermolecular activation of Au(I) chloride complexes through H-bonding, providing an appealing approach to silver-free Au(I) catalysis. In accordance with experimental and computational studies, the superior acidity delivered by a 3,5-bis(trifluoromethyl)phenyl sulfonyl group overcomes the entropic cost of this intermolecular activation. On the basis of these findings, introduction of chiral fragments into SO_2_Sq designs for the development of enantioselective reactions through Au(I)/ion-pairing strategies is currently under investigation in our laboratories.

## Data Availability

The data underlying this study are available in the published article and its Supporting Information.

## Supporting Information Available

Experimental procedures, optimization experiments, characterization data, NMR spectra for new compounds, and computational studies (PDF)
FAIR data, including the primary NMR FID files, for compounds **I**–**V**, **VII**, **P2**, and **P3** (ZIP)

## References

[ref1] aRudolph M.; Hashmi A. S. K. Heterocycles from Gold Catalysis. Chem. Commun. 2011, 47, 6536–6544. 10.1039/c1cc10780a. 21451867

[ref2] aWeber D.; Gagne M. R. Dinuclear Gold–Silver Resting States May Explain Silver Effects in Gold(I)-Catalysis. Org. Lett. 2009, 11, 4962–4965. 10.1021/ol902116b. 19807117 PMC2913295

[ref3] Franchino A.; Montesinos-Magraner M.; Echavarren A. M. Silver-Free Catalysis with Gold(I) Chloride Complexes. Bull. Chem. Soc. Jpn. 2021, 94, 1099–1117. 10.1246/bcsj.20200358.

[ref4] Guérinot A.; Fang W.; Sircoglou M.; Bour C.; Bezzenine-Lafollée S.; Gandon V. Copper Salts as Additives in Gold(I)-Catalyzed Reactions. Angew. Chem., Int. Ed. 2013, 52, 5848–5452. 10.1002/anie.201300600. 23606499

[ref5] Tzouras N. V.; Gobbo A.; Pozsoni N. B.; Chalkidis S. G.; Bhandary S.; Van Hecke K.; Vougioukalakis G. C.; Nolan S. P. Hydrogen bonding-enabled gold catalysis: ligand effects in gold-catalyzed cycloisomerizations in hexafluoroisopropanol. Chem. Commun. 2022, 58, 8516–8519. 10.1039/D2CC03056J. 35801509

[ref6] aWolf J.; Huber F.; Erochok N.; Heinen F.; Guérin V.; Legault C. Y.; Kirsch S. F.; Huber S. M. Activation of a Metal-Halogen Bond by Halogen Bonding. Angew. Chem., Int. Ed. 2020, 59, 16496–16500. 10.1002/anie.202005214. PMC7540446 32472957

[ref7] Sen S.; Gabbaï F. P. An Ambiphilic Phosphine/H-Bond Donor Ligand and Its Application to the Gold Mediated Cyclization of Propargylamides. Chem. Commun. 2017, 53, 13356–13358. 10.1039/C7CC06065C. 29199294

[ref8] Seppänen O.; Aikonen S.; Muuronen M.; Alamillo-Ferrer C.; Burés J.; Helaja J. Dual H-Bond Activation of NHC-Au(I)-Cl Complexes with Amide Functionalized Side-Arms Assisted by H-Bond Donor Substrates or Acid Additives. Chem. Commun. 2020, 56, 14697–14700. 10.1039/D0CC05999D. 33169740

[ref9] Franchino A.; Martí À.; Nejrotti S.; Echavarren A. M. Silver-Free Au(I) Catalysis Enabled by Bifunctional Urea- and Squaramide-Phosphine Ligands via H-Bonding. Chem.—Eur. J. 2021, 27, 11989–11996. 10.1002/chem.202101751. 34018646 PMC8457243

[ref10] Auvil T. J.; Schafer A. G.; Mattson A. E. Design Strategies for Enhanced Hydrogen-Bond Donor Catalysts. Eur. J. Org. Chem. 2014, 2014, 2633–2646. 10.1002/ejoc.201400035.

[ref11] Li Y.; Yang G.-H.; Shen Y.-Y.; Xue X.-S.; Li X.; Cheng J.-P. *N*-*tert*-Butyl Sulfinyl Squaramide Receptors for Anion Recognition through Assisted *tert-*Butyl C-H Hydrogen Bonding. J. Org. Chem. 2017, 82, 8662–8667. 10.1021/acs.joc.7b01634. 28768098

[ref12] Molodtsov V.; Fleming P. R.; Eyermann C. J.; Ferguson A. D.; Foulk M. A.; McKinney D. C.; Masse C. E.; Buurman E. T.; Murakami K. S. X-ray Crystal Structures of *Escherichia coli* RNA Polymerase with Switch Region Binding Inhibitors Enable Rational Design of Squaramides with an Improved Fraction Unbound to Human Plasma Protein. J. Med. Chem. 2015, 58, 3156–3171. 10.1021/acs.jmedchem.5b00050. 25798859 PMC4658208

[ref13] aLi Y.; He C. O.; Gao F.-X.; Li Z.; Xue X.-S.; Li X.; Houk K. N.; Cheng J.-P. Design and Applications of *N*-*tert*-Butyl Sulfinyl Squaramide Catalysts. Org. Lett. 2017, 19, 1926–1929. 10.1021/acs.orglett.7b00727. 28357868

[ref14] Lu M.; Lu Q.-B.; Honek J. F. Squarate-based carbocyclic nucleosides: Syntheses, computational analyses and anticancer/antiviral evaluation. Bioorg. Med. Chem. Lett. 2017, 27, 282–287. 10.1016/j.bmcl.2016.11.058. 27913181

[ref15] Marchetti L. A.; Kumawat L. K.; Mao N.; Stephens J. C.; Elmes R. B. P. The Versatility of Squaramides: From Supramolecular Chemistry to Chemical Biology. Chem. 2019, 5, 1398–1485. 10.1016/j.chempr.2019.02.027.

[ref16] aLee T. J.; Ryu W. H.; Oh J. S.; Bae H. Y.; Jang H. B.; Song C. E. Self-association-free dimeric cinchona alkaloid organocatalysts: unprecedented catalytic activity, enantioselectivity and catalyst recyclability in dynamic kinetic resolution of racemic azlactones. Chem. Commun. 2009, 7224–7226. 10.1039/b917882a 19921037

[ref17] See the Supporting Information for details.

[ref18] Sen S.; Basu A.; Sen T.; Patwari G. N. π-Stacking Driven Aggregation and Folding of Squaramides. J. Phys. Chem. A 2020, 124, 5832–5839. 10.1021/acs.jpca.0c03120. 32530630

[ref19] aHashmi A. S. K.; Schuster A. M.; Rominger F. Gold Catalysis: Isolation of Vinylgold Complexes Derived from Alkynes. Angew. Chem., Int. Ed. 2009, 48, 8247–8249. 10.1002/anie.200903134. 19768822

[ref20] Kirsch S. F.; Binder J. T.; Liébert C.; Menz H. Gold(III) and Platinum(II)-Catalyzed Domino Reaction Consisting of Heterocyclization and 1,2-Migration: Efficient Synthesis of Highly Substituted 3(2*H*)-furanones. Angew. Chem., Int. Ed. 2006, 45, 5878–5880. 10.1002/anie.200601836. 16871607

[ref21] aZhang Z.; Smal V.; Retailleau P.; Voituriez A.; Frison G.; Marinetti A.; Guinchard X. Tethered Counterion-Directed Catalysis: Merging the Chiral Ion-Pairing and Bifunctional Ligand Strategies in Enantioselective Gold(I) Catalysis. J. Am. Chem. Soc. 2020, 142, 3797–3805. 10.1021/jacs.9b11154. 32011877

[ref22] Johansson M. J.; Gorin D. J.; Staben S. T.; Toste F. D. Gold(I)-Catalyzed Stereoselective Olefin Cyclopropanation. J. Am. Chem. Soc. 2005, 127, 18002–18003. 10.1021/ja0552500. 16366541

[ref23] For the full energy profile, also see the Supporting Information.

[ref24] aBecke A. D.; Edgecombe K. E. A simple measure of electron localization in atomic and molecular systems. J. Chem. Phys. 1990, 92, 5397–5403. 10.1063/1.458517.

[ref25] aJohnson E. R.; Keinan S.; Mori-Sanchez P.; Contreras-Garcia J.; Cohen A. J.; Yang W. Revealing Noncovalent Interactions. J. Am. Chem. Soc. 2010, 132, 6498–6506. 10.1021/ja100936w. 20394428 PMC2864795

