# Supplementary material for: Silver-Free Gold-Catalyzed Heterocyclizations through Intermolecular H-Bonding Activation
Source: J Org Chem. 2023 Jan 27;88(4):2487–92. doi: 10.1021/acs.joc.2c02932 (PMC9942198; doi:10.1021/acs.joc.2c02932)
Supplement: Supplementary file 1 — jo2c02932_si_001.pdf [file jo2c02932_si_001.pdf]

Supporting information

Silver-Free Gold-Catalyzed Heterocyclizations through Intermolecular H-Bonding Activation

Pilar Elías-Rodríguez,[†] Esteban Matador,[†] Manuel Benítez,[†] Tomás Tejero,[§] Elena Díez,[†] Rosario Fernández,*[,†] Pedro Merino,*[,§] David Monge*[,†] and José M. Lassaletta,*[,‡]

† Facultad de Química. Departamento de Química Orgánica. Universidad de Sevilla and Centro de Innovación en Química Avanzada (ORFEO-CINQA), C/Prof. García González, 1, 41012 Sevilla, Spain

§ Instituto de Biocomputación y Física de Sistemas Complejos (BIFI). Universidad de Zaragoza-CSIC, 50009 Zaragoza, Spain

‡ Instituto de Investigaciones Químicas (CSIC-US) and Centro de Innovación en Química Avanzada (ORFEO-CINQA), Avda. Américo Vespucio, 49, 41092 Sevilla, Spain

[*]Corresponding authors. E-mails: ffernan@us.es (R.F.), pmerino@unizar.es (P.M.), dmonge@us.es (D.M.), jmlassa@iiq.csic.es (J.M.L.)

Table of Contents

1. General information…………………………………………………………………….S1
2. Synthesis of compounds P1, P2 and P3…………………………………………………S1
3. Synthesis of organocatalysts I-III, V……………………………………………………S3
4. Synthesis of organocatalyst IV…………………………………………………………….S5
5. Synthesis of organocatalyst VII…………………………………………………………S6
6. Cyclization of *N*-propargyl benzamide…………………………………………………S7
6.1. Optimization of the reaction parameters……………………………………………….S7
6.2. General procedure for cyclization of *N*-propargyl benzamide………………………......S9
7. Heterocyclization and subsequent 1,2-alkyl shift of α-hydroxy α -alkynyl ketone……......S9
7.1. Optimization of the reaction parameters…………………………………………………S9
7.2. General procedure for heterocyclization and subsequent 1,2-alkyl shift of α-hydroxy α -alkynyl ketone at 1mmol scale reaction……………………………………………..S10
8. General procedure for tandem cycloisomerization and nucleophilic addition……….......S11
9. General procedure for intermolecular olefin cyclopropanation…………………………….S12
10. Computational studies………………………………………………………………..S13
10.1. Computational methods……………………………………………………….......S13
10.2. MD simulations…………………………………………………………………………..S14
10.3. Catalytic cycles…………………………………………………………………….......S16
10.4. Optimized geometries……………………………………………………………………S18
10.5. Topological analyses………………………………………………………………..S19
10.6. Energies……………………………………………………………………………..S21
10.7. Cartesian Coordinates……………………………………………………….......S23
11. References…………………………………………………………………………..S72
12. NMR spectra of new compounds…………………………………………………………S76

1. General information

Unless otherwise stated, all reactions were performed in dry glassware under nitrogen or argon atmosphere using standard Schlenk techniques. THF and toluene were distilled over Na/benzophenone, CH_2Cl_2 was distilled from $CaCl_2$, and 1,2-DCE was taken from commercial bottle equipped with septa and molecular sieves. Other analytical grade solvents were used without further purification. All chemicals were purchased from Sigma-Aldrich, Across Organics, TCI, and Fluorochem and used as received. Purifications by column chromatography were performed on silica gel (Merck Kieselgel 60). Analytical TLC was performed on aluminium backed plates (1.5 × 5 cm) pre-coated (0.25 mm) with silica gel (Merck, Silica Gel 60 F254). Compounds were visualized by exposure to UV light at 254 nm and/or by dipping the plates in solutions of $KMnO_4$, vanillin or phosphomolybdic acid stains followed by heating. ^{1}H, ^{13}C and ^{19}F NMR spectra were collected on a Bruker Avance NEO 300 MHz and Bruker Avance NEO 500 MHz spectrometers at room temperature. ^{1}H NMR spectra were recorded at 300 MHz or 500 MHz (internal reference; $CDCl_3$ = 7.26 ppm; DMSO-d_6 = 2.50 ppm). $^{13}C\{1H\}$ NMR spectra were recorded at 75.5 or 126 MHz (internal reference; $CDCl_3$ = 77.0 ppm; DMSO-d_6 = 39.5 ppm); ^{19}F NMR spectra were recorded at 471 MHz. Chemical shifts are expressed in parts per million (ppm, δ scale) and coupling constants are in hertz (Hz). Multiplicities were given as: s (singlet), br s (broad singlet), d (doublet), dd (double doublet), t (triplet), q (quartet), and m (multiplet). All the assignments were confirmed by COSY and HSQC experiments. Optical rotations were measured on a JASCO *P-2000* polarimeter. High resolution mass spectra were recorded on a Q-Exactive spectrometer by a positive electrospray ionization (ESI) method using a Quadrupole-Orbitrap hybrid analyzer. Not commercially available substrates **1**,[1] **3**,[2] **5**,[3] **10**,[4] activators **VI**,[5] **VIII**,[6] and gold(I) chloride complexes **Au2**,[7] **Au3**,[8] **Au4**,[9] **Au5**,[10] **Au6**,[10] **Au7**,[11] were synthetized according to literature procedures.

2. Synthesis of compounds P1, P2 and P3

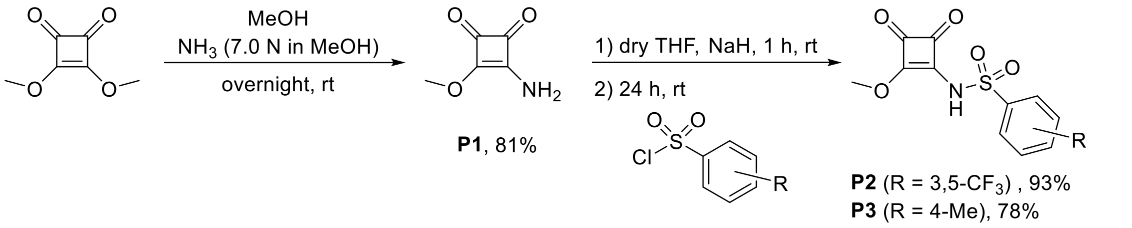

Step 1. Ammonia (0.71 mL, 7.0 N solution in MeOH) was added dropwise to a solution of 3,4-dimethoxycyclobut-3-ene-1,2-dione (0.73 g, 5.0 mmol) in MeOH (5.0 mL, 1 M) at room temperature.

The resulting mixture was stirred at the same temperature overnight. Then, the precipitated formed was filtered and washed with MeOH (2 x 5 mL) to afford 3-amino-4-methoxycyclobut-3-ene-1,2-dione (**P1**) as a white solid (514 mg, 4.04 mmol, 81%). **^{1}H NMR** (300 MHz, DMSO-d_6): δ 8.28 (br s, 2H), 4.26 (s, 3H). The experimental data are in accordance with those reported in literature.[12]

Step 2. *General procedure:* NaH powder (129 mg, 4.84 mmol) was added to a solution of 3-amino-4-methoxycyclobut-3-ene-1,2-dione (**P1**) (0.30 g, 2.36 mmol) in dry THF (9.5 mL, 0.25 M) at room temperature. The resulting suspension was stirred at the same temperature for 1 hour and then, the corresponding sulfonyl chloride (3.07 mmol) was added in one portion. The reaction was stirred until the consumption of the starting material (24 h, TLC monitoring). Then, the solvent was eliminated under reduced pressure and EtOAc (9.5 mL), and HCl (3.30 mL, 1.0 M in Et_2O) were subsequently added. The resulting yellow suspension was diluted with EtOAc (100 mL), washed with brine (2 x 50 mL), dried over $MgSO_4$ and concentrated under reduced pressure. The resulting residue was purified by column chromatography on silica gel (EtOAc/acetone 4/1) to afford the desired product.

Benzenesulfonamide P2

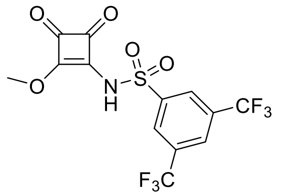

Following the general procedure (step 2), starting from 3,5-bis(trifluoromethyl)benzenesulfonyl chloride (0.99 g, 2.45 mmol, 3.07 mmol), compound **P2** was obtained as a pale orange solid (0.89 g, 2.21 mmol, 93%). **^{1}H NMR** (300 MHz, DMSO-d_6): δ 8.42 (s, 2H), 8.30 (s, 1H), 6.99 (br s, 1H), 4.18 (s, 3H). **^{13}C{1H} NMR** (75.5 MHz, DMSO-d_6): δ 193.0, 187.9, 184.4, 177.3, 146.9, 130.8 (q, $J_{C,F}$ = 33.6 Hz), 127.7 (d, $J_{C,F}$ = 3.3 Hz), 125.8 − 125.3 (m), 122.9 (q, $J_{C,F}$ = 273.3 Hz), 59.6. **^{19}F NMR** (471 MHz, DMSO-d_6): δ −61.45 (s, 6F). **HRMS** (ESI): *m/z* calcd for $C_{13}H_7O_5NF_6NaS$ [M$^+$+Na] 425.9841, found 425.9837.

Benzenesulfonamide P3

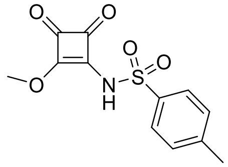

Following the general procedure (step 2), starting from *p*-toluenesulfonyl chloride (0.60 g, 3.07 mmol), compound **P3** was obtained as a yellow solid (0.52 g, 1.85 mmol, 78%). **^{1}H NMR** (500 MHz,

DMSO-d_6): δ 7.69 (d, *J* = 8.2 Hz, 2H), 7.24 (d, *J* = 8.0 Hz, 2H), 4.16 (s, 3H), 2.32 (s, 3H). **^{13}C{1H} NMR** (126 MHz, DMSO-d_6): δ 195.0, 187.3, 183.6, 179.1, 142.2, 140.4, 128.7, 126.5, 58.9, 20.9. **HRMS** (ESI): *m/z* calcd for $C_{12}H_{11}O_5NNaS$ [M^++Na] 304.0250, found 304.0251.

3. Synthesis of organocatalysts I-III, V

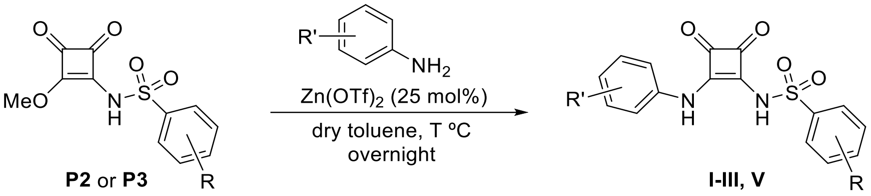

General procedure: In a flame-dried schlenk flask, the corresponding aniline derivative (0.9 mmol) was added to a suspension of the corresponding precursor **P2-P3** (0.5 mmol), and zinc(II) trifluoromethanesulfonate (46 mg, 0.125 mmol, 25 mol%) in dry toluene (2.0 mL, 0.25 M) at room temperature. The resulting mixture was stirred at the temperature specified for each substrate overnight. Then, the solvent was eliminated under reduced pressure and the resulting residue was purified by column chromatography on silica gel to afford organocatalysts **I-III** and **V**.

***N*-{2-{[3,5-Bis(trifluoromethyl)phenyl]amino}-3,4-dioxocyclobut-1-en-1-yl}-3,5 bis(trifluoromethyl)benzenesulfonamide (I)**

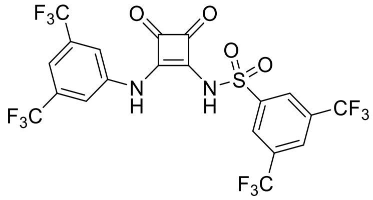

Following the general procedure at 100 ºC (sand bath), starting from 3,5-bis(trifluoromethyl)aniline (145 µL, 0.9 mmol) and **P2** (202 mg, 0.5 mmol), compound **I** was obtained as a pale-orange solid (265 mg, 0.44 mmol, 88%; **Reaction performed at 2.0 mmol scale:** 931 mg, 1.55 mmol, 78%). **^{1}H NMR** (500 MHz, DMSO-d_6): δ 10.34 (s, 1H), 8.52 (s, 2H), 8.28 (s, 3H), 7.52 (s, 1H). **^{13}C{1H} NMR** (126 MHz, DMSO-d_6): δ 188.5, 185.2, 177.0, 170.2, 147.9, 141.8, 131.0 (q, $J_{C,F}$ = 32.7 Hz), 130.6 (q, $J_{C,F}$ = 33.6 Hz), 127.6 (d, $J_{C,F}$ = 3.1 Hz), 124.6 − 124.5 (m), 123.2 (q, $J_{C,F}$ = 272.7 Hz), 122.9 (q, $J_{C,F}$ = 273.2 Hz), 118.2 (d, $J_{C,F}$ = 3.2 Hz), 113.9 − 113.8 (m). **^{19}F NMR** (471 MHz, DMSO-d_6): δ −61.51 (s, 6F), −61.80 (s, 6F). **HRMS** (ESI): *m/z* calcd for $C_{20}H_8O_4N_2F_{12}NaS$ [M^++Na] 622.9905, found 622.9896.

***N*-[3,4-Dioxo-2-(phenylamino)cyclobut-1-en-1-yl]-3,5-bis(trifluoromethyl)benzene-sulfonamide (II)**

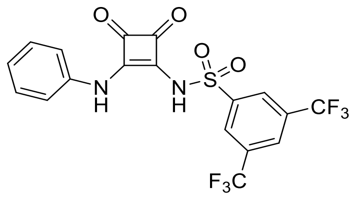

Following the general procedure at 60 ºC (sand bath), starting from aniline (83 µL, 0.9 mmol) and **P2** (202 mg, 0.5 mmol), compound **II** was obtained as a brown solid (225 mg, 0.48 mmol, 97%). **^{1}H NMR** (500 MHz, DMSO-d_6): δ 9.75 (s, 1H), 8.54 (s, 2H), 8.25 (s, 1H), 7.55 (d, J = 7.8 Hz, 2H), 7.24 (t, J = 7.8 Hz, 2H), 6.93 (t, J = 7.8 Hz, 1H). **^{13}C{1H} NMR** (126 MHz, DMSO-d_6): δ 187.9, 185.4, 176.2, 171.4, 148.3, 139.6, 130.6 (q, $J_{C,F}$ = 33.3 Hz), 128.8, 127.6 – 127.5 (m), 124.4 – 124.3 (m), 122.9 (q, $J_{C,F}$ = 273.3 Hz), 122.0, 118.4. **^{19}F NMR** (471 MHz, DMSO-d_6): δ −61.44 (s, 6F). **HRMS** (ESI): *m/z* calcd for $C_{18}H_{10}O_4N_2F_6NaS$ [M$^+$+Na] 487.0158, found 487.0150.

***N*-[2-(Naphthalen-1-ylamino)-3,4-dioxocyclobut-1-en-1-yl]-3,5-bis(trifluoromethyl) benzenesulfonamide (III)**

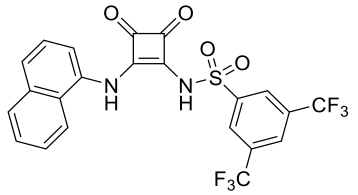

Following the general procedure at 60 ºC (sand bath), starting from naphthalen-1-amine (129 mg, 0.9 mmol) and **P2** (202 mg, 0.5 mmol), compound **III** was obtained as a brown solid (246 mg, 0.48 mmol, 96%). **^{1}H NMR** (500 MHz, DMSO-d_6): δ 10.01 (s, 1H), 8.49 (s, 2H), 8.30 (s, 1H), 8.08 (d, J = 7.5 Hz, 1H), 7.94 – 7.92 (m, 1H), 7.67 (d, J = 7.5 Hz, 1H), 7.57 – 7.52 (m, 2H), 7.48 – 7.43 (m, 2H). **^{13}C{1H} NMR** (126 MHz, DMSO-d_6): δ 189.8, 185.9, 175.5, 171.8, 148.0, 133.6, 130.6 (q, $J_{C,F}$ = 33.5 Hz), 128.1, 127.2 (d, $J_{C,F}$ = 2.9 Hz), 126.1, 125.9, 125.6, 124.6 – 124.5 (m), 123.8, 122.8 (q, $J_{C,F}$ = 262.5 Hz), 122.0, 118.3. **^{19}F NMR** (471 MHz, DMSO-d_6): δ −61.44 (s, 6F). **HRMS** (ESI): *m/z* calcd for $C_{22}H_{12}O_4N_2F_6NaS$ [M$^+$+Na] 537.0314, found 537.0309.

***N*-{2-{[3,5-Bis(trifluoromethyl)phenyl]amino}-3,4-dioxocyclobut-1-en-1-yl}-4-methyl-benzenesulfonamide (V)**

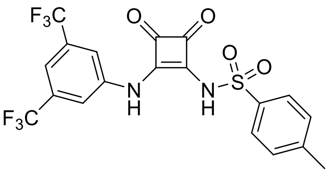

Following the general procedure at 100 ºC (sand bath), starting from 3,5-bis(trifluoromethyl)aniline (145 µL, 0.9 mmol) and **P3** (141 mg, 0.5 mmol), compound **V** was obtained as a brown solid (157 mg, 65%). **^{1}H NMR** (500 MHz, DMSO-d_6): δ 10.12 (s, 1H), 8.31 (s, 2H), 7.78 (d, J = 8.2 Hz, 2H), 7.49 (s, 1H), 7.25 (d, J = 8.0 Hz, 2H), 2.32 (s, 3H). **^{13}C{1H} NMR** (126 MHz, DMSO-d_6): δ 189.3, 184.4, 177.2, 168.8, 142.2, 142.1, 140.6, 131.0 (q, $J_{C,F}$ = 32.6 Hz), 128.8, 126.5, 123.3 (q, $J_{C,F}$ = 272.8 Hz), 117.9 (d, $J_{C,F}$ = 3.3 Hz), 113.4 − 113.3 (m), 20.8. **^{19}F NMR** (471 MHz, DMSO-d_6): δ −61.72 (s, 6F). **HRMS** (ESI): *m/z* calcd for $C_{19}H_{12}O_4N_2F_6NaS$ [M$^+$+Na] 501.0314, found 501.0317.

4. Synthesis of organocatalyst IV

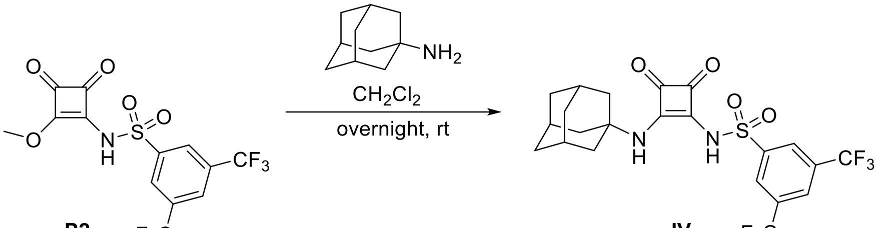

P2 IV

1-Adamanthyl amine (135 mg, 0.9 mmol) was added to a suspension of precursor **P2** (121 mg, 0.3 mmol) in CH_2Cl_2 (4.5 mL, 0.07 M) at room temperature. The resulting mixture was stirred at the same temperature overnight. Then, H_2O was added (4.5 mL), the mixture was acidified with a saturated solution of $NaHSO_4$ until pH 1-2, and extracted with CH_2Cl_2 (2 x 10 mL). The combined organic layers were washed with H_2O (1 x 15 mL), dried over $MgSO_4$ and concentrated under reduced pressure. The resulting residue was purified by column chromatography on silica gel (EtOAc/acetone 4/1) to afford **IV** as an off-white solid (127 mg, 0.24 mmol, 81%). **^{1}H NMR** (500 MHz, DMSO-d_6): δ 8.41 (s, 2H), 8.24 (s, 1H), 7.42 (s, 1H), 2.03 (s, 3H), 1.91 (s, 6H), 1.60 (s, 6H).**^{13}C{1H} NMR** (126 MHz, DMSO-d_6): δ 187.1, 186.4, 174.7, 174.1, 148.5 – 148.4 (m), 130.6 (q, $J_{C,F}$ = 33.7 Hz), 127.1 (d, $J_{C,F}$ = 2.4 Hz), 124.3 – 124.0 (m), 122.9 (q, $J_{C,F}$ = 273.1 Hz), 51.7, 42.5, 35.4, 28.9. **^{19}F NMR** (471 MHz, DMSO-d_6): δ −61.44 (s, 6F). **HRMS** (ESI): *m/z* calcd for $C_{22}H_{20}O_4N_2F_6NaS$ [M$^+$+Na] 545.0940, found 545.0937.

5. Synthesis of organocatalyst VII

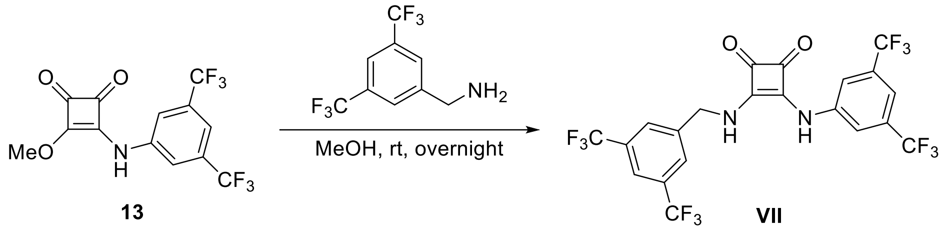

[3,5-Bis(trifluoromethyl)phenyl]methanamine (205 mg, 0.8 mmol) was added to a suspension of **13**[13] (271 mg, 0.8 mmol) in MeOH (4 mL, 0.2 M) at room temperature. The resulting mixture was stirred at the same temperature overnight. Then, the precipitated formed was filtered and washed with a cold mixture of pentane/Et_2O (1/1, 2 x 5 mL) to afford **VII** as a white solid (351 mg, 0.64 mmol, 80%). **^{1}H NMR** (500 MHz, DMSO-d_6): δ 10.29 (br s, 1H), 8.13 (s, 3H), 8.07 (s, 1H), 7.99 (s, 2H), 7.66 (s, 1H), 5.01 (d, J = 2.7 Hz, 2H). **^{13}C{1H} NMR** (126 MHz, DMSO-d_6): δ 185.0, 181.1, 169.4, 163.0, 141.9, 140.9, 131.2 (q, $J_{C,F}$ = 32.6 Hz), 130.5 (q, $J_{C,F}$ = 32.8 Hz), 128.7 (d, $J_{C,F}$ = 2.8 Hz), 123.3 (q, $J_{C,F}$ = 272.8 Hz), 123.1 (q, $J_{C,F}$ = 272.9 Hz), 121.4 – 121.3 (m), 118.4 (br s), 114.9 (br s), 46.2. **^{19}F NMR** (471 MHz, DMSO-d_6): δ −61.28 (s, 6F), −61.77 (s, 6F). **HRMS** (ESI): *m/z* calcd for $C_{21}H_{10}O_2N_2F_{12}NaS$ [M^++Na] 573.0443, found 573.0437.

6. Cyclization of *N*-propargyl benzamide

6.1. Optimization of reaction conditions

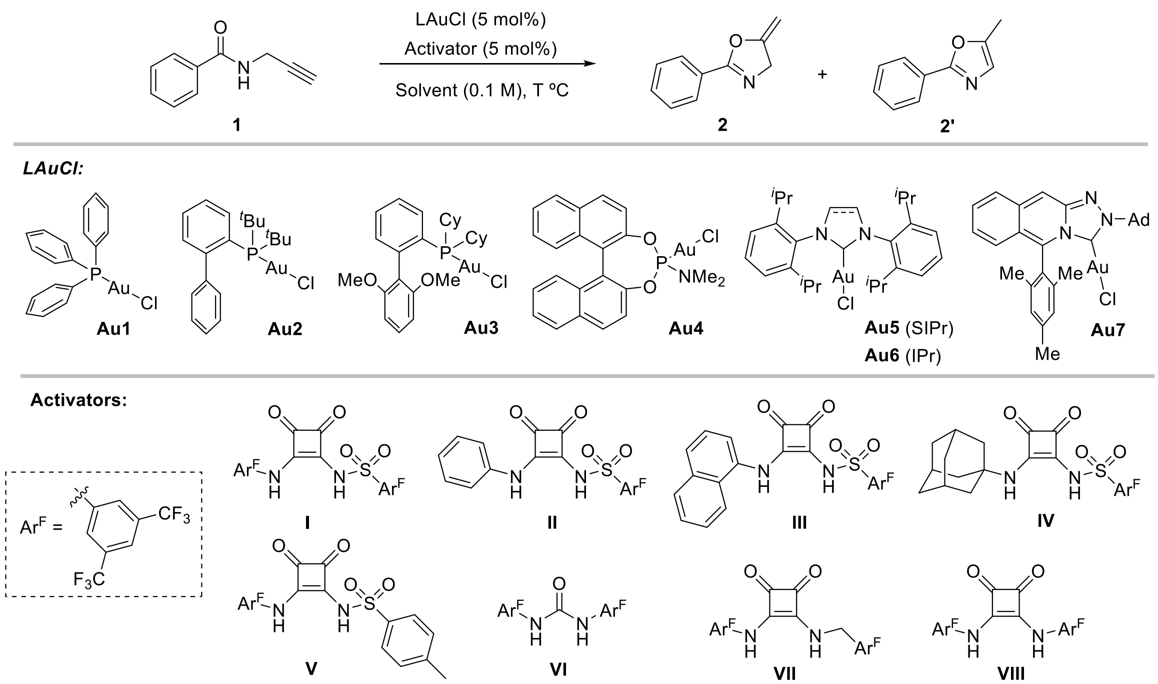

Scheme S1. Gold(I) chloride complexes and activators tested in cyclization of *N*-propargyl benzamide **1**.

In a flame-dried schlenk tube, the corresponding freshly distilled anhydrous solvent (2.0 mL, 0.1 M) was added to a mixture of **1** (32 mg, 0.2 mmol), the corresponding [LAuCl] complex (0.01 mmol, 5 mol%), and the corresponding activator (0.01 mmol, 5 mol%) at room temperature. An additive was added if it is specified. The resulting solution was stirred for 16 h at the specified temperature (sand bath). Then, the reaction was quenched with Et_3N (1.4 µL, 0.01 mmol) and the solvent was eliminated under reduced pressure. NMR yield was determined by ^{1}H NMR ($CDCl_3$) using 1,3,5-trimethoxybenzene as internal standard (see **Table S1**).

Spectroscopic data of products **2** and **2'** are in accordance with those reported in literature.[14] The following diagnostic signals were integrated to quantify the amount of starting material and products: for substrate **1**, peaks at 2.28 ppm (t, *J* = 2.6 Hz, 1H) and 4.26 ppm (dd, *J* = 5.2, 2.6 Hz, 2H); for product **2**, peaks at 4.39 ppm (q, *J* = 2.7 Hz, 1H) and 4.70 ppm (t, *J* = 2.9 Hz, 2H); for product **2'**, peak at 2.39 ppm (d, *J* = 1.3 Hz, 3H); for 1,3,5-trimethoxybenzene, peak at 6.09 ppm (s, 3H).

Table S1. Control experiments and optimization of the reaction parameters[a]

Entry	Solvent	LAuCl	Activator	Additive	T (°C)	Yield (%)[b]
**1**	CH_2Cl_2	**Au1**	**I**	--	25	58
**2**	CH_2Cl_2	**Au1**	**I**	H_2O (50 mol%)	25	>95
**3**	CH_2Cl_2	**Au1**	**--**	H_2O (50 mol%)	25	<5
**4**	CH_2Cl_2	**Au1**	**I**	TFA (10 mol%)	25	0 [**2'**: 90][c]
**5**	CH_2Cl_2	**Au1**	**--**	TFA (10 mol%)	25	7 [**2'**: 40][c]
**6**	CH_2Cl_2	**Au1**	**I**	$Zn(OTf)_2$ (5 mol%)	25	>95
**7**	CH_2Cl_2	**Au1**	**--**	$Zn(OTf)_2$ (5 mol%)	25	>95
**8**	CH_2Cl_2	**Au1**	**I**	MS 4Å	25	91
**9**	CH_2Cl_2	**Au1**	**--**	MS 4Å	25	21
**10**	CH_2Cl_2	**Au1**	**VI**	--	25	<5
**11**	CH_2Cl_2	**Au1**	**VII**	--	25	<5
**12**	CH_2Cl_2	**Au1**	**VIII**	--	25	<5
**13**	CH_2Cl_2	**Au1**	**I**	--	35	93
**14**	$CHCl_3$	**Au1**	**I**	--	35	>95
**15**	DCE	**Au1**	**I**	--	35	>95 (98)[d]
**16**	DCE	**Au1**	**I**	--	50	90 [**2'**: 10][c, e]
**17**	Toluene	**Au1**	**I**	--	35	46
**18**	TFT	**Au1**	**I**	--	35	90
**19**	Et_2O	**Au1**	**I**	--	35	<5
**20**	DME	**Au1**	**I**	--	35	<5
**21**	THF	**Au1**	**I**	--	35	<5
**22**	Acetone	**Au1**	**I**	--	35	<5
**23**	DCE	**Au2**	**I**	--	35	79
**24**	DCE	**Au3**	**I**	--	35	63
**25**	DCE	**Au4**	**I**	--	35	50
**26**	DCE	**Au5**	**I**	--	35	73
**27**	DCE	**Au7**	**I**	--	35	41
**28**	DCE	**Au1**	**II**	--	35	70
**29**	DCE	**Au1**	**III**	--	35	71 [**2'**: 13][c]
**30**	DCE	**Au1**	**IV**	--	35	21
**31**	DCE	**Au1**	**V**	--	35	60

[a] Reactions were performed at 0.2 mmol scale. Reaction time: 16 h. [b] Estimated by ^1H NMR employing 1,3,5-trimethoxybenzene as internal standard. [c] NMR yield of **2'**. [d] Isolated yield after column chromatography. [e] Reaction time: 10 h.

6.2. General procedure for cyclization of *N*-propargyl benzamide

In a flame-dried schlenk tube, freshly distilled anhydrous 1,2-DCE (2.0 mL, 0.1 M) was added to a mixture of **1** (32 mg, 0.2 mmol), **Au1** (5.2 mg, 0.01 mmol, 5 mol%) and **I** (6 mg, 0.01 mmol, 5 mol%) at room temperature. The resulting solution was stirred for 16 h at 35 ºC (sand bath). Then, the reaction was quenched with Et_3N (1.4 µL, 0.01 mmol) and the solvent was eliminated under reduced pressure. NMR yield was determined by ^1H NMR ($CDCl_3$) using 1,3,5-trimethoxybenzene as internal standard and the resulting residue was purified by column chromatography on silica gel (EtOAc/hexane 1/5) to afford **2** as yellow oil (31.2 mg, 0.196 mmol, 98%). **^{1}H NMR** (300 MHz, CD_2Cl_2): δ 7.99 – 7.95 (m, 2 H), 7.56 – 7.50 (m, 1 H), 7.48 – 7.42 (m, 2 H), 4.80 (q, *J* = 2.7 Hz, 1 H), 4.63 (t, *J* = 2.7 Hz, 2 H), 4.37 (q, *J* = 2.7 Hz, 1 H). The experimental data are in accordance with those reported in literature.[14]

7. Heterocyclization and subsequent 1,2-alkyl shift of α-hydroxy α-alkynyl ketone

7.1. Optimization of reaction conditions

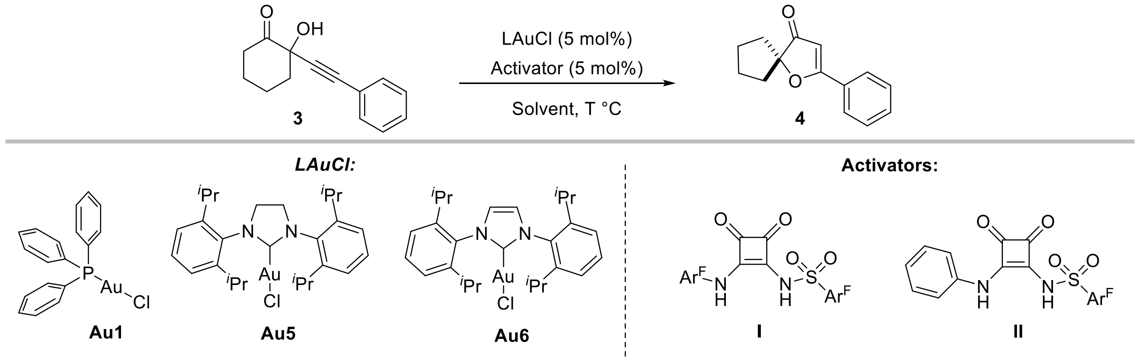

Scheme S2. Gold (I) chloride complexes and activators tested in heterocyclization and subsequent 1,2-alkyl shift of α-hydroxy α-alkynyl ketone **3**.

In a flame-dried schlenk tube, the corresponding freshly distilled anhydrous solvent (M) was added to a mixture of **3** (43 mg, 0.2 mmol), the corresponding [LAuCl] complex (0.01 mmol, 5 mol%), and the corresponding activator (0.01 mmol, 5 mol%) at room temperature. The resulting solution was stirred for 24 h at the specified temperature using sand bath. Then, the reaction was quenched with Et_3N (1.4 µL, 0.01 mmol) and the solvent was eliminated under reduced pressure. NMR yield was determined by ^1H NMR ($CDCl_3$) using 1,3,5-trimethoxybenzene as internal standard (see **Table S2**).

Spectroscopic data of product **4** are in accordance with those reported in literature.[15] The following diagnostic signals were integrated to quantify the amount of starting material and product: for substrate **3**, peak at 3.00 ppm (dt, J = 13.8, 6.2 Hz, 1H); for product **4**, peak at 6.00 ppm (s, 1H); for 1,3,5-trimethoxybenzene, peak at 6.09 ppm (s, 3H).

Table S2. Control experiments and optimization of the reaction parameters[a]

Entry	Solvent [M]	LAuCl	Activator	T (°C)	Yield (%)^b	
					4	3
**1**	CH_2Cl_2 [0.2]	**Au1**	--	40	<5	>95
**2**	CH_2Cl_2 [0.2]	**Au1**	$AgNTf_2$	40	66	<5
**3**	CH_2Cl_2 [0.2]	--	$AgNTf_2$	40	23	55
**4**	CH_2Cl_2 [0.2]	**Au1**	$NaBAr^F$	40	49	<5
**5**	CH_2Cl_2 [0.2]	**Au1**	$NaBF_4$	40	<5	>95
**6**	CH_2Cl_2 [0.2]	**Au1**	**I**	40	22	73
**7**	CH_2Cl_2 [0.2]	--	**I**	40	<5	>95
**8**	DCE [0.2]	**Au1**	**I**	50	37	52
**9**	DCE [0.2]	**Au1**	**I**	60	66	15
**10**	DCE [0.2]	**Au1**	**I**	70	81	6
**11**	DCE [0.03]	**Au1**	**I**	70	>95 (98)^c	<5
**12**^{d}	DCE [0.03]	**Au1**	**I**	70	>95	<5
**13**	Toluene [0.03]	**Au1**	**I**	70	26	32
**14**	DCE [0.03]	**Au6**	**I**	70	>95 (98)^c	<5
**15**	DCE [0.03]	**Au1**	**II**	70	11	89
**16**	DCE [0.03]	**Au6**	**II**	70	13	83
**17**^{e}	DCE [0.03]	**Au1**	**I**	70	>95 (93)^c	<5

^{a} Reactions were performed at 0.2 mmol scale. Reaction time: 24 h. ^{b} Estimated by ^{1}H NMR employing 1,3,5-trimethoxybenzene as internal standard. ^{c} Isolated yield after column chromatography. ^{d} **Au1** (2.5 mol %)/**I** (2.5 mol %). ^{e} Reaction was performed at 1 mmol scale.

7.2. General procedure for heterocyclization and subsequent 1,2-alkyl shift of α-hydroxy α -alkynyl ketone (1mmol scale)

In a flame-dried schlenk tube, freshly distilled anhydrous 1,2-DCE (33.5 mL, 0.03 M) was added to a mixture of **3** (214.1 mg, 1 mmol), **Au1** (26 mg, 0.05 mmol, 5 mol%) and **I** (30 mg, 0.05 mmol, 5 mol%) at room temperature. The resulting solution was stirred for 24 h at 70 ºC (sand bath). Then, the reaction was quenched with Et_3N (7 µL, 0.05 mmol) and the solvent was eliminated under reduced pressure. NMR yield was determined by ^{1}H NMR ($CDCl_3$) using 1,3,5-trimethoxybenzene as internal standard and the resulting residue was purified by column chromatography on silica gel (EtOAc/Hex 1/10) to afford **4** as a pale yellow solid (199.1 mg, 0.93 mmol, 93%). **^{1}H NMR** (300 MHz, $CDCl_3$): δ

7.83 – 7.79 (m, 2H), 7.56 – 7.43 (m, 3H), 6.00 (s, 1H), 2.09 – 1.88 (m, 8H). The experimental data are in accordance with those reported in literature.[15]

8. General procedure for tandem cycloisomerization and nucleophilic addition

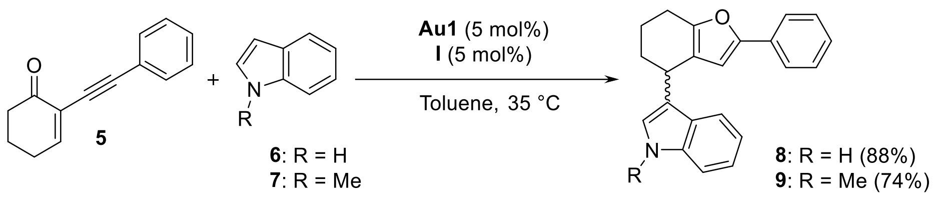

General procedure: In a flame-dried schlenk flask, freshly distilled anhydrous toluene (1.0 mL, 0.2 M) was added to a mixture of **5** (43.1 mg, 0.22 mmol), the corresponding nucleophile **6-7** (0.2 mmol), **Au1** (5.2 mg, 0.01 mmol, 5 mol%), and **I** (6 mg, 0.01 mmol, 5 mol%) at room temperature. The resulting solution was stirred for 16 h at the same temperature. Then, the reaction was quenched with Et_3N (1.4 µL, 0.01 mmol) and the solvent was eliminated under reduced pressure. The resulting residue was purified by column chromatography on silica gel (Toluene/Hexane 4/1).

3-(2-Phenyl-4,5,6,7-tetrahydrobenzofuran-4-yl)-1*H*-indole (8)

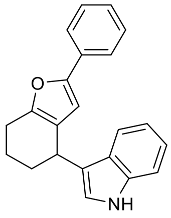

Following the general procedure, using **6** as nucleophile (23.4 mg, 0.20 mmol), compound **8** was obtained as a pale orange solid (55 mg, 0.18 mmol, 88%). **^{1}H NMR (300 MHz, $CDCl_3$)**: δ 7.88 (s, 1H), 7.68 – 7.61 (m, 3H), 7.39 – 7.33 (m, 3H), 7.27 – 7.13 (m, 3H), 6.87 (d, *J* = 2.2 Hz, 1H), 6.48 (s, 1H), 4.29 (t, *J* = 5.7 Hz, 1H), 2.81 (t, *J* = 6.1 Hz, 2H), 2.25 – 2.16 (m, 1H), 2.05 – 1.94 (m, 2H), 1.93 – 1.82 (m, 1H). The experimental data are in accordance with those reported in literature.[16]

1-Methyl-3-(2-phenyl-4,5,6,7-tetrahydrobenzofuran-4-yl)-1*H*-indole (9)

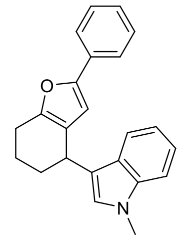

Following the general procedure, using **7** as nucleophile (26.4 µL, 0.20 mmol), compound **9** was obtained as a pale yellow oil (49 mg, 0.16 mmol, 74%). **^{1}H NMR (300 MHz, $CDCl_3$)**: δ 7.63 – 7.58 (m, 3H), 7.35 – 7.30 (m, 3H), 7.24 – 7.16 (m, 2H), 7.14 – 7.08 (m, 1H), 6.73 (s, 1H), 6.45 (s, 1H), 4.25 (t, J = 4.9 Hz, 1H), 3.73 (s, 3H), 2.78 – 2.74 (m, 2H), 2.22 – 2.11 (m, 1H), 2.05 – 1.92 (m, 2H), 1.91 – 1.78 (m, 1H). The experimental data are in accordance with those reported in literature.[16]

9. General procedure for intermolecular olefin cyclopropanation

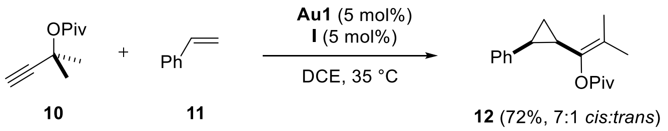

In a flame-dried schlenk tube, freshly distilled anhydrous 1,2-DCE (4 mL, 0.05 M) was added to a mixture **Au1** (5.2 mg, 0.01 mmol, 5 mol%) and **I** (6 mg, 0.01 mmol, 5 mol%). Then, **11** (93 µL, 0.08 mmol) was added followed by **10** (40 µL, 0.2 mmol) at room temperature. The resulting solution was stirred for 18 h at 35 ºC (sand bath). Then, the reaction was quenched with Et_3N (1.4 µL, 0.02 mmol) and the solvent was eliminated under reduced pressure. The resulting residue was purified by column chromatography on silica gel (Hexane→EtOAc/Hexane 1:70→1:50) to afford **12** as a white oil (39.2 mg, 0.14 mmol, 72%, 7:1 inseparable mixture of diastereomers). **^{1}H NMR** (300 MHz, $CDCl_3$): *Signals corresponding to the major diastereoisomer*: δ 7.26 – 7.21 (t, 2 H), 7.16 – 7.06 (m, 1 H), 7.05 – 7.02 (m, 2 H), 2.27 (t, 2 H, J = 6.5 Hz), 1.49 (s, 3 H), 1.41 (s, 3 H), 1.29 (br s, 1 H), 1.22 (s, 9 H), 1.00 (q, J = 6.5 Hz, 1 H). The experimental data are in accordance with those reported in literature.[17]

10. Computational studies

10.1. Computational methods

All of the calculations were performed using the Gaussian16 program.[18] Computations were done using wb97xd functional[19] in conjunction with standard basis sets def2SVP and def2TZVP.[20] Geometry full optimizations were made at wb97xd/def2SVP level. Single point calculations using def2TZVP basis set were carried out over optimized geometries to obtain the energy values. Solvent effects (toluene) were considered using the SMD model.[21] The nature of stationary points was defined on the basis of calculations of normal vibrational frequencies (force constant Hessian matrix). The optimizations were carried out using the Berny analytical gradient optimization method.[22] Minimum energy pathways for the reactions studied were found by gradient descent of transition states in the forward and backward direction of the transition vector (IRC analysis).[23] Analytical second derivatives of the energy were calculated to classify the nature of every stationary point, to determine the harmonic vibrational frequencies, and to provide zero-point vibrational energy corrections. The thermal and entropic contributions to the free energies were also obtained from the vibrational frequency calculations, using the unscaled frequencies. Correction to free energy was made by substracting S_{trans} contribution and considering a 1 M concentration.[24] Structural representations were generated using CYLView.[25]

ELF analysis

The electronic structures of stationary points were analyzed by the topological analysis of the gradient field of electron localization function (ELF)[26] developed by Silvi and Savin.[27] The ELF study was performed with the TopMod program[28] using the corresponding wavefunctions of the all structures of the IRC. The topological analysis of the gradient field of ELF has showed to be a powerful tool for the study of the bonding changes along an organic reaction.[29]

NCI Calculations

NCI (non-covalent interactions) were computed using the methodology previously described.[30] Quantitative data were obtained with the NCIPLOT4 program.[31] A density cutoff of ρ=0.5 a.u. was applied and isosurfaces of s(r) =0.5 were colored by $sign(\lambda_2)\rho$ in the [-0.03,0.03] a.u. range using VMD software.[32] $s(\mathbf{r})$ against $sign(\lambda_2)\rho(\mathbf{r})$ plots were generated with gnuplot software.[33]

Molecular Dynamics

MD simulations were carried out with AMBER20 suite of programs.[34] Parameters for trisaccharides were generated with the antechamber module using the general Amber force field (GAFF2),[35] with partial charges calculated using AM1-BCC method. The system to be studied was neutralized if necessary, and immersed in a chloroform box of 12 Å. A two-stage geometry optimization approach was carried out: (i) minimization of only the positions of solvent molecules executed by 500 cycles of steepest descent minimization followed by 500 cycles of conjugate gradient minimization and (ii) unrestrained minimization of all the atoms in the simulation cell executed by 2500 cycles of steepest descent minimization followed by 2500 cycles of conjugate gradient minimization. After system optimization, running of MD simulations was started on the systems by gradually heating each system in the NVT ensemble from 0 to 300 K for 100 ps using a Langevin thermostat with a coupling coefficient of 1.0/ps. Harmonic restraints of 10 kcal·mol-1 were applied to the solute, and the Langevin temperature coupling scheme[36] was used to control and equalize the temperature. The time step was kept at 2 fs during the heating stages, allowing potential inhomogeneities to self-adjust. Water molecules are treated with the SHAKE algorithm such that the angle between the hydrogen atoms is kept fixed. Long-range electrostatic effects are modelled using the particle-mesh-Ewald method.[37] Then 5 ns of density equilibration with a force constant of 2.0 kcal/mol·Å^2 was performed by releasing all the restraints. Finally, production trajectories were then run for 100 ns under the same simulation conditions with an integration time step of 0.5 fs, recording geometry every 0.05 ps and with snapshots written each 2 ps, producing 50,000 frames per simulation. All MD simulations were replicated three times to ensure feasibility.

10.2. MD simulations

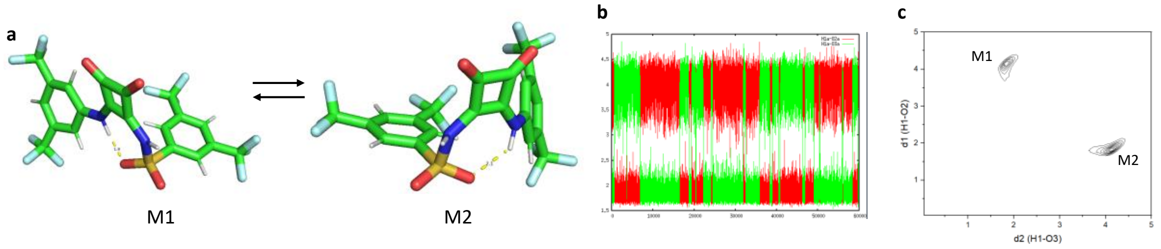

Figure S1. MD simulations of the monomer of squaramide. a) Two equivalent structures are only present showing identical H-bonds between one amino group and the sulfone group. b) Monitoring the two equivalents NH···OSOH H-bonds during 300 ns (step: 5 ps). c) Contour graph showing the two representative identical conformations for the monomer of squaramide.

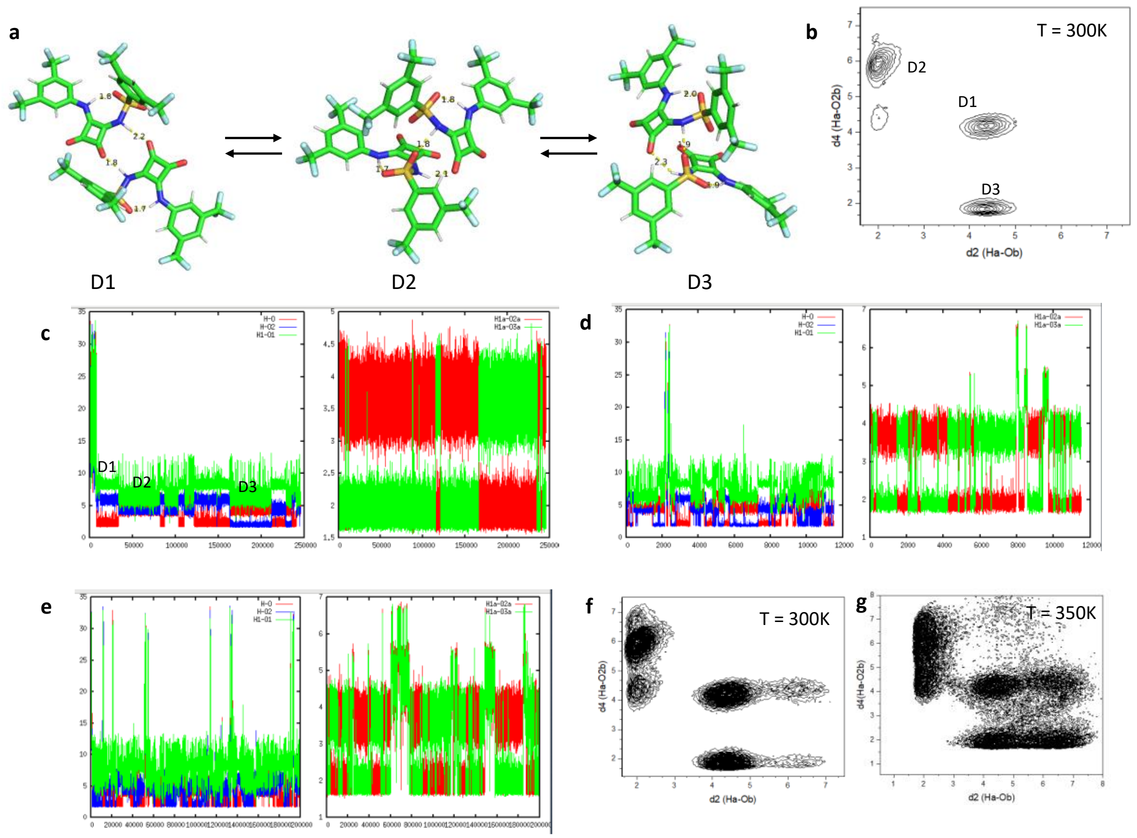

Figure S2. MD simulations of the dimer of squaramide. a) Three species, two of them (D2 and D3) equivalent showing identical H-bonds are detected. b) Contour plot indicating the relative populations of each dimer. c-e) Monitoring the NH···OSOH H-bond (step: 5 ps) at 300 K (c), 325 K (d) and 350 K (e). Note that at 300K the three species alternate in time indicating a high degree of association. However, at 350 K the alternance is broken and no defined areas for each species can be observed, indicating a high degree of disaggregation. At 325 K there is an intermediate situation. f-g) Analysis of more than 250.000 snapshots (limit of detection: 100 for groups within 0.05 Å, i.e: 0.04%) at 300 K (f) and 350 K (g). The area with high values of bond distances (up right) corresponds to completely disaggregates units.

10.3. Catalytic cycles

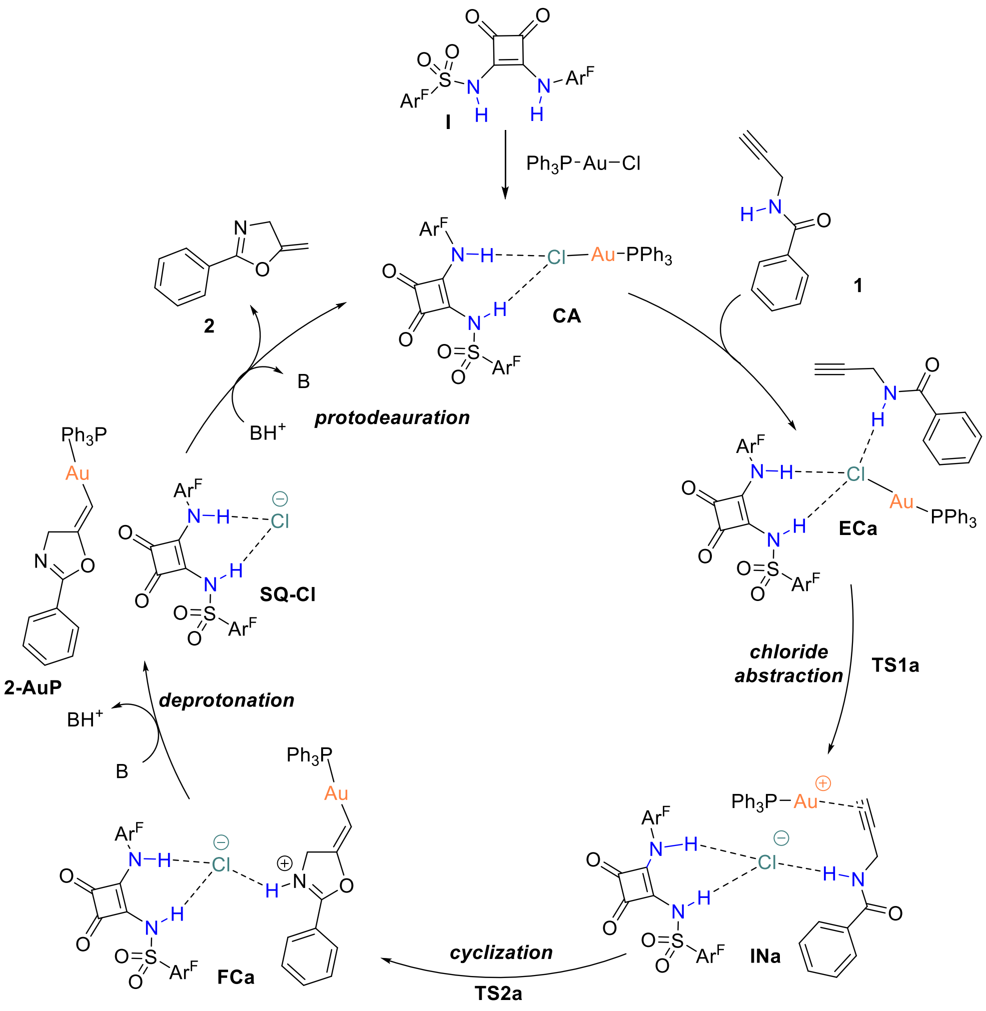

Scheme S3. Catalytic cycle for the cyclization of *N*-propargyl benzamide **1** to oxazoline **2**.

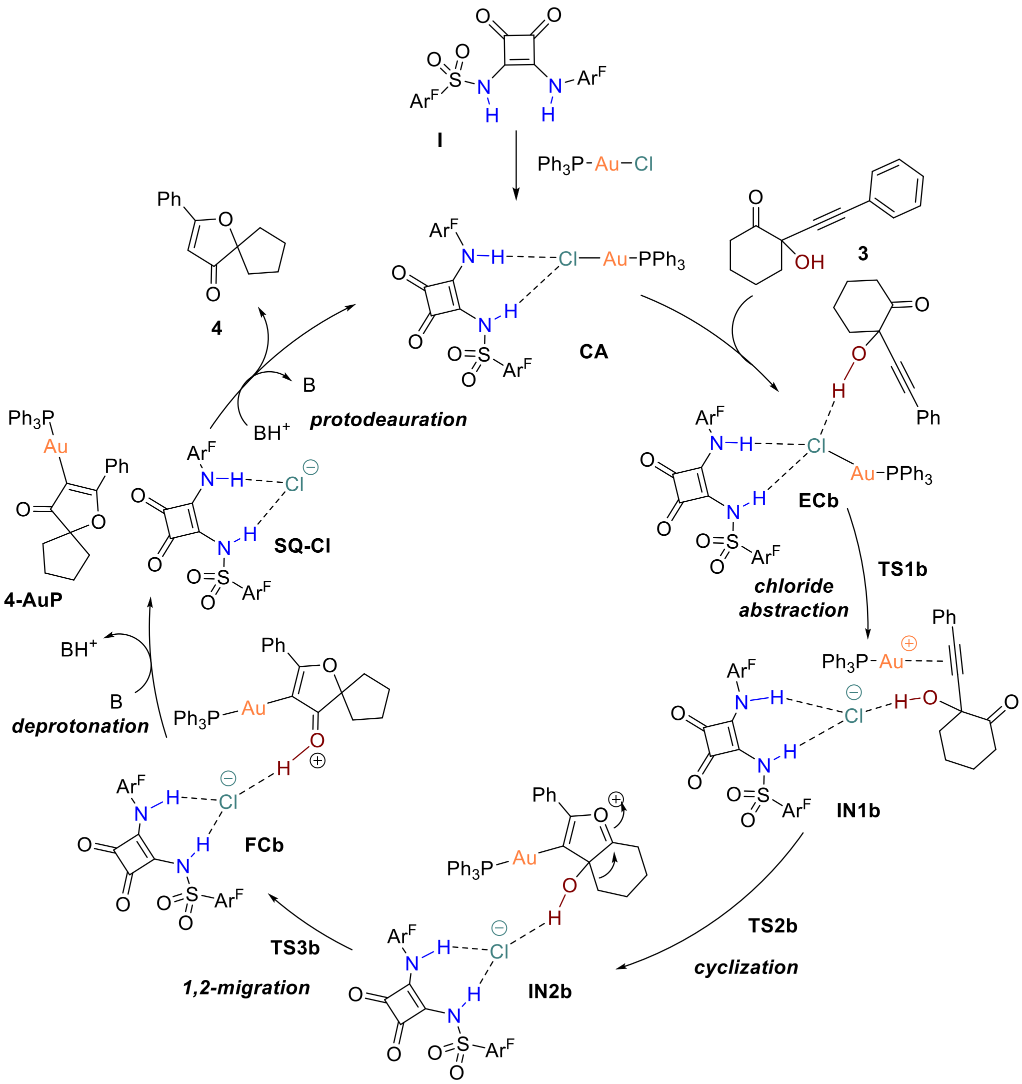

Scheme S4. Catalytic cycle for the heterocyclization/1,2-migration cascade.

In this reaction, a simultaneous migration of a bond seems to be necessary to take place when the cyclization is produced. However, any attempt of locating a single transition structure in which both cyclization and migration takes place at the same time, failed. On the other hand, the reaction proceeds in a similar way to the formation of oxazolines, that is forming a reactive intermediate (**IN1b**) that cyclizes (through **TS2b**). A subsequent 1,2-migration through a third transition structure **TS3b** would yield the product. The energy barrier for the last migration step (18.7 kcal/mol) is the rate limiting stage of the process. Consequently, we have, in this case, a two-step reaction, being the second step the migration to form the spiro derivative. Finally, the catalytic cycle would continue as usual with a deprotonation and protodeauration, yielding the spirocycle **4** (Scheme S4). Similarly, to the model reaction showed in Scheme S3, the Au-Cl bond is not completely broken in the first step and only

after the second transition structure can be considered that the Au atom is forming a bond with the triple bond (now becoming a double bond).

10.4. Optimized geometries

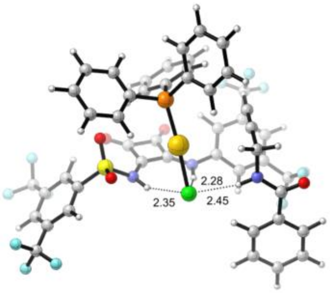

ECa

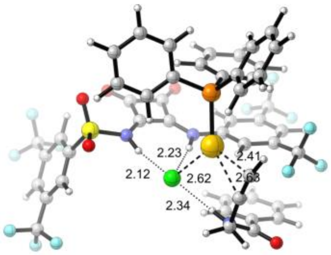

TS1a

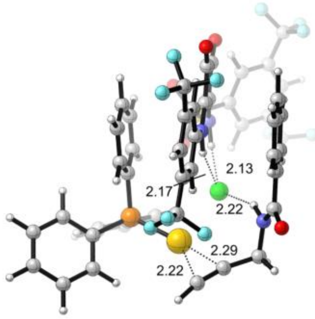

INa

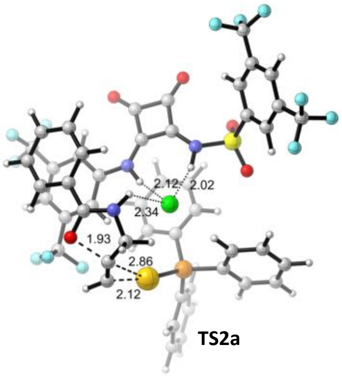

TS2a

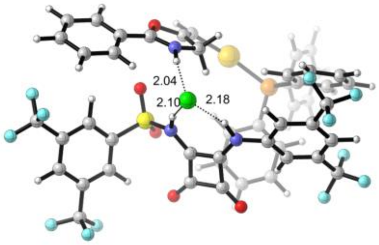

FCa

Figure S3. Optimized structures (b3lyp-gd3bj/def2svp/pcm=DCM) of the stationary points corresponding to the reaction of compound **1**.

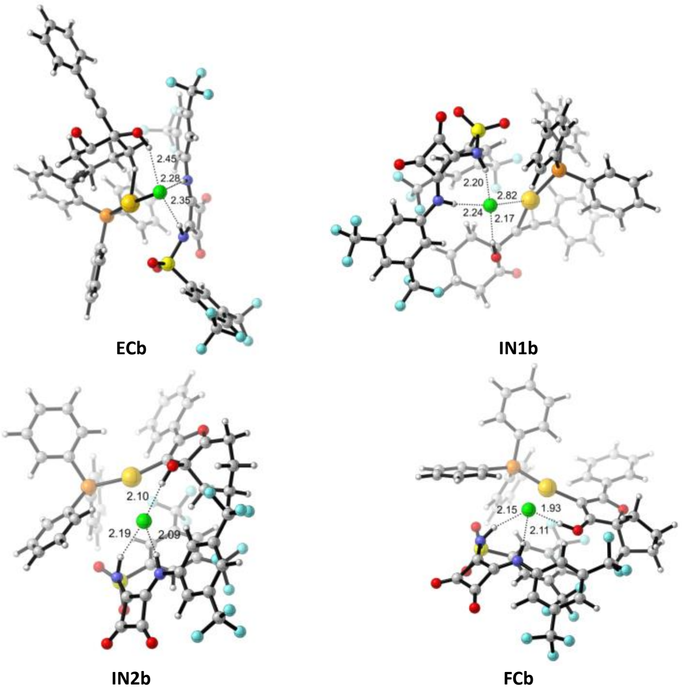

Figure S4. Optimized structures (b3lyp-gd3bj/def2svp/pcm=DCM) of the minima corresponding to the reaction of compound **3**.

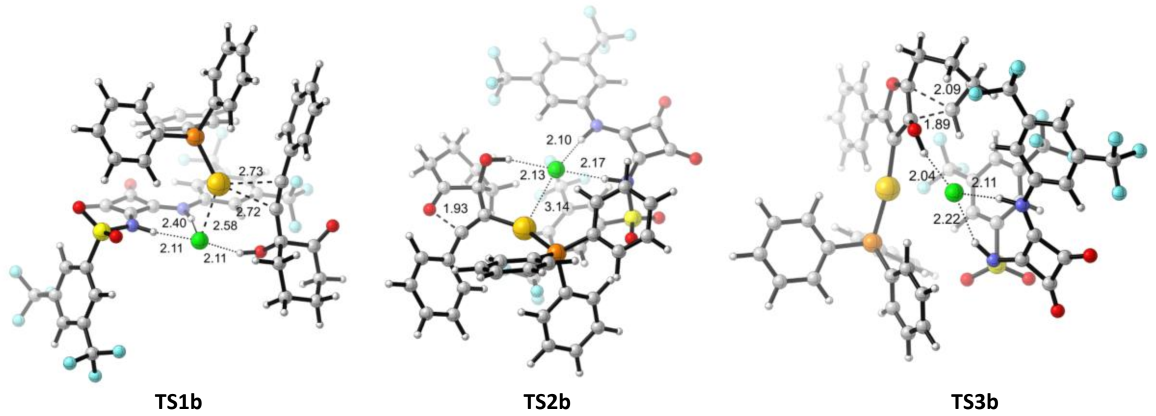

Figure S5. Optimized structures (b3lyp-gd3bj/def2svp/pcm=DCM) of the transition structures corresponding to the reaction of compound **3**.

10.5. Topological analyses

The electron localization function (ELF) was introduced by Becke and Edgecombe[38] as a "simple measure of electron localization in atomic and molecular systems". The ELF analysis show the presence of electron density contributing to bonding.[26][27a] The NCI analysis provides information of non-covalent interactions showing non-bonding electron density involved in such interactions.[30a][31]

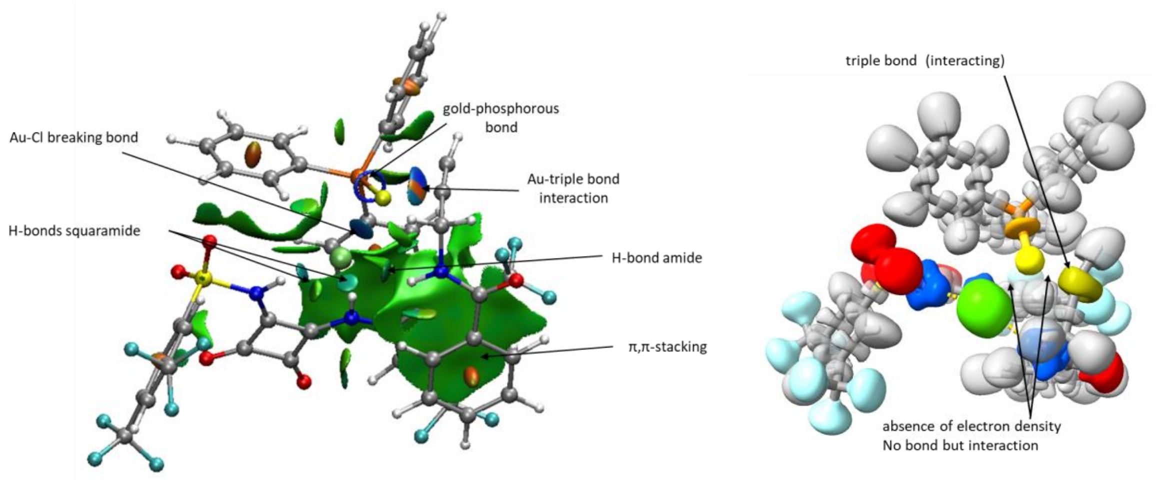

Figure S6. Left: Non-covalent interactions (NCI analysis) in **TS1a** showing the main interactions not involved in chemical bonds. It can be seen how the Au-Cl bond is already broken, although it remains an interaction between both atoms, and the metal atom has an interaction with the triple bond. Thin, delocalized green surface indicates van der Waals interactions. Small, lenticular, bluish surfaces indicate strong interactions such as hydrogen bonding. Steric clashes are shown as red isosurfaces. Right: Electron localization function (ELF) analysis showing electron density involved in bond (complementary to NCI). Note the absence of electron density between Au and Cl atoms and how the Au atom interacts with the triple bond which has not altered its typical toroid electronic structure.

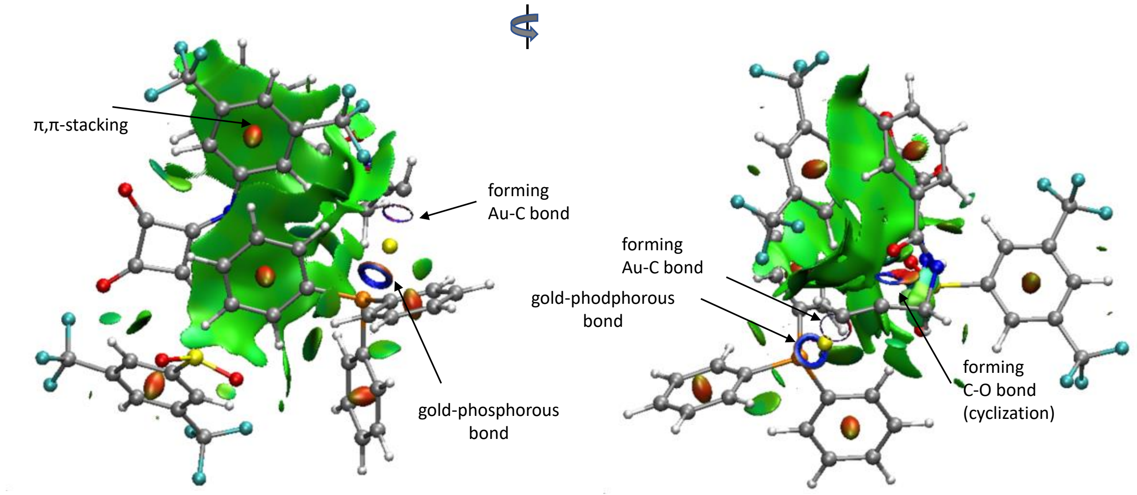

Figure S7. Non-covalent interactions (NCI analysis) in **TS2a** showing, in two views, the main interactions not involved in chemical bonds. It can be seen the incipient forming bond leading to the final cycle. Thin, delocalized green surface indicates van der Waals interactions. Small, lenticular, bluish surfaces indicate strong interactions such as hydrogen bonding. Steric clashes are shown as red isosurfaces.

10.6. Energies

Table S3. Absolute (hartree) and relative (kcal/mol) energies (b3lyp-gd3bj/def2tzvp/smd=DCM// b3lyp-gd3bj/def2svp/smd=DCM) for the transformation of **1** into **2**.

	E_0	ΔE_0	G	ΔG	im. freq
**1**	-516.482803		-516.521037		
**Au1**	-1632.540285		-1632.594534		
**I**	-2774.502753		-2774.571533		
**Reagents**	-4923.525842	0.0	-4923.687105	0.0	
**SQ-AuPCl**	-4923.560699	-21.9	-4923.700717	-8.5	
**EC1a**	-4923.578647	-33.1	-4923.693568	-4.1	
**TS1a**	-4923.574547	-30.6	-4923.682255	3.0	-48.5
**IN1a**	-4923.577466	-32.4	-4923.689086	-1.2	
**TS2a**	-4923.553410	-17.3	-4923.670446	10.5	-287.3
**FCa**	-4923.578336	-32.9	-4923.691213	-2.6	
**Product**	-4923.547698	-13.7	-4923.706340	-12.1	

Table S4. Absolute (hartree) and relative (kcal/mol) energies (b3lyp-gd3bj/def2tzvp/smd=DCM// b3lyp-gd3bj/def2svp/smd=DCM) for the transformation of **3** into **4**.

	E_0	ΔE_0	G	ΔG	im. freq
**3**	-692.390399		-692.434244		
**Au1**	-1632.540285		-1632.594534		
**I**	-2774.502753		-2774.571533		
**Reagents**	-5099.433438	0.0	-5099.600312	0.0	
**SQ-AuPCl**	-5099.468295	-21.9	-5099.613924	-8.5	
**EC1b**	-5099.491449	-36.4	-5099.607775	-4.7	
**TS1b**	-5099.480705	-29.7	-5099.600090	0.1	-46.4
**IN1b**	-5099.459542	-16.4	-5099.602788	-1.6	
**TS2b**	-5099.455049	-13.6	-5099.574341	16.3	-217.6
**IN2b**	-5099.459582	-16.4	-5099.577827	14.1	
**TS3b**	-5099.452602	-12.0	-5099.570549	18.7	-352.7
**FCb**	-5099.513712	-50.4	-5099.631842	-19.8	
**Product**	-5099.491710	-36.6	-5099.656070	-35.0	

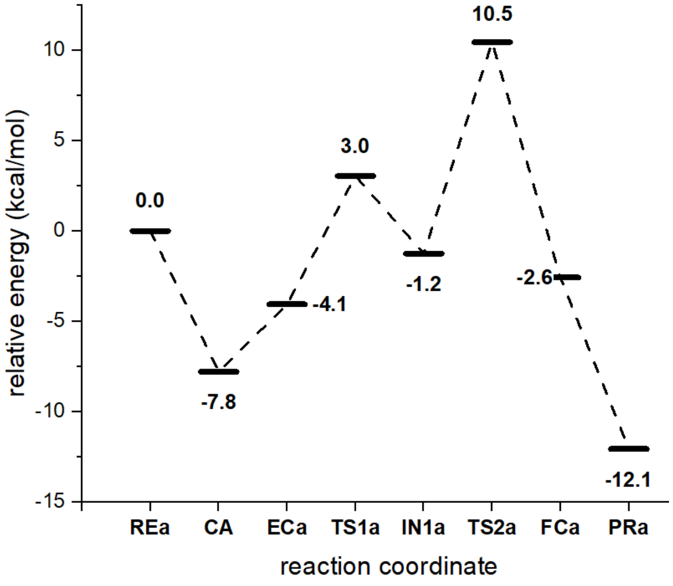

Figure S8. Energy profile (in kcal/mol) for the reaction of **1** to give **2**.

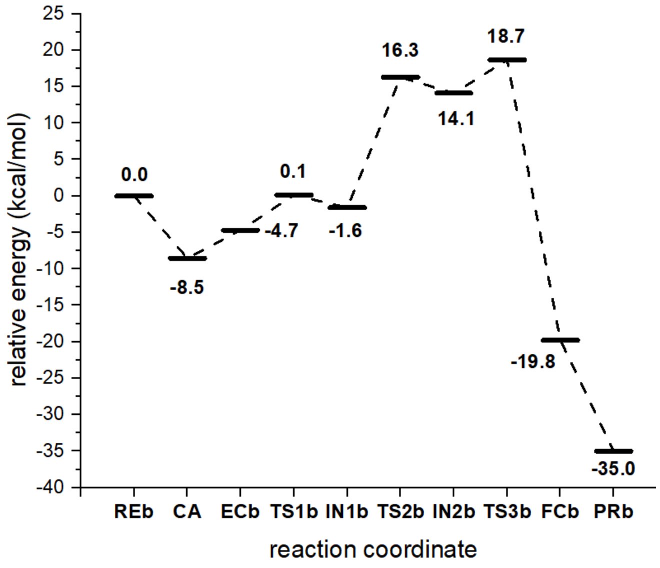

Figure S9. Energy profile (in kcal/mol) for the reaction of **3** to give **4**.

10.7. Cartesian Coordinates

CA

1 1

N	-0.9541154295	-0.4052829798	1.5077651111
H	-0.5926119426	-1.3812148910	1.4681201063
O	-1.3801076044	2.8334252807	1.0984287731
F	7.0605275312	2.5690102113	0.1131840122
N	2.0407445413	-0.2353851220	0.4904601518
H	1.4288413318	-1.9205777303	0.7448048463
O	1.7484323741	3.1373997776	0.2319538226
F	6.6608418765	2.3662998307	-2.0015319057
F	5.2736450268	3.4518946225	-0.7371643063
F	5.3631381591	-3.6969509648	-0.4536473301
F	6.7534595146	-2.6828160045	-1.7735805476
F	7.1165463164	-2.7149388935	0.3567099751
C	-0.2117735002	0.5814465458	1.1505048595
C	-0.4468143693	2.1073614066	0.9934236314
C	1.0428609774	2.2275150072	0.5468149898
C	1.1710411175	0.7122045634	0.6845934725
C	3.3420882064	-0.1169202100	0.1108382200
C	4.0124298153	1.1166334624	-0.1399937353
H	3.4880091916	2.0661399840	-0.0408968692
C	5.3404217312	1.0983779872	-0.5249906045
C	6.0304668671	-0.1177204426	-0.6710098729
H	7.0771436260	-0.1153534748	-0.9828335482
C	5.3796666414	-1.3348697518	-0.4227529757
C	4.0511687054	-1.3436721658	-0.0373033953
H	3.5385777253	-2.2853588973	0.1536509361
C	6.0857679485	2.3909090340	-0.7905551391
C	6.1561830054	-2.6265960963	-0.5748165318
Cl	0.7662439732	-3.0401439358	1.0215455338
S	-2.5920361941	-0.2753175520	2.3534930530
O	-2.6746187179	-1.5816319712	2.9753310475

O	-2.5194736834	0.9787681723	3.0747947170
C	-3.6972490433	-0.1574089758	0.9780260150
C	-4.0709032173	1.1106304607	0.5255035371
C	-4.1791947322	-1.3398691027	0.4131657816
C	-4.9511987637	1.1827754440	-0.5521455050
H	-3.6814281247	2.0120708470	0.9973298971
C	-5.0669828684	-1.2369185388	-0.6576315649
H	-3.8766653692	-2.3117022487	0.8029295852
C	-5.4494141213	0.0166600588	-1.1428977953
H	-6.1409161959	0.0834333902	-1.9841553554
C	-5.3609129067	2.5264887212	-1.1143420571
C	-5.6641408445	-2.4880522501	-1.2643719166
F	-4.8671735257	-3.5505432414	-1.0821669515
F	-6.8511653996	-2.7730020011	-0.7063094840
F	-5.8651053430	-2.3408977861	-2.5816147108
F	-5.0171766028	3.5311580133	-0.2986267727
F	-4.7722269139	2.7460716188	-2.3019806962
F	-6.6859097899	2.5850867884	-1.3105875037

Compound **1**

0 1

C	0.0744099844	3.9696205886	-13.8976420672
C	0.7740953670	4.4200097827	-14.7783735578
H	1.3989202699	4.8143150173	-15.5592311511
C	-0.7867717485	3.4417572123	-12.8333030686
H	-1.0009840703	4.2425285064	-12.1043802002
H	-0.2697490609	2.6374195829	-12.2888100919
N	-2.0238785317	2.8715808464	-13.3340709671
C	-2.3263589296	1.5507156867	-13.1853017641
H	-2.6790308108	3.4880574466	-13.7981289190
O	-1.6094990440	0.7774312216	-12.5573631201
C	-3.6102413864	1.0941588632	-13.8193259844
C	-4.2124783824	1.7581050956	-14.8996386935
C	-4.2020139940	-0.0747813319	-13.3169667939

C	-5.3975157415	1.2694697793	-15.4547541105
H	-3.7488503195	2.6449044271	-15.3374622974
C	-5.3899531582	-0.5575825763	-13.8657214805
H	-3.7121833622	-0.5915956106	-12.4903696189
C	-5.9911768456	0.1150198817	-14.9354717305
H	-5.8539883404	1.7884153392	-16.3005584414
H	-5.8483469538	-1.4629200138	-13.4612225302
H	-6.9194244515	-0.2644581852	-15.3690364216

Compound **2**

0 1

C	4.1131152804	6.4406339614	8.5697276913
C	4.9268536009	7.4953477511	8.5926894332
H	5.9843923949	7.3980746374	8.3417189931
C	2.6312658392	6.2913797540	8.8679907624
H	2.0062041950	6.9131392214	8.2038037299
H	2.3814460864	6.5860620271	9.9020005333
N	2.3607499662	4.8715902073	8.6547302388
C	3.4650132958	4.3353670474	8.3087048264
O	4.5569974701	5.1717462372	8.2285048822
C	3.7065315355	2.9237674667	7.9849556340
C	2.6391831948	2.0109409875	8.0445996405
C	4.9860813092	2.4767548686	7.6178477226
C	2.8521358776	0.6680819904	7.7400694050
H	1.6511767238	2.3739567800	8.3315623086
C	5.1931805829	1.1297190757	7.3138226724
H	5.8114901251	3.1876916758	7.5728136269
C	4.1293554770	0.2246057734	7.3740742147
H	2.0203409995	-0.0383234299	7.7873293972
H	6.1897268633	0.7854421157	7.0285684425
H	4.2940065538	-0.8288471122	7.1356733269
H	4.5357472089	8.4754747739	8.8676309281

Compound **3**

0 1

C	0	0.1412090000	-0.2545130000	-0.1461410000
C	0	-1.0693340000	-0.1779730000	-0.0715880000
H	0	1.6417920000	-2.1928760000	-0.9891450000
O	0	1.9820210000	-1.3192970000	-1.2335170000
C	0	1.5990740000	-0.4008900000	-0.2107910000
C	0	2.1771320000	-0.8343580000	1.1640760000
C	0	3.7950080000	0.8408490000	-0.6721130000
C	0	3.7009510000	-0.9649540000	1.1149910000
H	0	1.8756260000	-0.0873510000	1.9172410000
H	0	1.7004530000	-1.7860930000	1.4516330000
C	0	4.3604180000	0.3437220000	0.6743680000
H	0	4.1953140000	1.8266930000	-0.9465450000
H	0	4.0486220000	0.1173630000	-1.4634710000
H	0	4.0747530000	-1.2641620000	2.1068690000
H	0	3.9710210000	-1.7700400000	0.4132050000
H	0	5.4512030000	0.2202700000	0.5892740000
H	0	4.1872670000	1.1186130000	1.4411840000
C	0	2.2897540000	0.9371690000	-0.6014760000
O	0	1.6662490000	1.9592150000	-0.7694110000
C	0	-2.4939460000	-0.0706970000	0.0116930000
C	0	-3.3207170000	-1.1234230000	-0.4321710000
C	0	-3.0917550000	1.0929350000	0.5388370000
C	0	-4.7084680000	-1.0111630000	-0.3493180000
H	0	-2.8632530000	-2.0258810000	-0.8420280000
C	0	-4.4803150000	1.1957590000	0.6189830000
H	0	-2.4557140000	1.9111290000	0.8816130000
C	0	-5.2927700000	0.1463870000	0.1760010000
H	0	-5.3386790000	-1.8328770000	-0.6973540000
H	0	-4.9319950000	2.1019480000	1.0292230000
H	0	-6.3800320000	0.2308260000	0.2396120000

Compound **4**

0 1

C	0	-1.3198590000	4.2702450000	0.9495600000
C	0	-1.6551210000	5.7387410000	0.6722990000
C	0	-1.9228250000	5.7749870000	-0.8448670000
C	0	-1.0762550000	4.6245500000	-1.4505060000
H	0	-0.7843940000	6.3638920000	0.9246410000
H	0	-2.5065460000	6.0952990000	1.2699270000
H	0	-2.9873470000	5.5869100000	-1.0433410000
H	0	-1.6715440000	6.7493610000	-1.2870260000
H	0	-1.7033690000	3.9134710000	-2.0037400000
H	0	-0.2815090000	4.9680920000	-2.1262550000
C	0	-0.4381530000	3.8906530000	-0.2479330000
O	0	0.8973510000	4.4061120000	-0.0150720000
C	0	1.7658210000	3.3969010000	-0.0892000000
C	0	1.1876390000	2.1414180000	-0.3683410000
Au	0	2.1771940000	0.3415510000	-0.6490600000
P	0	3.7354300000	-1.4048580000	-0.8276110000
C	0	3.2044260000	-3.0984250000	-1.2258530000
C	0	2.0471450000	-3.2616540000	-2.0000970000
C	0	3.9093940000	-4.2262060000	-0.7769500000
C	0	1.5927130000	-4.5433720000	-2.3144330000
H	0	1.4794240000	-2.3886220000	-2.3259140000
C	0	3.4506550000	-5.5055860000	-1.0954510000
H	0	4.8048530000	-4.1097360000	-0.1639580000
C	0	2.2909070000	-5.6657860000	-1.8608090000
H	0	0.6800600000	-4.6632590000	-2.9023290000
H	0	3.9970100000	-6.3812310000	-0.7377070000
H	0	1.9279660000	-6.6687090000	-2.0974650000
C	0	5.0508550000	-0.9729420000	-2.0284660000
C	0	5.8925820000	-1.9536940000	-2.5755420000
C	0	5.2458620000	0.3751600000	-2.3711880000
C	0	6.9195580000	-1.5873970000	-3.4481160000
H	0	5.7447080000	-3.0057690000	-2.3265630000

C	0	6.2794000000	0.7381900000	-3.2383730000
H	0	4.5892090000	1.1425870000	-1.9549730000
C	0	7.1167740000	-0.2422490000	-3.7782170000
H	0	7.5681070000	-2.3568500000	-3.8730110000
H	0	6.4253660000	1.7896680000	-3.4954860000
H	0	7.9211290000	0.0405620000	-4.4611830000
C	0	4.5981920000	-1.4877620000	0.7882980000
C	0	4.0471310000	-2.2400980000	1.8392630000
C	0	5.7151250000	-0.6737830000	1.0306610000
C	0	4.6064880000	-2.1672470000	3.1169170000
H	0	3.1814100000	-2.8796400000	1.6599920000
C	0	6.2664990000	-0.6029040000	2.3127110000
H	0	6.1547260000	-0.0902290000	0.2199600000
C	0	5.7115630000	-1.3451420000	3.3591310000
H	0	4.1716240000	-2.7551390000	3.9286100000
H	0	7.1350730000	0.0350500000	2.4924110000
H	0	6.1421260000	-1.2861740000	4.3612790000
C	0	3.1636660000	3.7684060000	0.1214860000
C	0	4.1272650000	2.8216290000	0.5214590000
C	0	3.5555730000	5.1099930000	-0.0672380000
C	0	5.4525640000	3.2061820000	0.7098080000
H	0	3.8304930000	1.7907550000	0.7076580000
C	0	4.8849890000	5.4854700000	0.1115090000
H	0	2.8108470000	5.8465250000	-0.3698420000
C	0	5.8367560000	4.5347650000	0.4982040000
H	0	6.1871600000	2.4652550000	1.0307070000
H	0	5.1821180000	6.5235370000	-0.0511040000
H	0	6.8782930000	4.8316670000	0.6407690000
C	0	-0.1733720000	2.4312610000	-0.4536400000
O	0	-1.1826370000	1.6502860000	-0.6792980000
H	0	-0.9015980000	0.7131990000	-0.9471430000
Cl	0	-0.6531070000	-0.9658570000	-1.8590290000
H	0	-0.7676400000	-2.7937220000	-0.7403890000
H	0	-2.6385540000	-1.4191590000	-1.3195550000
N	0	-1.0852300000	-3.5369990000	-0.0860530000

N	0	-3.5645630000	-1.7847590000	-1.0352930000
C	0	-2.4465600000	-3.7833840000	-0.0741790000
S	0	-0.2584970000	-3.4236040000	1.3925560000
C	0	-3.5381780000	-3.0072500000	-0.4877270000
C	0	-4.6124800000	-0.8632910000	-1.1289640000
C	0	-3.2848770000	-4.9130520000	0.3930070000
O	0	-0.9746270000	-4.2661230000	2.3457220000
O	0	1.1622790000	-3.6138880000	1.1185340000
C	0	-0.4938470000	-1.7032630000	1.8682800000
C	0	-4.5166200000	-4.0542020000	-0.0018260000
C	0	-5.9404040000	-1.1638010000	-0.7958420000
C	0	-4.2741120000	0.4365300000	-1.5367190000
O	0	-3.1230760000	-6.0080380000	0.8688450000
C	0	0.6091480000	-0.9337790000	2.2254280000
C	0	-1.7916300000	-1.1896100000	1.9178130000
O	0	-5.7146670000	-4.1623330000	0.0681980000
H	0	-6.2202290000	-2.1743280000	-0.4962170000
C	0	-6.9027060000	-0.1541760000	-0.8532080000
C	0	-5.2481520000	1.4304920000	-1.5709640000
H	0	-3.2440900000	0.6703490000	-1.8033580000
C	0	0.3984400000	0.3819220000	2.6536080000
H	0	1.6133000000	-1.3507940000	2.1809700000
C	0	-1.9791890000	0.1362210000	2.2969310000
H	0	-2.6515660000	-1.8083180000	1.6728160000
C	0	-6.5737880000	1.1494980000	-1.2323870000
C	0	-8.3159920000	-0.4723030000	-0.4341540000
C	0	-4.8377930000	2.8402870000	-1.9054490000
C	0	-0.8866700000	0.9232480000	2.6789170000
C	0	1.5558600000	1.2035710000	3.1704960000
C	0	-3.3655070000	0.7360080000	2.2970110000
H	0	-7.3352730000	1.9271720000	-1.2701870000
F	0	-8.4786930000	-0.3253850000	0.8960180000
F	0	-9.2104250000	0.3366640000	-1.0283470000
F	0	-8.6555090000	-1.7380300000	-0.7308290000
F	0	-5.8491290000	3.5581520000	-2.4162130000

F	0	-4.4068190000	3.5012810000	-0.8058610000
F	0	-3.8256920000	2.8749240000	-2.7931660000
H	0	-1.0424330000	1.9513790000	3.0058990000
F	0	2.7379520000	0.7548330000	2.7211700000
F	0	1.4487470000	2.4993440000	2.8174570000
F	0	1.6050820000	1.1716280000	4.5138280000
F	0	-4.3154270000	-0.1947890000	2.1166660000
F	0	-3.6254290000	1.3726770000	3.4493810000
F	0	-3.5086180000	1.6423870000	1.3089590000
H	0	-0.8033300000	4.0878270000	1.9021240000
H	0	-2.2228230000	3.6438970000	0.9069610000

Catalyst **Au1**

0 1

P	0.4332746174	0.0008578685	-0.0057358148
C	1.1306783057	-0.6197069609	1.5638117261
Au	-1.8491765452	-0.0417911357	-0.0546171643
C	0.5578236459	-1.7688352065	2.1350342866
H	-0.3051932897	-2.2421211309	1.6587772947
C	1.0882588237	-2.3037808705	3.3093803277
H	0.6407733466	-3.1988883331	3.7482303957
C	2.1845524767	-1.6890479992	3.9264795030
H	2.5951478811	-2.1046220737	4.8495384798
C	2.7503679482	-0.5401266844	3.3650650446
H	3.6030042804	-0.0556854510	3.8472911767
C	2.2280065510	-0.0042377350	2.1840580582
H	2.6751845470	0.8912689927	1.7481688517
C	1.1290877871	1.6737036259	-0.2265311988
C	0.4575060954	2.7631628480	0.3519215595
H	-0.4838569045	2.6021025929	0.8834907699
C	0.9889603644	4.0492658375	0.2429703328
H	0.4623820311	4.8934236904	0.6938298708
C	2.1872857705	4.2560603956	-0.4492937871
H	2.5991472380	5.2638871327	-0.5386653532

C	2.8538940710	3.1743230207	-1.0327574457
H	3.7871475763	3.3337632169	-1.5776423339
C	2.3286112691	1.8839846339	-0.9232742804
H	2.8526459178	1.0431665155	-1.3812052525
C	1.1967633412	-1.0227439435	-1.3111575006
C	2.3554783636	-1.7767437083	-1.0736836368
H	2.8118544510	-1.7840626031	-0.0822878466
C	2.9247595521	-2.5238061524	-2.1089303377
H	3.8249736635	-3.1129020049	-1.9190619362
C	2.3435334029	-2.5189895087	-3.3803263826
H	2.7889631328	-3.1061784678	-4.1866032368
C	1.1865036272	-1.7682887120	-3.6189985828
H	0.7264857544	-1.7679203753	-4.6097440057
C	0.6105673258	-1.0252574328	-2.5877308894
Cl	-4.1915859139	-0.0872197904	-0.1064423760
H	-0.3004715056	-0.4492360911	-2.7694223143

Activator **I**

0 1

N	0.9407832696	0.8173584948	1.2988632182
H	0.5923489855	1.7574710543	1.1177016026
O	1.3362251096	-2.4925951407	1.2452468641
F	-6.6330059827	-2.7179939590	0.4176683879
N	-2.1397064698	0.5245360527	0.5643181795
H	-1.8543673061	1.4847722802	0.7333072536
O	-1.8130642755	-2.8499325067	0.5189312554
F	-7.0273387658	-2.1819790032	-1.6393844175
F	-5.1734699151	-3.1738277677	-1.1116390064
F	-5.6325129187	3.8177578878	-0.7267022702
F	-7.3510695038	2.6611814606	-1.3684920586
F	-7.0105653797	3.0196921154	0.7360237305
C	0.1623118120	-0.2761097794	1.0969536007
C	0.4020614516	-1.7437479443	1.0829198307

C	-1.0842347123	-1.9133340829	0.7354715209
C	-1.1921836546	-0.4129170262	0.7556720573
C	-3.4813281558	0.3849477122	0.1787763851
C	-4.0737161486	-0.8579932655	-0.0859750710
H	-3.4984826323	-1.7796808716	0.0065439739
C	-5.4126418944	-0.9064071215	-0.4760274164
C	-6.1778975281	0.2536522795	-0.6128896641
H	-7.2173192792	0.2004942764	-0.9344454569
C	-5.5772629028	1.4847478204	-0.3424016716
C	-4.2417202710	1.5576959886	0.0490790229
H	-3.7863742656	2.5299354138	0.2450728157
C	-6.0600850516	-2.2496578510	-0.7083502723
C	-6.3937483004	2.7498237658	-0.4308786255
S	2.4276171518	0.8241592473	2.1498880426
O	2.5827887175	2.2163943364	2.5600769851
O	2.3838040867	-0.2824119159	3.0954262750
C	3.6504329932	0.4642699431	0.8978780491
C	3.9136027117	-0.8643311188	0.5696931801
C	4.3192229084	1.5304124186	0.2970168514
C	4.8713440072	-1.1224629637	-0.4138997778
H	3.3599713575	-1.6716883605	1.0529066826
C	5.2788177201	1.2470929267	-0.6756996969
H	4.0975815788	2.5561001698	0.5903263917
C	5.5552344875	-0.0755570998	-1.0355019820
H	6.3032192088	-0.2899318636	-1.7990382353
C	5.1240853207	-2.5507065832	-0.8356280661
C	6.0598879188	2.3706091243	-1.3141593595
F	5.3909818346	3.5329544838	-1.2604461646
F	7.2393165665	2.5605341577	-0.6962295891
F	6.3280125311	2.1113697418	-2.6042025973
F	5.0370257130	-3.3932411891	0.2045825266
F	4.2220512806	-2.9537397515	-1.7490628585
F	6.3395015912	-2.7003409861	-1.3855274256

ECa

0 1

P	-0.3059296944	2.9112395259	-0.9928818873
C	-0.5983853737	3.0848351546	0.7983427172
N	2.2079402734	-0.7884853768	0.2260545243
H	1.7057764630	-1.2762164394	-0.5271302062
O	2.9456272115	0.5216065137	3.2021991805
F	-4.8791130666	1.7039937882	3.0468244901
Au	-0.2923194369	0.7041367293	-1.5679394187
N	-0.7927667748	-1.4230747035	1.2355360586
H	-0.6504540387	-1.7672821895	0.2798688498
O	-0.0569098726	-0.2375086459	4.2586131761
F	-5.5193103438	0.2904362415	4.5549621093
C	0.4568019111	2.8188472264	1.6884803308
H	1.4515192558	2.5819670196	1.3066535520
F	-3.5352942117	1.1576767740	4.6532761157
C	0.2268969611	2.8482291553	3.0648033285
H	1.0476915107	2.6316422357	3.7509906433
F	-4.6028714404	-4.6335165287	0.8369208551
C	-1.0522077139	3.1241905481	3.5599970623
H	-1.2335550083	3.1225678315	4.6364570016
F	-6.1985889537	-3.8085380055	2.0504038629
C	-2.1012368170	3.3868253434	2.6751289014
H	-3.1013961762	3.5953321464	3.0571451357
F	-5.8967831739	-3.0941055409	0.0337633709
C	-1.8783130811	3.3695829546	1.2954823253
H	-2.7008090954	3.5758437163	0.6093367376
C	-1.5830786672	3.9067303509	-1.8275778818
C	-2.3937927788	3.3270925605	-2.8126219966
H	-2.2607647547	2.2765499945	-3.0739586620
C	-3.3694517206	4.0919136605	-3.4573484613
H	-3.9999972181	3.6322403154	-4.2212707813
C	-3.5373139643	5.4372263936	-3.1209120253
H	-4.3018441806	6.0345696060	-3.6227504948

C	-2.7260412342	6.0217917694	-2.1407437894
H	-2.8550989105	7.0738100990	-1.8774944277
C	-1.7502591847	5.2622147460	-1.4957875207
H	-1.1253130343	5.7256605397	-0.7301137989
C	1.2966264630	3.7120124956	-1.3284609200
C	1.7172749820	4.8354131420	-0.5986310085
H	1.0978365331	5.2390840325	0.2042051775
C	2.9504483230	5.4252657974	-0.8800383420
H	3.2780221819	6.2940903465	-0.3050092160
C	3.7677460836	4.8991497290	-1.8864232981
H	4.7368222589	5.3575904047	-2.0965031994
C	3.3496681288	3.7822593539	-2.6145963165
H	3.9903218911	3.3589616758	-3.3909997299
C	2.1172182727	3.1890084010	-2.3376672732
C	1.5691957767	-0.5832504036	1.4145547184
C	1.9619579362	0.0038079256	2.7209048387
C	0.5485274185	-0.3357756117	3.2211631716
C	0.2617246178	-0.8602375622	1.8408301333
C	-2.1201285000	-1.4224595125	1.6987980134
C	-2.5995661889	-0.4253584667	2.5577365469
H	-1.9494542264	0.3776023432	2.8954582671
C	-3.9325435342	-0.4567594965	2.9667236511
C	-4.8013298977	-1.4574116353	2.5284950606
H	-5.8386279961	-1.4750955732	2.8602715798
C	-4.3160110157	-2.4356729088	1.6584602089
C	-2.9855933673	-2.4257185360	1.2417756270
H	-2.6155852278	-3.2014598063	0.5702998438
C	-4.4639839398	0.6657824590	3.8194666694
C	-5.2529477741	-3.5003789291	1.1458782875
Cl	-0.1277009310	-1.6719649261	-1.9375829647
C	-3.9388778580	0.4098727793	-1.2796168954
C	-4.0386532364	0.9835192310	-0.2170408515
H	-4.1490168629	1.4748786744	0.7304295177
H	1.8067407671	2.2982088758	-2.8858677255
S	3.3785875619	0.2911806625	-0.3898682571

O	3.2553152143	0.1839444028	-1.8426979713
O	3.2259233506	1.5575044194	0.3214588136
C	4.9397451264	-0.4322697278	0.0877803356
C	5.3991861280	-0.2542904083	1.3923245363
C	5.6654708952	-1.1351622339	-0.8720490278
C	6.6268899992	-0.8217567928	1.7394972715
H	4.7968634317	0.2930325514	2.1220083631
C	6.8967571700	-1.6817739078	-0.5043616340
H	5.2759889892	-1.2448095955	-1.8838816072
C	7.3784606162	-1.5304675530	0.7983919461
H	8.3383566348	-1.9631991048	1.0797246607
C	-3.8262447019	-0.3010724727	-2.5587937295
H	-3.0675261557	0.1903689717	-3.1910634982
H	-4.7855425179	-0.2526376239	-3.0949151911
C	7.1164559585	-0.6983483776	3.1628845800
C	7.7349915298	-2.3923919728	-1.5394518635
F	6.9715714386	-2.9887733854	-2.4688295744
F	8.5454479996	-1.5333738644	-2.1845217035
F	8.5162423447	-3.3326497336	-0.9850191455
F	6.7884870611	0.4892370198	3.6944266112
F	6.5748917543	-1.6469483745	3.9487633045
F	8.4501328467	-0.8297411618	3.2443102583
N	-3.4994907785	-1.7034197381	-2.4013468191
C	-4.4155560457	-2.6807404544	-2.6460289494
H	-2.5647099792	-1.9242554548	-2.0737095838
O	-5.5461755547	-2.4285691034	-3.0535084431
C	-3.9811814226	-4.0934542742	-2.3674736430
C	-2.6574606039	-4.4642429389	-2.0822877570
C	-4.9793429195	-5.0786380608	-2.3746819385
C	-2.3462429523	-5.7943463125	-1.7902099797
H	-1.8485524248	-3.7324712659	-2.0979299531
C	-4.6686720163	-6.4057987381	-2.0828240252
H	-6.0019001893	-4.7732047100	-2.5982628144
C	-3.3505833437	-6.7666968780	-1.7846863129
H	-1.3126228494	-6.0713410920	-1.5705338661

H	-5.4568420351	-7.1623273125	-2.0821391681
H	-3.1051033038	-7.8056282116	-1.5519837735

ECb

0 1

P	0	1.1206043704	0.4759301664	2.5087024485
C	0	0.2077079497	1.9808559414	2.0130268001
N	0	-2.7948740485	-0.2279306270	0.1523593308
H	0	-2.1483001962	-1.0375449954	0.0692660737
O	0	-4.2150908292	2.7661836676	-0.3503908674
F	0	3.1815328690	4.4887707323	-0.8448357844
Au	0	1.6875077378	-1.0040209984	0.7745135541
N	0	-0.2993993576	0.6064964307	-1.6400839266
H	0	-0.3029677849	-0.3950662147	-1.4096879752
O	0	-1.5995700523	3.6753861529	-2.0746392449
F	0	3.9140533515	4.7713207803	-2.8654293699
C	0	-1.1480031124	2.1422319907	2.3279830593
H	0	-1.6924997228	1.3680668927	2.8676351691
F	0	1.8457227441	5.2094305066	-2.3831350626
C	0	-1.8249010789	3.2987459554	1.9297390538
H	0	-2.8875051508	3.3966344675	2.1548369375
F	0	4.4635980972	0.2776676212	-5.0408543505
C	0	-1.1527684991	4.3031471078	1.2302146498
H	0	-1.6865428279	5.2006257546	0.9120674622
F	0	5.1247234015	-0.3681660272	-3.0900562423
C	0	0.2003505393	4.1432511563	0.9111857594
H	0	0.7294768297	4.9194274236	0.3552171200
F	0	3.5213775852	-1.4278007872	-4.0936666405
C	0	0.8766494028	2.9834837346	1.2888506597
H	0	1.9309896929	2.8701688254	1.0325941320
C	0	2.6435130769	1.0980837788	3.3103628619
C	0	3.7928603858	0.2922238045	3.3017328222
H	0	3.7692628510	-0.6849097583	2.8161773582

C	0	4.9741723296	0.7434063576	3.8909279701
H	0	5.8661391084	0.1148002579	3.8619811401
C	0	5.0182332883	2.0043065036	4.4930709262
H	0	5.9466549148	2.3634739701	4.9427995366
C	0	3.8745899675	2.8085379926	4.5132306553
H	0	3.9043598505	3.7935893648	4.9841169353
C	0	2.6891798545	2.3594365135	3.9259868229
H	0	1.8046835929	2.9980665426	3.9384118600
C	0	0.1039000157	-0.2773843271	3.8221607192
C	0	0.2504665163	0.0979401273	5.1669280905
H	0	1.0235220037	0.8104437496	5.4589236503
C	0	-0.5968694036	-0.4436656263	6.1354812454
H	0	-0.4773683166	-0.1532196240	7.1816928838
C	0	-1.5987557799	-1.3483515822	5.7669872447
H	0	-2.2656866714	-1.7624959348	6.5267399144
C	0	-1.7464461160	-1.7219623754	4.4285326700
H	0	-2.5350729470	-2.4112920064	4.1248024389
C	0	-0.8893806877	-1.1993473300	3.4572225399
C	0	-2.5018930994	0.9299419511	-0.4878948301
C	0	-3.1679166277	2.2489266036	-0.6693298674
C	0	-1.9459420100	2.6735449381	-1.4960737952
C	0	-1.3879815156	1.3008166090	-1.2586779184
C	0	0.8988504118	1.1232021184	-2.1514405863
C	0	1.2347834233	2.4753622165	-2.0175960546
H	0	0.5358746364	3.1762934678	-1.5725952751
C	0	2.4770365079	2.9295725935	-2.4647516443
C	0	3.3889617764	2.0676151805	-3.0691259253
H	0	4.3580271712	2.4321042116	-3.4076600463
C	0	3.0349582121	0.7218789580	-3.2179686290
C	0	1.8099174662	0.2418229375	-2.7622436066
H	0	1.5752856547	-0.8192605218	-2.8583165513
C	0	2.8561379299	4.3536497912	-2.1594042562
C	0	4.0327046847	-0.2082460323	-3.8606523675
Cl	0	-0.5166263832	-2.3219397357	-0.2108312728
C	0	3.0495954795	-2.1181782161	-0.5957718866

C	0	3.7497651479	-1.1539845250	-0.2324349368
H	0	-1.0045754716	-1.5058510312	2.4162026553
S	0	-4.0317361862	-0.4513185387	1.2872413825
O	0	-3.7134988607	-1.7388260419	1.9024680949
O	0	-4.1648165885	0.7741286759	2.0703387571
C	0	-5.5151157468	-0.6621040147	0.3107491491
C	0	-6.2386451409	0.4611014904	-0.0856061844
C	0	-5.9117030043	-1.9588945731	-0.0143920259
C	0	-7.3919495304	0.2670638422	-0.8489193417
H	0	-5.8851956289	1.4616744985	0.1689454655
C	0	-7.0739870123	-2.1286106862	-0.7687935747
H	0	-5.3254972330	-2.8138456042	0.3220697105
C	0	-7.8143183734	-1.0202641280	-1.1891758375
H	0	-8.7187030414	-1.1601613136	-1.7812366803
C	0	-8.1554933321	1.4689326691	-1.3514515698
C	0	-7.5615202760	-3.5215064684	-1.0867280624
F	0	-6.5435780560	-4.3908092296	-1.1881424658
F	0	-8.3826517952	-3.9837899123	-0.1259685141
F	0	-8.2461766049	-3.5551819443	-2.2411385674
F	0	-8.1007218855	2.4884750067	-0.4811181883
F	0	-7.6505734553	1.9142822763	-2.5166069357
F	0	-9.4499455645	1.1817303179	-1.5659093338
H	0	0.8380097794	-2.9575691913	-1.7653717855
O	0	1.6567775435	-3.0696596971	-2.2950095978
C	0	2.7122423219	-3.3261271777	-1.3893068628
C	0	2.3851075696	-4.5433602854	-0.4822746629
C	0	3.6790408188	-4.9234261030	-3.1294125195
C	0	2.0960181546	-5.7872354470	-1.3275759303
H	0	3.2423317016	-4.7173812896	0.1893078344
H	0	1.5212627794	-4.2793897663	0.1459264725
C	0	3.2624750373	-6.1304175203	-2.2585095015
H	0	4.5826209872	-5.1381026108	-3.7162045588
H	0	2.8559472222	-4.6547124841	-3.8087050883
H	0	1.8814868039	-6.6374722944	-0.6613650402
H	0	1.1865201335	-5.6110481195	-1.9230676001

H	0	3.0023066420	-6.9784276801	-2.9107084030
H	0	4.1326379577	-6.4460598250	-1.6573693593
C	0	3.9554835336	-3.7485677494	-2.2254902515
O	0	5.0457743894	-3.2561797055	-2.0566402073
C	0	4.7318671515	-0.1537043021	0.0650307418
C	0	4.3958761761	1.2129402423	0.0591168434
C	0	6.0490117539	-0.5471032361	0.3660654588
C	0	5.3591712724	2.1717045311	0.3578569589
H	0	3.3745710258	1.5077043897	-0.1780113046
C	0	7.0061744292	0.4199525391	0.6721009917
H	0	6.3062831098	-1.6068846007	0.3509730916
C	0	6.6640385539	1.7765993421	0.6730186182
H	0	5.0876513257	3.2284487089	0.3452393311
H	0	8.0265244018	0.1126248208	0.9111583732
H	0	7.4171596022	2.5287336179	0.9178924428

FCa

0 1

N	1.9353327593	-0.0834040474	0.0111109572
H	1.9174364267	-0.2178437040	1.0485718269
O	2.5251675481	1.8137055366	-2.6987607117
F	-4.6369055554	4.3718917462	-0.5507038013
N	0.0102237932	2.0975900045	1.2042736912
H	0.3424175713	1.4243312141	1.9167553654
O	0.5331819507	4.1025863265	-1.4573277257
F	-4.3016808864	6.2354580666	0.4927408874
F	-2.9152138857	5.5832923050	-1.0421465752
F	-2.3494943573	4.6501811893	5.4521223374
F	-4.3050515310	3.9352702150	4.8656313854
F	-2.7261353779	2.5236050141	5.3292372066
C	1.5504783437	1.1230967042	-0.4905366892
C	1.8343117357	1.9209012484	-1.7096612589
C	0.8990865739	3.0012658920	-1.1299346110

C	0.6807719889	2.0823575931	0.0416574509
C	-1.0580376830	2.9221102271	1.5603850739
C	-1.7773073831	3.6668298140	0.6136417471
H	-1.4852667586	3.6627697745	-0.4329540498
C	-2.8705977166	4.4280075361	1.0232492145
C	-3.2752691510	4.4575324280	2.3589369042
H	-4.1435619101	5.0394799922	2.6635497787
C	-2.5586360547	3.7041200271	3.2908550198
C	-1.4545475327	2.9455470717	2.9062022867
H	-0.9070068797	2.3483507232	3.6371439456
C	-3.6770839869	5.1626937663	-0.0168796064
C	-2.9844410105	3.7054666479	4.7361721338
Cl	1.6700563994	0.1716386238	3.1022766112
S	2.3645397137	-1.3997722957	-0.9429551579
O	2.0451525012	-2.5807482546	-0.1406757559
O	1.7777634220	-1.1845202382	-2.2623656162
C	4.1451080593	-1.3148731738	-1.0724650980
C	4.7154999559	-0.3879201336	-1.9472109858
C	4.9114187353	-2.1837829256	-0.2999326991
C	6.1062666616	-0.3344010094	-2.0315337199
H	4.0828069443	0.2865527837	-2.5309685073
C	6.3045539498	-2.1129989062	-0.4048407725
H	4.4270626709	-2.9036400060	0.3604457034
C	6.9034584338	-1.1949019621	-1.2678599021
H	7.9891242771	-1.1551500264	-1.3534215090
C	6.7609259741	0.6898857160	-2.9273765149
C	7.1489615944	-3.0332392612	0.4432543956
F	7.1542284476	-2.6426576646	1.7328912794
F	6.6772958018	-4.2921159871	0.4197019169
F	8.4248251315	-3.0683669443	0.0335133823
F	5.9668049559	1.0347420104	-3.9512043646
F	7.0495686551	1.8164094086	-2.2497349703
F	7.9158716075	0.2324735702	-3.4398179082
C	-0.6927491938	-3.6179959699	0.8996853409
C	-1.4785523400	-3.9459801623	-0.1184186194

H	-1.3146105332	-4.9219915615	-0.5885261773
C	-0.6298736152	-2.3634839354	1.7442886407
H	-1.4922095996	-2.2530394418	2.4215337819
H	-0.5477900931	-1.4478583233	1.1380096777
N	0.5990647408	-2.5765710888	2.4943481275
C	1.1543612542	-3.7127201954	2.1615003074
O	0.4068699013	-4.4350896755	1.3569285111
C	2.4805459157	-4.1680488594	2.5463286145
C	3.3731753038	-3.2762633995	3.1719477970
C	2.8940879188	-5.4689559778	2.2113741516
C	4.6701227022	-3.6931521676	3.4583686680
H	3.0704493012	-2.2512986456	3.3978948176
C	4.1925390927	-5.8770620640	2.5098906877
H	2.1978865246	-6.1438990397	1.7125150635
C	5.0798846558	-4.9906473440	3.1293348990
H	5.3717591667	-2.9985231553	3.9222614799
H	4.5181912759	-6.8856536451	2.2488013537
H	6.1028724694	-5.3060990833	3.3434921035
H	1.0611783758	-1.7677773196	2.9624097223
Au	-2.7057899873	-2.4487893691	-0.8353766386
P	-3.8197127634	-0.4930999303	-1.5122597713
C	-2.6796829639	0.6764317533	-2.3297158175
C	-4.5023782236	0.4286464768	-0.0854240569
C	-5.2297518103	-0.7139406230	-2.6529209206
C	-1.4951733443	0.1861834218	-2.8997774971
C	-2.9788165904	2.0471547441	-2.4177909638
C	-3.8059391464	0.3784759107	1.1345938105
C	-5.6752984776	1.1912211382	-0.1806370369
C	-5.3581951627	0.0007986106	-3.8515776059
C	-6.2094457639	-1.6571260056	-2.2964224577
H	-1.2380018788	-0.8709860687	-2.8053830313
C	-0.6277162657	1.0530546405	-3.5694980721
C	-2.1150660232	2.9063909479	-3.0976956806
H	-3.8780665404	2.4461707409	-1.9465197725
H	-2.8936864965	-0.2161145881	1.2165357837

C	-4.2805717320	1.0795668829	2.2424313063
C	-6.1382184159	1.9043915760	0.9288059221
H	-6.2290588368	1.2306027520	-1.1204783007
H	-4.5989696544	0.7281294071	-4.1420792280
C	-6.4640517034	-0.2183160716	-4.6796072420
C	-7.3142319786	-1.8660584148	-3.1218278090
H	-6.1049646398	-2.2276044524	-1.3697150131
H	0.3065791643	0.6683449406	-3.9800104049
C	-0.9424598672	2.4096286150	-3.6784308381
H	-2.3448530534	3.9721317787	-3.1503043351
H	-3.7401860974	1.0289791054	3.1885182683
C	-5.4441310836	1.8489700805	2.1396639805
H	-7.0448563940	2.5071529003	0.8434738374
H	-6.5582257568	0.3409423233	-5.6131802248
C	-7.4429804417	-1.1461853797	-4.3156681503
H	-8.0741266708	-2.5971045630	-2.8368032693
H	-0.2525820909	3.0900954528	-4.1806305791
H	-5.8020674079	2.4086004588	3.0062246052
H	-8.3057870483	-1.3139637733	-4.9643007365

FCb

0 1

C	0	-1.3021853199	4.2985313238	0.8068412015
C	0	-1.6381760784	5.7563796018	0.4789237088
C	0	-1.9169477195	5.7377992511	-1.0365497916
C	0	-1.0754991837	4.5654356087	-1.6061945215
H	0	-0.7650771723	6.3892829957	0.7020570481
H	0	-2.4848918747	6.1353652646	1.0693933606
H	0	-2.9829960377	5.5435108610	-1.2202694185
H	0	-1.6683696212	6.6952786855	-1.5156136817
H	0	-1.7069986010	3.8355117919	-2.1290708443
H	0	-0.2852479408	4.8836278272	-2.2993965254
C	0	-0.4294825505	3.8747586684	-0.3824109512
O	0	0.9081709849	4.3966285418	-0.1779096366

C	0	1.7750790228	3.3842942354	-0.2215523054
C	0	1.1936455142	2.1202610309	-0.4509081091
Au	0	2.1793646992	0.3101811873	-0.6733702247
P	0	3.7347133440	-1.4433175636	-0.7996984282
C	0	3.1991674746	-3.1496856721	-1.1317582842
C	0	2.0362522110	-3.3396797347	-1.8913152227
C	0	3.9060871624	-4.2611605696	-0.6467335148
C	0	1.5781989333	-4.6314580382	-2.1551395213
H	0	1.4671766746	-2.4784311289	-2.2449406497
C	0	3.4437001161	-5.5507540238	-0.9147782788
H	0	4.8060011064	-4.1234556545	-0.0447727680
C	0	2.2783653503	-5.7373960286	-1.6655156627
H	0	0.6612636238	-4.7716244837	-2.7317789592
H	0	3.9916307790	-6.4134054608	-0.5289739348
H	0	1.9126530421	-6.7478433717	-1.8625862585
C	0	5.0414665656	-1.0572841407	-2.0253632418
C	0	5.8784148043	-2.0584004863	-2.5420394331
C	0	5.2348898922	0.2770523618	-2.4190375532
C	0	6.8991675239	-1.7255781070	-3.4350805335
H	0	5.7316350653	-3.1004202582	-2.2532837780
C	0	6.2622298098	0.6068012694	-3.3066442345
H	0	4.5818502843	1.0599356077	-2.0266101824
C	0	7.0948840764	-0.3937257298	-3.8160718552
H	0	7.5440114030	-2.5108252976	-3.8360431175
H	0	6.4070297722	1.6479225993	-3.6034946437
H	0	7.8943580191	-0.1371142109	-4.5149313031
C	0	4.6095589578	-1.4680689362	0.8116341674
C	0	4.0653809768	-2.1803052070	1.8936653652
C	0	5.7294493686	-0.6476264059	1.0155017718
C	0	4.6344946150	-2.0615095162	3.1635361495
H	0	3.1974219682	-2.8245241290	1.7445099971
C	0	6.2906383882	-0.5306506940	2.2898989215
H	0	6.1637309649	-0.0950083237	0.1806168531
C	0	5.7425769218	-1.2329772691	3.3670406259
H	0	4.2049428450	-2.6184183358	3.9995433040

H	0	7.1614914182	0.1119459381	2.4394912893
H	0	6.1808232216	-1.1379775975	4.3630725055
C	0	3.1747599991	3.7614068153	-0.0343504053
C	0	4.1403031933	2.8284409242	0.3925126452
C	0	3.5665988380	5.0948683586	-0.2739681772
C	0	5.4672823091	3.2179060177	0.5574760373
H	0	3.8439060522	1.8052640418	0.6177113427
C	0	4.8976161924	5.4749134809	-0.1182977126
H	0	2.8204337794	5.8209541502	-0.5975417464
C	0	5.8512162295	4.5375641606	0.2955364804
H	0	6.2034618496	2.4880996023	0.8995374318
H	0	5.1945668054	6.5061045254	-0.3202295465
H	0	6.8940307637	4.8381362297	0.4199230691
C	0	-0.1676390253	2.4085483926	-0.5369874486
O	0	-1.1792700735	1.6211699144	-0.7269995387
H	0	-0.9010230900	0.6746469240	-0.9625976636
Cl	0	-0.6603736879	-1.0366873713	-1.8146999619
H	0	-0.7683159476	-2.8224282682	-0.6293174550
H	0	-2.6422144379	-1.4678538988	-1.2449661675
N	0	-1.0818687122	-3.5410479696	0.0538675815
N	0	-3.5664590196	-1.8218424868	-0.9409512621
C	0	-2.4432756455	-3.7854012460	0.0842654878
S	0	-0.2446413856	-3.3748139790	1.5215358312
C	0	-3.5371688870	-3.0236395218	-0.3494890396
C	0	-4.6142162093	-0.9031871591	-1.0604290176
C	0	-3.2791709098	-4.8964659800	0.5980833510
O	0	-0.9546874511	-4.1813731483	2.5097173379
O	0	1.1740219850	-3.5764048020	1.2446810416
C	0	-0.4753272022	-1.6380736254	1.9360131891
C	0	-4.5129885531	-4.0511863619	0.1810586870
C	0	-5.9399486629	-1.1899063703	-0.7070435079
C	0	-4.2776688609	0.3806020584	-1.5174868146
O	0	-3.1148825446	-5.9736353795	1.1121944411
C	0	0.6307488577	-0.8571433631	2.2569408999
C	0	-1.7723652289	-1.1217097431	1.9761279702

O	0	-5.7105896225	-4.1553905789	0.2634807090
H	0	-6.2184784402	-2.1885866321	-0.3689602758
C	0	-6.9017471885	-0.1819208697	-0.7940102343
C	0	-5.2510462502	1.3737927935	-1.5806840780
H	0	-3.2494107408	0.6034214633	-1.7998737305
C	0	0.4240563151	0.4734781652	2.6384307332
H	0	1.6342719371	-1.2764355226	2.2204472815
C	0	-1.9562574420	0.2172359534	2.3080142021
H	0	-2.6344738705	-1.7481115709	1.7600110173
C	0	-6.5744336940	1.1067799621	-1.2225573375
C	0	-8.3122343092	-0.4830154990	-0.3534889884
C	0	-4.8418841385	2.7700863870	-1.9689430646
C	0	-0.8604590908	1.0165951860	2.6532459652
C	0	1.5857730752	1.3122293231	3.1167843015
C	0	-3.3421010135	0.8180101769	2.2961384825
H	0	-7.3354802957	1.8834518875	-1.2830035818
F	0	-8.4651788865	-0.2876048396	0.9715917023
F	0	-9.2102526286	0.3047907365	-0.9702770993
F	0	-8.6549732440	-1.7583197035	-0.6014400961
F	0	-5.8562042478	3.4700537915	-2.4982996361
F	0	-4.4026520917	3.4700452083	-0.8970773519
F	0	-3.8360294253	2.7714296726	-2.8643951928
H	0	-1.0131218311	2.0560947086	2.9437135137
F	0	2.7642589837	0.8462905323	2.6755696981
F	0	1.4770751248	2.5944319179	2.7176778061
F	0	1.6446721497	1.3290523924	4.4600002560
F	0	-4.2939788173	-0.1177274769	2.1562529212
F	0	-3.5934204661	1.4962087734	3.4265142345
F	0	-3.4914528496	1.6882092824	1.2769670229
H	0	-0.7789261286	4.1501864754	1.7616393869
H	0	-2.2059819935	3.6720777058	0.7934951734

IN1a

0 1

P	-1.9242233480	2.8811429849	0.0992301314
C	-1.2515983740	1.9476032461	1.5192197037
N	2.4903456185	-0.0586739742	0.4388174777
H	1.8268129898	0.2238755653	-0.3078150794
O	3.9969371821	-2.0251875185	2.6811132359
F	-2.4521663590	-4.3507452166	4.5544159578
Au	-2.4531079711	1.5006594470	-1.7327219852
N	-0.2724904133	-1.4790192573	0.9298137976
H	-0.2621438026	-0.7908223033	0.1617231106
O	1.1308934306	-3.4812312337	3.2827626741
F	-4.4950624404	-4.6297924908	3.8848059393
C	0.1054135511	2.0043537069	1.8694881064
H	0.8048126454	2.6098128783	1.2920479380
F	-2.8894537141	-5.7592669465	2.9751204522
C	0.5712442568	1.2730026275	2.9657156700
H	1.6313139536	1.3138909144	3.2216232644
F	-6.2262721454	-1.8989086846	0.3313132415
C	-0.3085527263	0.4839414575	3.7121078391
H	0.0618665981	-0.0904506855	4.5640330373
F	-5.0870201202	-0.0666622136	0.1158495049
C	-1.6591275992	0.4164607487	3.3560456944
H	-2.3464617303	-0.2152763076	3.9221345776
F	-4.9001892317	-1.6355085066	-1.3605871250
C	-2.1305054016	1.1393811092	2.2599746724
H	-3.1832533247	1.0702598024	1.9784980771
C	-3.3821084585	3.7586772347	0.7576752153
C	-4.4426629813	4.0678021295	-0.1070927490
H	-4.4059152012	3.7410995578	-1.1485786576
C	-5.5428571695	4.7886260938	0.3622624936
H	-6.3659404241	5.0243542807	-0.3157013779
C	-5.5923030052	5.1987933624	1.6979419945
H	-6.4561657145	5.7565616340	2.0664746818

C	-4.5377160378	4.8913101777	2.5640160406
H	-4.5742142066	5.2101867323	3.6079470835
C	-3.4332387215	4.1754761505	2.0981498401
H	-2.6129755770	3.9399426996	2.7781598928
C	-0.6975456949	4.1754235414	-0.2987887658
C	-0.8720186768	5.4999379512	0.1332678834
H	-1.7597611320	5.7842538811	0.6990991091
C	0.0943815498	6.4631689135	-0.1659409101
H	-0.0482684496	7.4925528654	0.1704792544
C	1.2387977729	6.1115590938	-0.8883692956
H	1.9950224686	6.8665641345	-1.1154479331
C	1.4128290118	4.7933803152	-1.3198374217
H	2.3082503801	4.5088152153	-1.8757899083
C	0.4460496709	3.8269529082	-1.0371256572
C	2.1495958906	-1.0736567395	1.2783174193
C	2.8632464100	-1.9095982067	2.2691474118
C	1.5168487879	-2.6040145475	2.5469992672
C	0.9165172922	-1.7095771455	1.4967702602
C	-1.5193779607	-2.0329957141	1.2126549397
C	-1.7312192382	-2.9998388816	2.2035166840
H	-0.8946719553	-3.3844815357	2.7870098699
C	-3.0264133857	-3.4596991319	2.4424686379
C	-4.1222647643	-2.9720769192	1.7261718077
H	-5.1289777621	-3.3288914338	1.9346530081
C	-3.8959943513	-2.0060075596	0.7443096837
C	-2.6088741866	-1.5497460481	0.4771598471
H	-2.4425582658	-0.7959374071	-0.2916229666
C	-3.2207539496	-4.5456672370	3.4685496881
C	-5.0342172111	-1.4122198566	-0.0403902776
Cl	0.1824863113	0.3103605489	-1.6553659963
C	-3.1743830093	0.2012494710	-3.4879068219
C	-4.0863103244	0.9647968794	-3.1462701353
H	-5.0273216922	1.4818869900	-3.0340395806
H	0.5886797634	2.7992496432	-1.3751361979
S	3.6737654607	1.0980244946	0.7906468924

O	3.2308012733	2.3142355836	0.1080173992
O	3.9111074822	1.0620988794	2.2326735370
C	5.1416686807	0.4973314215	-0.0326748169
C	5.8384813867	-0.5744349622	0.5263032717
C	5.5514274927	1.1261121879	-1.2061102175
C	6.9783537131	-1.0328921004	-0.1364265528
H	5.4816771452	-1.0505182771	1.4443791775
C	6.7032034119	0.6586724208	-1.8431246312
H	4.9813011711	1.9632057450	-1.6078318139
C	7.4157367637	-0.4201746454	-1.3145004004
H	8.3109460033	-0.7846132288	-1.8189419240
C	-2.3179316018	-0.8228113771	-4.1317836055
H	-1.5525759834	-0.3126870008	-4.7364813611
H	-2.9632946869	-1.4063103898	-4.8054615680
C	7.7139614515	-2.2389353350	0.3972916875
C	7.2107508357	1.3545073812	-3.0827792980
F	6.2211616399	1.9742237586	-3.7449407520
F	8.1299239696	2.2887211222	-2.7774510018
F	7.7934754045	0.4912158687	-3.9305206891
F	7.6159438642	-2.3310971810	1.7314433167
F	7.2150247360	-3.3781120790	-0.1181189079
F	9.0194919792	-2.2033397077	0.0832182655
N	-1.6859796096	-1.7276301355	-3.2053693491
C	-2.2556217528	-2.9191729308	-2.8654684657
H	-0.9175221006	-1.3370001134	-2.6569201419
O	-3.3007924727	-3.3186687506	-3.3673479962
C	-1.5169763052	-3.7113930843	-1.8253559711
C	-0.1470568626	-3.5494146946	-1.5700083305
C	-2.2484837112	-4.6554317726	-1.0874591778
C	0.4755643744	-4.3083268921	-0.5760662629
H	0.4438235307	-2.8287994860	-2.1377855634
C	-1.6299448760	-5.3986627446	-0.0852905297
H	-3.3117503197	-4.7713341894	-1.3011027231
C	-0.2655762442	-5.2227370594	0.1760823355
H	1.5420426962	-4.1755637212	-0.3823262030

H	-2.2128963302	-6.1000152960	0.5132604927
H	0.2168860643	-5.7929058994	0.9728886042

IN1b

0 1

C	0	-0.6090490000	2.8506740000	0.6192370000
C	0	-1.8766950000	3.7393170000	0.6156750000
C	0	-1.9082010000	4.7808100000	-0.5157180000
C	0	-0.5451100000	5.4801680000	-0.6807580000
H	0	-1.9417760000	4.2501980000	1.5887010000
H	0	-2.7747860000	3.1130180000	0.5393010000
H	0	-2.1563670000	4.2807150000	-1.4581390000
H	0	-2.6852890000	5.5339480000	-0.3290700000
H	0	-0.4771500000	5.9552120000	-1.6775280000
H	0	-0.3516100000	6.2740280000	0.0586980000
C	0	0.5215250000	4.4743640000	-0.5930530000
O	0	1.6970470000	4.7328130000	-0.1815340000
C	0	2.4713530000	3.4689690000	-0.0935490000
C	0	1.6793010000	2.4319240000	-0.4203150000
Au	0	2.2762500000	0.4803120000	-0.7015790000
P	0	3.4610860000	-1.5003210000	-1.1338420000
C	0	2.5591310000	-2.9919210000	-1.6532940000
C	0	1.2790350000	-2.8291270000	-2.2024690000
C	0	3.1036590000	-4.2803430000	-1.5251360000
C	0	0.5484480000	-3.9455000000	-2.6139840000
H	0	0.8357200000	-1.8341530000	-2.2744820000
C	0	2.3676660000	-5.3919370000	-1.9370030000
H	0	4.0932420000	-4.4211510000	-1.0868680000
C	0	1.0892260000	-5.2264160000	-2.4798450000
H	0	-0.4542450000	-3.8121070000	-3.0256890000
H	0	2.7914110000	-6.3926710000	-1.8269530000
H	0	0.5115540000	-6.0998240000	-2.7911780000
C	0	4.7200180000	-1.1675630000	-2.4288260000
C	0	5.2730170000	-2.1989850000	-3.2032820000

C	0	5.1652260000	0.1515870000	-2.6188480000
C	0	6.2629510000	-1.9143720000	-4.1465600000
H	0	4.9278430000	-3.2262320000	-3.0799420000
C	0	6.1618150000	0.4306730000	-3.5574470000
H	0	4.7288400000	0.9628880000	-2.0316440000
C	0	6.7118670000	-0.6013950000	-4.3227920000
H	0	6.6841420000	-2.7232280000	-4.7478900000
H	0	6.5025930000	1.4594060000	-3.6940110000
H	0	7.4863020000	-0.3826020000	-5.0615680000
C	0	4.4457830000	-1.9504190000	0.3464060000
C	0	3.9571580000	-2.8719040000	1.2859780000
C	0	5.6312000000	-1.2483420000	0.6173050000
C	0	4.6486590000	-3.0812250000	2.4826330000
H	0	3.0343970000	-3.4197000000	1.0931120000
C	0	6.3149650000	-1.4611960000	1.8156900000
H	0	6.0205750000	-0.5275860000	-0.1039540000
C	0	5.8241860000	-2.3757390000	2.7530560000
H	0	4.2598770000	-3.7996220000	3.2081530000
H	0	7.2362660000	-0.9095690000	2.0170900000
H	0	6.3589990000	-2.5390990000	3.6914010000
C	0	3.8590370000	3.6803910000	0.3046800000
C	0	4.6552440000	2.5884420000	0.7059150000
C	0	4.4352230000	4.9649590000	0.2841670000
C	0	5.9915180000	2.7759930000	1.0511520000
H	0	4.2183830000	1.5920880000	0.7565500000
C	0	5.7737640000	5.1462880000	0.6374590000
H	0	3.8338420000	5.8238610000	-0.0150720000
C	0	6.5595500000	4.0547840000	1.0176700000
H	0	6.5892450000	1.9155170000	1.3604380000
H	0	6.2049090000	6.1497110000	0.6125610000
H	0	7.6065890000	4.1997480000	1.2930210000
C	0	0.3015390000	3.0034050000	-0.6821810000
O	0	-0.3671400000	2.5989530000	-1.8299250000
H	0	-0.6052930000	1.6499000000	-1.7036480000
Cl	0	-0.9664510000	-0.3033150000	-1.0209950000

H	0	-1.1225270000	-2.4121090000	-0.4691950000
H	0	-2.9442450000	-0.8792500000	-0.6928240000
N	0	-1.4385290000	-3.2444120000	0.0633350000
N	0	-3.8818900000	-1.2972380000	-0.5444760000
C	0	-2.7998730000	-3.4270900000	0.1491970000
S	0	-0.4489560000	-3.5015600000	1.4238980000
C	0	-3.8774920000	-2.5741540000	-0.1417980000
C	0	-4.9487820000	-0.4285540000	-0.7830810000
C	0	-3.6661850000	-4.5774520000	0.5002020000
O	0	-1.1952610000	-4.3622330000	2.3363810000
O	0	0.8811610000	-3.8654900000	0.9477350000
C	0	-0.3524090000	-1.8516400000	2.1262070000
C	0	-4.8742970000	-3.6495630000	0.2254990000
C	0	-6.2952690000	-0.8045680000	-0.6661730000
C	0	-4.6239640000	0.8857970000	-1.1593960000
O	0	-3.5317880000	-5.7278880000	0.8350070000
C	0	0.8913230000	-1.2612660000	2.3410980000
C	0	-1.5420630000	-1.1862660000	2.4136570000
O	0	-6.0753170000	-3.7518700000	0.2713070000
H	0	-6.5619870000	-1.8237540000	-0.3841500000
C	0	-7.2925460000	0.1378030000	-0.9204780000
C	0	-5.6384490000	1.8088060000	-1.4062250000
H	0	-3.5755630000	1.1710670000	-1.2610610000
C	0	0.9336930000	0.0234840000	2.8857430000
H	0	1.8069060000	-1.7915620000	2.0869460000
C	0	-1.4801680000	0.1150120000	2.9117010000
H	0	-2.5069160000	-1.6612770000	2.2346950000
C	0	-6.9828950000	1.4482100000	-1.2923830000
C	0	-8.7358010000	-0.2605390000	-0.7386360000
C	0	-5.2659820000	3.2254220000	-1.7567780000
C	0	-0.2472130000	0.7147540000	3.1682530000
C	0	2.2539700000	0.6774430000	3.2133490000
C	0	-2.7690020000	0.8771480000	3.0932060000
H	0	-7.7718210000	2.1695250000	-1.4996870000
F	0	-9.1428620000	-0.0669490000	0.5319470000

F	0	-9.5571400000	0.4576200000	-1.5246320000
F	0	-8.9393380000	-1.5582800000	-1.0201370000
F	0	-6.2525940000	3.8693970000	-2.3973790000
F	0	-4.9823310000	3.9467940000	-0.6482670000
F	0	-4.1715680000	3.2778190000	-2.5395060000
H	0	-0.2087840000	1.7255060000	3.5718800000
F	0	3.2793450000	0.1047240000	2.5657990000
F	0	2.2474090000	1.9857770000	2.8948190000
F	0	2.5217020000	0.5998310000	4.5297910000
F	0	-3.6009120000	0.2630540000	3.9463640000
F	0	-2.5614910000	2.1234940000	3.5435530000
F	0	-3.4258440000	0.9827840000	1.9156180000
H	0	0.0162070000	3.0396320000	1.5013860000
H	0	-0.8502240000	1.7788150000	0.6112080000

IN2b

0 1

C	0	-0.5994375110	2.8507395909	0.6280969616
C	0	-1.8646636115	3.7428304247	0.6327163946
C	0	-1.8984962448	4.7874715254	-0.4956982282
C	0	-0.5343649642	5.4838054772	-0.6648670684
H	0	-1.9239325638	4.2512616354	1.6073965385
H	0	-2.7647906720	3.1191716388	0.5587471553
H	0	-2.1520978520	4.2905855047	-1.4383671993
H	0	-2.6728232425	5.5420714524	-0.3035711347
H	0	-0.4695809713	5.9613603822	-1.6606497211
H	0	-0.3355767240	6.2751778776	0.0758531141
C	0	0.5300647693	4.4750624475	-0.5845870420
O	0	1.7080576103	4.7294008471	-0.1775984666
C	0	2.4794510106	3.4633474148	-0.0964424797
C	0	1.6833365883	2.4292049601	-0.4225184824
Au	0	2.2746550538	0.4769931121	-0.7114817777
P	0	3.4532170890	-1.5050816091	-1.1539908956

C	0	2.5454798737	-2.9926667945	-1.6748086208
C	0	1.2631466369	-2.8251536273	-2.2172816399
C	0	3.0878337047	-4.2827060317	-1.5537983105
C	0	0.5281571031	-3.9384936171	-2.6291855833
H	0	0.8216661197	-1.8289756844	-2.2837369623
C	0	2.3474708331	-5.3912415647	-1.9660625547
H	0	4.0792625740	-4.4271391779	-1.1208951488
C	0	1.0667902511	-5.2210325653	-2.5021555763
H	0	-0.4762045200	-3.8014835177	-3.0355969628
H	0	2.7695672377	-6.3932725368	-1.8616224354
H	0	0.4857350355	-6.0920880636	-2.8137845888
C	0	4.7077757921	-1.1708348755	-2.4528169676
C	0	5.2489437278	-2.1995096104	-3.2391216706
C	0	5.1612875794	0.1467505303	-2.6343151881
C	0	6.2355160011	-1.9139185003	-4.1856796610
H	0	4.8971315542	-3.2252736006	-3.1224090965
C	0	6.1545041067	0.4266743940	-3.5761831670
H	0	4.7342181303	0.9562590698	-2.0378703061
C	0	6.6928065051	-0.6027577498	-4.3533986744
H	0	6.6474455393	-2.7206285953	-4.7962380316
H	0	6.5018449309	1.4540837086	-3.7059991688
H	0	7.4645422325	-0.3831947700	-5.0947650634
C	0	4.4423456236	-1.9630177469	0.3209544550
C	0	3.9539922127	-2.8860145223	1.2592034419
C	0	5.6312979862	-1.2661702157	0.5897886980
C	0	4.6491636144	-3.1018865703	2.4525484341
H	0	3.0286689141	-3.4299980864	1.0678312298
C	0	6.3187585419	-1.4855723845	1.7848945654
H	0	6.0207792878	-0.5445298916	-0.1304910902
C	0	5.8281950276	-2.4015045820	2.7209998803
H	0	4.2605303118	-3.8214103473	3.1770318779
H	0	7.2428177492	-0.9379821609	1.9846594487
H	0	6.3659129159	-2.5699705831	3.6567779192
C	0	3.8694923627	3.6700077173	0.2959892272
C	0	4.6639531428	2.5750432053	0.6924105264

C	0	4.4496839138	4.9527382531	0.2743218353
C	0	6.0024293127	2.7578387481	1.0316282584
H	0	4.2239861369	1.5801091491	0.7439908403
C	0	5.7904216045	5.1293159705	0.6216478830
H	0	3.8497083741	5.8139072160	-0.0212227886
C	0	6.5744284494	4.0348240098	0.9969349471
H	0	6.5987692250	1.8950695369	1.3371775570
H	0	6.2247096736	6.1313577551	0.5959003310
H	0	7.6232016961	4.1760870368	1.2675664235
C	0	0.3059005325	3.0049043884	-0.6767820651
O	0	-0.3687320755	2.6054471729	-1.8227958556
H	0	-0.6088222396	1.6566574959	-1.6981979350
Cl	0	-0.9723718040	-0.2974918054	-1.0198828488
H	0	-1.1319522774	-2.4071415878	-0.4734842304
H	0	-2.9504458853	-0.8687162616	-0.6844961885
N	0	-1.4477956102	-3.2401899526	0.0579980996
N	0	-3.8885455246	-1.2845374849	-0.5329698765
C	0	-2.8092400835	-3.4193698805	0.1495574140
S	0	-0.4528151784	-3.5039291375	1.4133429257
C	0	-3.8858185341	-2.5626220385	-0.1339888962
C	0	-4.9541469232	-0.4122116418	-0.7639657154
C	0	-3.6771044572	-4.5683540389	0.5012330376
O	0	-1.1971722326	-4.3654972333	2.3265695905
O	0	0.8742182855	-3.8697478280	0.9300790417
C	0	-0.3489535898	-1.8563956396	2.1202381633
C	0	-4.8838923843	-3.6363347079	0.2348554269
C	0	-6.3010954539	-0.7848212759	-0.6415865360
C	0	-4.6274962342	0.9023020231	-1.1381219752
O	0	-3.5443570215	-5.7201270913	0.8320731071
C	0	0.8972434663	-1.2699805320	2.3316715314
C	0	-1.5355968341	-1.1887441570	2.4147765332
O	0	-6.0849619337	-3.7354479699	0.2860254016
H	0	-6.5692889850	-1.8040651620	-0.3611817408
C	0	-7.2969678856	0.1610265889	-0.8883653600
C	0	-5.6405980147	1.8288119230	-1.3774102636

H	0	-3.5788096559	1.1849652551	-1.2440336314
C	0	0.9453414743	0.0129734281	2.8800502135
H	0	1.8103416279	-1.8019101928	2.0720593759
C	0	-1.4681232881	0.1108439387	2.9165094547
H	0	-2.5024700862	-1.6606345755	2.2384945468
C	0	-6.9854743533	1.4716194515	-1.2580597035
C	0	-8.7404325101	-0.2338028344	-0.7006210171
C	0	-5.2659489403	3.2453976363	-1.7257447771
C	0	-0.2325029268	0.7065311918	3.1696432222
C	0	2.2687154612	0.6624322062	3.2040547950
C	0	-2.7541296909	0.8758489898	3.1058419491
H	0	-7.7733828003	2.1957081487	-1.4594853159
F	0	-9.1408262460	-0.0424677125	0.5724196227
F	0	-9.5635425968	0.4887296869	-1.4807351012
F	0	-8.9489415733	-1.5302161458	-0.9845788767
F	0	-6.2537763683	3.8937797489	-2.3599967207
F	0	-4.9752795037	3.9629168875	-0.6165520829
F	0	-4.1750210984	3.2970666357	-2.5133657601
H	0	-0.1896695664	1.7159471584	3.5761632998
F	0	3.2898813233	0.0887320565	2.5507185762
F	0	2.2644025954	1.9716596773	2.8891586351
F	0	2.5416287948	0.5804566861	4.5191596851
F	0	-3.5840570827	0.2613277941	3.9606243610
F	0	-2.5413682603	2.1202349009	3.5591530077
F	0	-3.4156768971	0.9868965480	1.9313847750
H	0	0.0301474975	3.0353321387	1.5080899798
H	0	-0.8435672419	1.7795651214	0.6179015108

TS1a

0 1

P	-1.7486309138	2.9359985042	0.1208419605
C	-1.8868115877	2.0524058023	1.7098568425
N	2.6239061008	0.4516403762	-0.1416598324

H	1.9815254465	0.3430580649	-0.9490163670
O	4.0553683559	-0.0376532011	2.8561614289
F	-3.0093558004	-2.4737118823	5.2198797883
Au	-1.7120085388	1.5176292185	-1.7125009998
N	-0.0385652563	-0.9172134624	0.8324325642
H	-0.0449245888	-0.6154886538	-0.1508656655
O	1.2878779307	-1.3796659515	3.9178692077
F	-3.6734743080	-4.2819140638	4.2369475154
C	-0.7851673019	1.9307097324	2.5693810686
H	0.1665664345	2.3985060317	2.3125651939
F	-1.5608196008	-3.9562959228	4.5982854228
C	-0.9059935672	1.2037720053	3.7568351938
H	-0.0447980904	1.0999582557	4.4186383825
F	-5.6990341880	-2.5379630940	0.0885879880
C	-2.1222809849	0.6045166248	4.0939895734
H	-2.2096408990	0.0233141407	5.0133177215
F	-5.5018705665	-0.4497556389	0.6144477748
C	-3.2217111792	0.7256525022	3.2386999248
H	-4.1681856555	0.2437114046	3.4894281057
F	-4.5524107935	-1.1991816463	-1.1778403495
C	-3.1038891224	1.4347492637	2.0442927517
H	-3.9603709333	1.5011966528	1.3707940312
C	-3.1323710275	4.1307680379	0.0884032409
C	-3.5230558014	4.6544196536	-1.1549286594
H	-3.0287675916	4.3126352344	-2.0677101680
C	-4.5361397531	5.6116003906	-1.2274605476
H	-4.8334865561	6.0142123258	-2.1981969235
C	-5.1720621183	6.0469677560	-0.0598783575
H	-5.9704013842	6.7902677144	-0.1171271054
C	-4.7875630395	5.5275002800	1.1794901342
H	-5.2818702768	5.8652835800	2.0929975621
C	-3.7687734740	4.5738767782	1.2580565282
H	-3.4762792654	4.1747549344	2.2303654312
C	-0.2417625850	3.9585515023	0.2532397200
C	-0.2317772614	5.0871492255	1.0905557742

H	-1.1287161001	5.3702486361	1.6444936984
C	0.9268583859	5.8541906420	1.2128218257
H	0.9301362045	6.7300156218	1.8654404241
C	2.0782757677	5.5037275604	0.4985707595
H	2.9841308528	6.1067296344	0.5938015221
C	2.0708746316	4.3855569062	-0.3381357832
H	2.9635065905	4.1043193199	-0.8958790222
C	0.9129221055	3.6121922041	-0.4636207146
C	2.2903435983	-0.0665352893	1.0651897474
C	2.9669069075	-0.2591729068	2.3751388500
C	1.6648673476	-0.9014145935	2.8735400273
C	1.1066328415	-0.6924714766	1.4956062013
C	-1.2385484718	-1.4622539578	1.3024874654
C	-1.3468710929	-2.1458778295	2.5181286848
H	-0.4820980056	-2.2564621944	3.1706587071
C	-2.5847403128	-2.6625613188	2.9031205458
C	-3.7214520707	-2.5034334730	2.1114571670
H	-4.6809805814	-2.9127668802	2.4249831336
C	-3.6009245114	-1.8096503346	0.9054801777
C	-2.3735522385	-1.3058377738	0.4942354440
H	-2.2934407424	-0.7735174606	-0.4523462694
C	-2.7039250944	-3.3506446423	4.2366812704
C	-4.8348437404	-1.5104001695	0.0990410348
Cl	0.3478717511	-0.0063696246	-2.2599223494
C	-2.9356653060	0.4513276614	-3.7757032130
C	-3.5388180901	1.3975247413	-3.2792981807
H	-4.2430785057	2.1685030563	-3.0111195610
H	0.9109324846	2.7387408974	-1.1186884634
S	4.0303554592	1.3290842906	-0.4860783038
O	3.7577329905	1.9300077933	-1.7923395271
O	4.3639545257	2.1291110742	0.6861832334
C	5.2910385357	0.0796383608	-0.6980129129
C	5.9817611138	-0.3788481399	0.4223945407
C	5.5453540248	-0.4013108558	-1.9818788900
C	6.9499361765	-1.3686648431	0.2408465402

H	5.7436269904	0.0109461661	1.4138894604
C	6.5253896530	-1.3829591925	-2.1400392620
H	4.9903513066	-0.0116561949	-2.8352804010
C	7.2260519550	-1.8706304518	-1.0330815724
H	7.9866693638	-2.6403690877	-1.1639093723
C	-2.3349527271	-0.7229543688	-4.4356246868
H	-1.4249164701	-0.4143730387	-4.9719424733
H	-3.0581087297	-1.1041454505	-5.1724903771
C	7.6636440391	-1.9312276075	1.4468377663
C	6.8671357931	-1.8850123301	-3.5223373065
F	5.8116667613	-1.8170425349	-4.3487519168
F	7.8534202175	-1.1572695607	-4.0773535375
F	7.2851307105	-3.1607270163	-3.4954518675
F	7.8402905851	-0.9988288102	2.3949589648
F	6.9608441672	-2.9348325465	2.0034196977
F	8.8707788405	-2.4241850507	1.1256936125
N	-2.0181029670	-1.7864325746	-3.5115041205
C	-2.9112258763	-2.7689018010	-3.2025809024
H	-1.2156308212	-1.6155050986	-2.9135292773
O	-3.9666630969	-2.9130181708	-3.8094259399
C	-2.5325215306	-3.6350850517	-2.0344856716
C	-1.2404288087	-3.6757720582	-1.4865307584
C	-3.5558813931	-4.3784659414	-1.4299304146
C	-0.9889936332	-4.4216026799	-0.3337045606
H	-0.4173099633	-3.1203752318	-1.9404904136
C	-3.3055221113	-5.1163021597	-0.2737047514
H	-4.5541271344	-4.3357336622	-1.8661124333
C	-2.0223213013	-5.1330882868	0.2821366803
H	0.0155096287	-4.4331580765	0.0945211792
H	-4.1171746993	-5.6667237403	0.2070107687
H	-1.8291954371	-5.6923515028	1.2002052217

TS1b

0 1

P	0	1.7807516860	1.3885798154	1.8474658101
C	0	1.7720676049	2.5728062856	0.4591914381
N	0	-2.7300754564	0.3488745998	0.7042732571
H	0	-2.1541101846	-0.5136491884	0.7172610644
O	0	-4.2265732639	3.1458229792	-0.3787674882
F	0	2.9075970151	3.8655389944	-4.7689453631
Au	0	1.3635972059	-0.7577520143	1.1371478033
N	0	-0.5637639617	0.5487162426	-1.5864366752
H	0	-0.5275037269	-0.3177587812	-1.0416600452
O	0	-1.8568529858	3.5419897547	-2.5578346263
F	0	1.8794544328	2.8825102576	-6.3950360194
C	0	0.7267593661	3.4899529133	0.2815384504
H	0	-0.1101423458	3.5130488502	0.9804659258
F	0	0.7569142189	4.0412395594	-4.9480491729
C	0	0.7624879139	4.3889321188	-0.7891858685
H	0	-0.0582970050	5.0940226573	-0.9288759693
F	0	4.4213042046	-1.0688969043	-4.5606654851
C	0	1.8375869027	4.3796806227	-1.6793884218
H	0	1.8605684977	5.0798374683	-2.5160411759
F	0	4.1911129357	-1.3650412839	-2.4283146020
C	0	2.8721525129	3.4519212498	-1.5161607142
H	0	3.6991359644	3.4259472750	-2.2272579773
F	0	2.9556686688	-2.4764881660	-3.8119068974
C	0	2.8362533326	2.5448919680	-0.4589251096
H	0	3.6444421928	1.8230679840	-0.3350759142
C	0	3.3546278375	1.6678095825	2.7324541947
C	0	3.7597054054	0.6993002099	3.6632906378
H	0	3.1979766014	-0.2328428405	3.7587447688
C	0	4.8812855124	0.9236397778	4.4602741150
H	0	5.1941903630	0.1641974665	5.1789722686
C	0	5.6141501061	2.1067040031	4.3240625174
H	0	6.4979656989	2.2758898042	4.9431846026

C	0	5.2234349179	3.0651807461	3.3859221651
H	0	5.7974126210	3.9873751521	3.2709364189
C	0	4.0930673312	2.8520334711	2.5923537807
H	0	3.7904929160	3.6094475266	1.8680731422
C	0	0.5266084955	1.9867951050	3.0348578735
C	0	0.6968745514	3.2408781958	3.6480390370
H	0	1.5630543396	3.8594834063	3.4055471609
C	0	-0.2404302831	3.6982826574	4.5732491356
H	0	-0.1055620613	4.6749170153	5.0433355753
C	0	-1.3461718723	2.9049134743	4.9021016239
H	0	-2.0774033433	3.2631351363	5.6302556972
C	0	-1.5156034150	1.6572083334	4.2998926483
H	0	-2.3779103406	1.0365361387	4.5422627428
C	0	-0.5827141989	1.1973014408	3.3654369811
C	0	-2.5317739250	1.2923843186	-0.2462816684
C	0	-3.2221040214	2.5381757489	-0.6730041185
C	0	-2.1207530536	2.7071964195	-1.7245765942
C	0	-1.5602984236	1.3914562648	-1.2598868540
C	0	0.4602761128	0.7071689764	-2.5321244968
C	0	0.5620455789	1.8431224532	-3.3506819046
H	0	-0.1755361070	2.6418789462	-3.2881064520
C	0	1.6290422894	1.9528860473	-4.2375014402
C	0	2.6064125780	0.9575004265	-4.3370942043
H	0	3.4425117086	1.0593127386	-5.0278682195
C	0	2.4813150613	-0.1748902811	-3.5355955299
C	0	1.4147953639	-0.3137621756	-2.6440629509
H	0	1.3228872691	-1.2351806709	-2.0669985180
C	0	1.7810931395	3.1868252312	-5.0882701862
C	0	3.5143651603	-1.2716534948	-3.5917863239
Cl	0	-0.7653201771	-2.0944932090	0.5572121025
C	0	2.7082206944	-2.8999158004	0.1437015728
C	0	3.6034821913	-2.2530741750	0.6744390337
H	0	-0.7214010605	0.2217201456	2.8964311659
S	0	-3.8955649767	0.4179631918	1.9336372039
O	0	-3.4708659045	-0.6102108493	2.8837713820

O	0	-4.0658255025	1.8136921766	2.3165313513
C	0	-5.3968682488	-0.1386694661	1.1393882449
C	0	-6.1689541042	0.7848076598	0.4375617415
C	0	-5.7480518069	-1.4846936696	1.2410935795
C	0	-7.3275805352	0.3285569300	-0.1965045119
H	0	-5.8533000292	1.8286538239	0.3707286644
C	0	-6.9144121618	-1.9147491131	0.6078034399
H	0	-5.1223427099	-2.1755838139	1.8055666493
C	0	-7.7040294283	-1.0133670243	-0.1136084291
H	0	-8.6115388560	-1.3565854308	-0.6106330518
C	0	-8.1441914247	1.2996249940	-1.0154336015
C	0	-7.3584619615	-3.3520508958	0.7351260567
F	0	-6.3352924286	-4.1646568552	1.0396724434
F	0	-8.2829334367	-3.4943392889	1.7023512113
F	0	-7.9109454358	-3.7982028005	-0.4051448411
F	0	-8.1978737466	2.5081693694	-0.4343748636
F	0	-7.6108866634	1.4724146283	-2.2386988047
F	0	-9.4046095726	0.8711592032	-1.1882612244
C	0	4.7389605525	-1.5733743097	1.2158600521
C	0	5.2322286741	-0.4113354693	0.5912100640
C	0	5.4067317766	-2.0904590242	2.3429430745
C	0	6.3629765068	0.2267138415	1.0941656613
H	0	4.7245618965	-0.0335374195	-0.2962310939
C	0	6.5487027423	-1.4553947276	2.8276032649
H	0	5.0240754262	-2.9910413869	2.8260831752
C	0	7.0245633729	-0.2943578397	2.2107683037
H	0	6.7310877656	1.1350789233	0.6130335547
H	0	7.0638713930	-1.8612990048	3.7008305747
H	0	7.9087024691	0.2104867323	2.6052552678
C	0	2.0147009598	-3.9193401562	-0.6658869897
C	0	3.0506632325	-4.6379430959	-1.5903561013
C	0	1.3896700616	-5.0273747320	0.2256781265
C	0	2.4113254915	-5.6709503034	-2.4870749765
C	0	0.7241383742	-6.1091397163	-0.6291401581
H	0	2.1893484526	-5.4614456571	0.8486466402

H	0	0.6643425253	-4.5545346712	0.9045563342
C	0	1.7087881321	-6.7371023490	-1.6189560590
H	0	3.1921693311	-6.1128292876	-3.1213545345
H	0	1.6708960061	-5.1630738188	-3.1232423424
H	0	0.3035072981	-6.8851069855	0.0296637562
H	0	-0.1210401005	-5.6648206260	-1.1781947229
H	0	1.1955079659	-7.4600999495	-2.2719156032
H	0	2.4774221792	-7.3011334727	-1.0627588606
O	0	1.0471377400	-3.3613431772	-1.5375702540
H	0	0.2961015534	-3.0607836378	-0.9854558997
O	0	4.2397428676	-4.4457724190	-1.5141286187

TS2a

0 1

P	-2.0340943133	3.0593229580	0.6532662811
C	-1.4371578909	1.7213810297	1.7514203274
N	2.4958567830	-0.2620667799	0.6935012550
H	1.8672742571	0.2227484646	0.0091384219
O	3.9244608609	-2.7671690093	2.3637389369
F	-2.2225795706	-5.9234440600	2.7513073279
Au	-2.7900331954	2.1533825401	-1.3580174971
N	-0.2100144481	-1.8894145521	0.4168421815
H	-0.1912188822	-0.9881096812	-0.0937911493
O	1.1445480296	-4.4615207579	2.1710710747
F	-4.3466333683	-5.8153125643	2.3313710769
C	-0.1185458525	1.6585596712	2.2205644514
H	0.6137372590	2.4116656532	1.9288149134
F	-2.9876000240	-6.6141469790	0.8502048336
C	0.2720209661	0.6075809170	3.0568592126
H	1.3019415338	0.5588067852	3.4140505231
F	-6.1834519152	-2.0427911487	-0.0051848871
C	-0.6436338435	-0.3833489016	3.4194784921
H	-0.3299937099	-1.2078267344	4.0636575861

F	-4.9369574505	-0.2785994154	-0.1779481191
C	-1.9574787648	-0.3277090935	2.9424095614
H	-2.6719824979	-1.1103271546	3.2056423261
F	-5.0630237141	-1.6815336814	-1.8199868759
C	-2.3536169200	0.7177691449	2.1086547771
H	-3.3759629922	0.7455519252	1.7249216433
C	-3.3742803823	3.8592972111	1.5998460318
C	-4.4302094930	4.4717773584	0.9078687891
H	-4.4611617372	4.4283321283	-0.1836186468
C	-5.4420015431	5.1284135400	1.6124466445
H	-6.2625322725	5.6015279228	1.0686288752
C	-5.4073699652	5.1710650839	3.0096250931
H	-6.2020724415	5.6794995706	3.5603471400
C	-4.3587381898	4.5574802806	3.7033085337
H	-4.3315767028	4.5872281348	4.7948209407
C	-3.3432458934	3.9031234841	3.0031436342
H	-2.5270747686	3.4244838558	3.5475687018
C	-0.6889700225	4.2919583257	0.5553216757
C	-0.7467565199	5.4952887276	1.2757303922
H	-1.6116708839	5.7245720503	1.8992364434
C	0.3091276717	6.4071145865	1.1954595752
H	0.2577689896	7.3424634237	1.7573866824
C	1.4258347466	6.1227593155	0.4041056762
H	2.2517871468	6.8356826341	0.3480857130
C	1.4829125601	4.9250245595	-0.3155470789
H	2.3555395063	4.6941503790	-0.9304649388
C	0.4278814315	4.0135058558	-0.2509531093
C	2.1438848768	-1.4954644678	1.1387018142
C	2.8248410491	-2.5728257174	1.8920988217
C	1.5183111262	-3.3751371938	1.7943753429
C	0.9452803283	-2.2234689276	1.0123379129
C	-1.4559111740	-2.5119710284	0.4955250005
C	-1.6524491945	-3.7556297808	1.1130136352
H	-0.8078553033	-4.3049148863	1.5289920162
C	-2.9406767875	-4.2796179390	1.2073395943

C	-4.0477320691	-3.5901319543	0.7096495173
H	-5.0508271129	-4.0012392341	0.8072318779
C	-3.8393503292	-2.3571085910	0.0892565702
C	-2.5578376837	-1.8229846452	-0.0368754875
H	-2.4060949087	-0.8540381533	-0.5146017577
C	-3.1252706600	-5.6542666896	1.7950572573
C	-5.0096064480	-1.5952271685	-0.4720544291
Cl	0.3954906627	0.6449110054	-1.3075558722
C	-2.6340339867	0.4149874789	-3.6201879817
C	-3.5596197586	0.9303031181	-2.9029864573
H	-4.5545930115	0.5079843974	-2.7617845081
H	0.4824074587	3.0729517662	-0.8031845941
S	3.5913111078	0.7537712382	1.4781934998
O	3.1533788667	2.1115350452	1.1524562405
O	3.7187277045	0.2955660724	2.8613513505
C	5.1477850803	0.4616375066	0.6480872126
C	5.8675339835	-0.6959578144	0.9451831568
C	5.6011953207	1.4084398860	-0.2674525012
C	7.0777727982	-0.9060813031	0.2816690034
H	5.4746491017	-1.4240527516	1.6606549554
C	6.8211100283	1.1821932704	-0.9096447004
H	5.0107392854	2.3022644301	-0.4680107062
C	7.5595723618	0.0276389914	-0.6405083997
H	8.5085304247	-0.1451160843	-1.1477660031
C	-1.2467234960	0.3804882509	-4.1180225683
H	-0.6577786456	1.1746580400	-3.6413268187
H	-1.2210036690	0.5043491277	-5.2132434565
C	7.8430792992	-2.1829426136	0.5342887475
C	7.3639684430	2.2215309367	-1.8600074441
F	6.3768345215	2.8614658261	-2.5080319183
F	8.0778523688	3.1568119622	-1.2066677804
F	8.1741360107	1.6781166860	-2.7821998336
F	7.7341318193	-2.5797300164	1.8112591742
F	7.3800955792	-3.1876367298	-0.2331099388
F	9.1499809403	-2.0445015979	0.2579202470

N	-0.7168446504	-0.9159918669	-3.7197169465
C	-1.6946820911	-1.8350230059	-3.6301216388
H	0.0472083088	-0.8900175755	-3.0374937145
O	-2.8415320282	-1.4682436191	-4.0113431222
C	-1.4471212527	-3.1503769619	-3.0172572857
C	-0.1474288747	-3.5752700831	-2.6922750583
C	-2.5524006903	-3.9479190557	-2.6743895924
C	0.0392331260	-4.7743283775	-2.0063124789
H	0.7191938840	-2.9741159024	-2.9725391840
C	-2.3583147485	-5.1482132311	-1.9968813308
H	-3.5542580260	-3.5905296281	-2.9113902537
C	-1.0649704593	-5.5572082811	-1.6536192433
H	1.0475206658	-5.0969214669	-1.7398132266
H	-3.2156740715	-5.7553687328	-1.7040246239
H	-0.9207889742	-6.4845399633	-1.0958965348

TS2b

0 1

C	0	-0.6590291762	2.9346563308	0.8491607023
C	0	-1.7692928572	4.0057941261	1.0113327341
C	0	-1.9222383630	4.9261813235	-0.2068980889
C	0	-0.5867748867	5.5939502757	-0.5675910995
H	0	-1.5434020972	4.6245928885	1.8955199816
H	0	-2.7296828630	3.5124645241	1.2126296313
H	0	-2.2741944021	4.3453732384	-1.0658943628
H	0	-2.6750689690	5.7018358503	-0.0061073122
H	0	-0.5952384975	5.9736686817	-1.6051379927
H	0	-0.3479994191	6.4580653506	0.0735120504
C	0	0.5476472833	4.6307394990	-0.4674837886
O	0	1.7211430228	4.9682012546	-0.3292538717
C	0	2.6036664786	3.2575613172	-0.1871612122
C	0	1.5789170307	2.4760306620	-0.2861779082
Au	0	2.1569518913	0.4753727750	-0.5377250659

P	0	3.5054167221	-1.3775115234	-0.9627775997
C	0	2.7430952841	-2.8898355494	-1.6238049539
C	0	1.4833246673	-2.7843496574	-2.2316313088
C	0	3.3738832191	-4.1418289964	-1.5366320013
C	0	0.8578954588	-3.9233939175	-2.7421282585
H	0	0.9749680289	-1.8185016107	-2.2747587057
C	0	2.7415721479	-5.2766233689	-2.0463346318
H	0	4.3481598864	-4.2367740700	-1.0536815056
C	0	1.4832256481	-5.1690446102	-2.6477013784
H	0	-0.1299353166	-3.8380058435	-3.1996914889
H	0	3.2305679786	-6.2501969164	-1.9679928906
H	0	0.9864464220	-6.0611351490	-3.0360292329
C	0	4.8060878945	-0.8640718156	-2.1533468658
C	0	5.4530622740	-1.7906765143	-2.9848334759
C	0	5.1931219770	0.4863738345	-2.1932721805
C	0	6.4795482298	-1.3711498504	-3.8348092678
H	0	5.1551505668	-2.8400565192	-2.9774718886
C	0	6.2274557986	0.8996063741	-3.0357491293
H	0	4.6858489464	1.2174790751	-1.5617432577
C	0	6.8721600012	-0.0287815378	-3.8583304717
H	0	6.9747144179	-2.0983104186	-4.4823355051
H	0	6.5216182787	1.9514093443	-3.0489910623
H	0	7.6766503127	0.2938092865	-4.5232736613
C	0	4.4364137803	-1.8492427249	0.5442266975
C	0	3.9553155918	-2.8518313660	1.4012217228
C	0	5.5615529899	-1.0978104214	0.9188039065
C	0	4.5976541807	-3.0956805301	2.6184520612
H	0	3.0755005196	-3.4347804450	1.1278307684
C	0	6.1968458991	-1.3471099571	2.1364358066
H	0	5.9393906554	-0.3089099824	0.2665534803
C	0	5.7159742019	-2.3446998226	2.9905253354
H	0	4.2159140918	-3.8772565871	3.2794860990
H	0	7.0719841261	-0.7572432702	2.4190257764
H	0	6.2131381799	-2.5362949039	3.9441489207
C	0	4.0141120039	3.4769584465	-0.0378092961

C	0	4.7228122490	2.7236748314	0.9220570218
C	0	4.7138374460	4.3470324783	-0.8962368093
C	0	6.1117253173	2.8153853900	0.9900387112
H	0	4.1777432158	2.0542768401	1.5860339623
C	0	6.1012491574	4.4449764998	-0.8063425649
H	0	4.1615011224	4.9329065703	-1.6323133851
C	0	6.8030138667	3.6735604607	0.1280623455
H	0	6.6558698873	2.2155764465	1.7225681594
H	0	6.6409699438	5.1182181484	-1.4756210555
H	0	7.8914120949	3.7443371765	0.1854705443
C	0	0.2270378285	3.1326216162	-0.4102372692
O	0	-0.4464417107	2.7950534005	-1.5957634831
H	0	-0.6788684814	1.8406159688	-1.5272096647
Cl	0	-0.8709913755	-0.2152792832	-1.0000011881
H	0	-0.9974673163	-2.3516916646	-0.6162911416
H	0	-2.8653661030	-0.8340664269	-0.7742989661
N	0	-1.3210205719	-3.2364600150	-0.1811796208
N	0	-3.7951458631	-1.2876945496	-0.7086124339
C	0	-2.6806538880	-3.4546726726	-0.2007429750
S	0	-0.4321072475	-3.5709403678	1.2327762458
C	0	-3.7680027503	-2.6018330590	-0.4517779300
C	0	-4.8809479259	-0.4244349586	-0.8789473262
C	0	-3.5245724197	-4.6582620107	-0.0226039496
O	0	-1.2259478158	-4.5080483668	2.0219667759
O	0	0.9398135902	-3.8746663435	0.8400592029
C	0	-0.4311059757	-1.9713821200	2.0471019113
C	0	-4.7477024774	-3.7394646277	-0.2590752412
C	0	-6.2175705714	-0.8527489137	-0.8571737187
C	0	-4.5905691833	0.9352729395	-1.0746859043
O	0	-3.3677098011	-5.8338853719	0.1944131194
C	0	0.7773465422	-1.3433469008	2.3391222997
C	0	-1.6580285796	-1.3748780246	2.3301938350
O	0	-5.9451682621	-3.8788192281	-0.2869301445
H	0	-6.4586889331	-1.9063283064	-0.7144070201
C	0	-7.2380360804	0.0826026006	-1.0236138322

C	0	-5.6295228057	1.8517948177	-1.2345022586
H	0	-3.5509821872	1.2656558382	-1.1032026237
C	0	0.7453976712	-0.0884668716	2.9511386373
H	0	1.7227886976	-1.8239319689	2.0958699150
C	0	-1.6697687523	-0.1021225556	2.8992108824
H	0	-2.5954043695	-1.8819577880	2.1008939249
C	0	-6.9624738992	1.4397819749	-1.2132038077
C	0	-8.6745690434	-0.3694824903	-0.9448595696
C	0	-5.2883243394	3.3101178536	-1.3930096653
C	0	-0.4731836769	0.5367428547	3.2273484427
C	0	2.0251753891	0.5986299758	3.3603175181
C	0	-2.9908945821	0.6063342014	3.0618549778
H	0	-7.7684147811	2.1590911786	-1.3511956235
F	0	-9.1751642936	-0.1997228025	0.2954675705
F	0	-9.4638299703	0.3302901954	-1.7798646793
F	0	-8.8148124262	-1.6694166042	-1.2502618049
F	0	-6.3173791410	4.0288785511	-1.8649944055
F	0	-4.9302310943	3.8626653289	-0.2127481407
F	0	-4.2504246455	3.4902154681	-2.2327370873
H	0	-0.4956135420	1.5226001748	3.6902367372
F	0	3.1032328201	0.0723452780	2.7583924645
F	0	1.9995750470	1.9133934922	3.0584960511
F	0	2.2247786115	0.5097363671	4.6866382043
F	0	-3.9449939359	-0.2146345064	3.5231451000
F	0	-2.9073551211	1.6559230972	3.8899844439
F	0	-3.4261703566	1.0767931123	1.8688827496
H	0	-0.0025008357	2.9206056401	1.7286331627
H	0	-1.0790366056	1.9252274521	0.7449894205

TS3b

0 1

C	0	0.5428840000	-3.1221010000	0.4974920000
C	0	1.8326400000	-3.9389620000	0.4510590000

C	0	1.9361080000	-4.7700810000	-0.8381120000
C	0	0.5600950000	-5.3941580000	-1.1032400000
H	0	1.8407900000	-4.6150730000	1.3203900000
H	0	2.7043730000	-3.2782200000	0.5429230000
H	0	2.2098240000	-4.1201650000	-1.6772220000
H	0	2.7104700000	-5.5443530000	-0.7558020000
H	0	0.4757060000	-5.7403550000	-2.1475070000
H	0	0.3546930000	-6.2541550000	-0.4482520000
C	0	-0.4676020000	-4.3585600000	-0.8543690000
O	0	-1.6315440000	-4.6691700000	-0.3158330000
C	0	-2.4235610000	-3.4935680000	-0.2382240000
C	0	-1.7437950000	-2.4142190000	-0.7183030000
Au	0	-2.3894320000	-0.4683800000	-0.9220350000
P	0	-3.5163340000	1.5778720000	-1.1406830000
C	0	-2.5513000000	3.0898930000	-1.4408820000
C	0	-1.2711250000	2.9549370000	-1.9983680000
C	0	-3.0512350000	4.3690390000	-1.1460720000
C	0	-0.4996530000	4.0892480000	-2.2584510000
H	0	-0.8622720000	1.9619870000	-2.1945680000
C	0	-2.2728400000	5.4978860000	-1.4035320000
H	0	-4.0386260000	4.4860170000	-0.6960130000
C	0	-0.9969850000	5.3597600000	-1.9594450000
H	0	0.5015740000	3.9767010000	-2.6795710000
H	0	-2.6615480000	6.4897520000	-1.1623760000
H	0	-0.3862320000	6.2450680000	-2.1505880000
C	0	-4.7730600000	1.4774240000	-2.4740380000
C	0	-5.2971680000	2.6264480000	-3.0862570000
C	0	-5.2438280000	0.2125630000	-2.8641600000
C	0	-6.2843060000	2.5098950000	-4.0671720000
H	0	-4.9313040000	3.6152880000	-2.8070130000
C	0	-6.2374500000	0.1006840000	-3.8401740000
H	0	-4.8278830000	-0.6867940000	-2.4037440000
C	0	-6.7587820000	1.2487650000	-4.4430180000
H	0	-6.6831620000	3.4094800000	-4.5414010000
H	0	-6.5982400000	-0.8874820000	-4.1341370000

H	0	-7.5308000000	1.1614810000	-5.2109800000
C	0	-4.4886270000	1.8506420000	0.3898050000
C	0	-3.9690640000	2.6149780000	1.4461950000
C	0	-5.6935570000	1.1519010000	0.5674460000
C	0	-4.6486900000	2.6717460000	2.6662670000
H	0	-3.0326660000	3.1604320000	1.3246740000
C	0	-6.3655300000	1.2117710000	1.7899500000
H	0	-6.1078920000	0.5540740000	-0.2466730000
C	0	-5.8430740000	1.9683750000	2.8438730000
H	0	-4.2355730000	3.2682490000	3.4830680000
H	0	-7.3024180000	0.6647210000	1.9187700000
H	0	-6.3684700000	2.0112820000	3.8005770000
C	0	-3.7478430000	-3.7087210000	0.3443990000
C	0	-4.6169340000	-2.6277540000	0.5891020000
C	0	-4.1802370000	-5.0078140000	0.6750500000
C	0	-5.8773580000	-2.8400800000	1.1414660000
H	0	-4.2941880000	-1.6141550000	0.3526260000
C	0	-5.4446200000	-5.2154910000	1.2287970000
H	0	-3.5203620000	-5.8569920000	0.4966870000
C	0	-6.2993550000	-4.1349060000	1.4645660000
H	0	-6.5300980000	-1.9845820000	1.3287560000
H	0	-5.7626390000	-6.2303560000	1.4785480000
H	0	-7.2872960000	-4.2994300000	1.9006720000
C	0	-0.4307130000	-2.9527580000	-1.1193930000
O	0	0.3965730000	-2.4160730000	-2.0240770000
H	0	0.5136320000	-1.4496310000	-1.8198890000
Cl	0	0.9030990000	0.2999430000	-0.8480500000
H	0	1.0503180000	2.4311670000	-0.2561410000
H	0	2.9003220000	0.9206290000	-0.5914910000
N	0	1.3768310000	3.2303400000	0.3155010000
N	0	3.8328310000	1.3535190000	-0.4609270000
C	0	2.7372710000	3.4288280000	0.3648800000
S	0	0.4400920000	3.3765590000	1.7300750000
C	0	3.8187670000	2.6102730000	0.0014280000
C	0	4.9106010000	0.5173030000	-0.7624610000

C	0	3.5931810000	4.5745550000	0.7515250000
O	0	1.2151660000	4.1795630000	2.6705120000
O	0	-0.9122470000	3.7544520000	1.3343130000
C	0	0.3931130000	1.6766830000	2.3041960000
C	0	4.8085760000	3.6875030000	0.3878200000
C	0	6.2513260000	0.9188760000	-0.6653790000
C	0	4.6049260000	-0.7885880000	-1.1795890000
O	0	3.4478330000	5.7011270000	1.1553810000
C	0	-0.8320330000	1.0286320000	2.4382550000
C	0	1.6011480000	1.0263740000	2.5477060000
O	0	6.0079150000	3.8133420000	0.3968770000
H	0	6.5037660000	1.9326710000	-0.3527100000
C	0	7.2620710000	0.0089790000	-0.9770910000
C	0	5.6329840000	-1.6798300000	-1.4824370000
H	0	3.5607980000	-1.0940940000	-1.2654190000
C	0	-0.8397410000	-0.3043360000	2.8522660000
H	0	-1.7608820000	1.5517850000	2.2203170000
C	0	1.5756300000	-0.3208660000	2.9072550000
H	0	2.5518520000	1.5495580000	2.4402940000
C	0	6.9715670000	-1.2944460000	-1.3871580000
C	0	8.6998580000	0.4343560000	-0.8143030000
C	0	5.2776900000	-3.0892920000	-1.8757020000
C	0	0.3597200000	-0.9842080000	3.0791430000
C	0	-2.1479410000	-1.0126260000	3.1060970000
C	0	2.8838770000	-1.0622700000	3.0221280000
H	0	7.7700780000	-1.9912720000	-1.6365360000
F	0	9.1343850000	0.2266510000	0.4448400000
F	0	9.5214310000	-0.2510090000	-1.6285790000
F	0	8.8701540000	1.7413470000	-1.0750060000
F	0	6.2871530000	-3.7190680000	-2.4936210000
F	0	4.9532680000	-3.8337050000	-0.7940690000
F	0	4.2126500000	-3.1249130000	-2.6997530000
H	0	0.3503560000	-2.0298100000	3.3840680000
F	0	-3.1658450000	-0.4457470000	2.4430770000
F	0	-2.0864840000	-2.3074730000	2.7403170000

F	0	-2.4657330000	-0.9935300000	4.4134600000
F	0	3.7687440000	-0.4030280000	3.7826090000
F	0	2.7287620000	-2.2912920000	3.5345080000
F	0	3.4526600000	-1.2107700000	1.8024720000
H	0	-0.1554730000	-3.3620740000	1.3042060000
H	0	0.7018400000	-2.0376350000	0.4513800000

11. References

[1] Wipf, P.; Aoyama, Y.; Benedum, T. E. A Practical Method for Oxazole Synthesis by Cycloisomerization of Propargyl Amides. *Org. Lett.* **2004,** *6*, 3593-3595.

[2] Bunnelle, E. M.; Smith, C. R.; Lee, S. K.; Singaram, S. W.; Rhodes, A. J.; Sarpong, R. Pt-catalyzed cyclization/migration of propargylic alcohols for the synthesis of 3(2*H*)-furanones, pyrrolones, indolizines, and indolizinones. *Tetrahedron* **2008**, *64*, 7008-7014.

[3] Zhang, Z.; Smal, V.; Retailleau, P.; Voituriez, A.; Frison, G.; Marinetti, A.; Guinchard, X. Tethered Counterion-Directed Catalysis: Merging the Chiral Ion-Pairing and Bifunctional Ligand Strategies in Enantioselective Gold(I) Catalysis. *J. Am. Chem. Soc.* **2020**, *142*, 3797-3805.

[4] Heard, A. W.; Goldup, S. M.; Synthesis of a Mechanically Planar Chiral Rotaxane Ligand for Enantioselective Catalysis. *Chem* **2020**, *6*, 994-1006.

[5] Kim, H. Y.; Oh, K. Highly Diastereo- and Enantioselective Aldol Reaction of Methyl α-Isocyanoacetate: A Cooperative Catalysis Approach. *Org. Lett.* **2011**, *13*, 1306-1309.

[6] Rostami, A.; Colin, A.; Li, X. Y.; Chudzinski, M. G.; Lough, A. J.; Taylor, M. S. N,N′-Diarylsquaramides: General, High-Yielding Synthesis and Applications in Colorimetric Anion Sensing. *J. Org. Chem.* **2010**, *75*, 3983-3992.

[7] Mauleón, P.; Zeldin, R. M.; González, A. Z.; Toste, F. D. Ligand-Controlled Access to [4+2] and [4+3] Cycloadditions in Gold-Catalyzed Reactions of Allene-Dienes. *J. Am. Chem. Soc.* **2009**, *131*, 6348-6349. *Chemtracts* **2010**, *23* (3), 99-102.

[8] Ebule, R. E.; Malhotra, D.; Hammond, G. B.; Xu, B. Ligand Effects in the Gold Catalyzed Hydration of Alkynes. *Adv. Synth. Catal.* **2016**, *358*, 1478-1481.

[9] González, A. Z.; Toste, F. D. Gold(I) Catalyzed Enantioselective [4+2]-Cycloaddition of Allene-dienes. *Org. Lett.* **2010**, *12*, 200-203.

[10] Collado, A.; Gómez-Suárez, A.; Martin, A. R.; Slawin, A. M. Z.; Nolan, S. P. Straightforward Synthesis of [Au(NHC)X] (NHC=N-Heterocyclic Carbene, X = Cl, Br, I) Complexes. *Chem. Commun.* **2013**, *49*, 5541-5543.

[11] Iglesias-Sigüenza, J.; Izquierdo, C.; Díez, E.; Fernández, R.; Lassaletta, J. M. N-Heterotricyclic cationic carbene ligands. Synthesis, reactivity and coordination chemistry. *Dalton Trans.* **2018**, *47*, 5196-5206.

[12] Lu, M.; Lu, Q.-B.; Honek, J. F. Squarate-based carbocyclic nucleosides: Syntheses, computational analyses and anticancer/antiviral evaluation. *Bioorg. Med. Chem. Lett.* **2017**, *27*, 282-287.

[13] Matador, E.; Retamosa, M. G.; Monge, D.; Iglesias-Sigüenza, J.; Fernández, R.; Lassaletta, J. M. Bifunctional Squaramide Organocatalysts for the Asymmetric Addition of Formaldehyde *tert*-Butylhydrazone to Simple Aldehydes. *Chem. Eur. J.* **2018**, *24*, 6854-6860.

[14] Hashmi, A. S. K.; Weyrauch, J. P.; Frey, W.; Bats, J. W. Gold Catalysis: Mild Conditions for the Synthesis of Oxazoles from N-Propargylcarboxamides and Mechanistic Aspects. *Org. Lett.* **2004**, *6*, 4391 – 4394.

[15] Binder, J. T.; Crone, B.; Kirsch, S. F.; Liébert, C.; Menz, H. Synthesis of Heterocyclic Systems by Transition-Metal-Catalyzed Cyclization-Migration Reactions – A Diversity-Oriented Strategy for the Construction of Spirocyclic 3(2*H*)-Furanones and 3-Pyrrolones. *Eur. J. Org. Chem.* **2007**, 1636-1647.

[16] Zhang, Z.; Smal, V.; Retailleau, P.; Voituriez, A.; Frison, G.; Marinetti, A.; Guinchard, X. Tethered Counterion-Directed Catalysis: Merging the Chiral Ion-Pairing and Bifunctional Ligand Strategies in Enantioselective Gold(I) Catalysis. *J. Am. Chem. Soc*. **2020**, *142*, 3797–3805.

[17] Johansson, M. J.; Gorin, D. J.; Staben, S. T.; Toste, F. D. Gold(I)-Catalyzed Stereoselective Olefin Cyclopropanation. *J. Am. Chem. Soc*. **2005**, *127*, 18002–18003.

[18] Frisch, M. J.; Trucks, G. W.; Schlegel, H. B.; Scuseria, G. E.; Robb, M. A.; Cheeseman, J. R.; Scalmani, G.; Barone, V.; Petersson, G. A.; Nakatsuji, H.; Li, X.; Caricato, M.; Marenich, A. V.; Bloino, J.; Janesko, B. G.; Gomperts, R.; Mennucci, B.; Hratchian, H. P.; Ortiz, J. V.; Izmaylov, A. F.; Sonnenberg, J. L.; Williams; Ding, F.; Lipparini, F.; Egidi, F.; Goings, J.; Peng, B.; Petrone, A.; Henderson, T.; Ranasinghe, D.; Zakrzewski, V. G.; Gao, J.; Rega, N.; Zheng, G.; Liang, W.; Hada, M.; Ehara, M.; Toyota, K.; Fukuda, R.; Hasegawa, J.; Ishida, M.; Nakajima, T.; Honda, Y.; Kitao, O.; Nakai, H.; Vreven, T.; Throssell, K.; Montgomery Jr., J. A.; Peralta, J. E.; Ogliaro, F.; Bearpark, M. J.; Heyd, J. J.; Brothers, E. N.; Kudin, K. N.; Staroverov, V. N.; Keith, T. A.; Kobayashi, R.; Normand, J.; Raghavachari, K.; Rendell, A. P.; Burant, J. C.; Iyengar, S. S.; Tomasi, J.; Cossi, M.; Millam, J. M.; Klene, M.; Adamo, C.; Cammi, R.; Ochterski, J. W.; Martin, R. L.; Morokuma, K.; Farkas, O.; Foresman, J. B.; Fox, D. J. Wallingford, CT, **2016**.

[19] Chai, J.-D.; Head-Gordon, M. Long-range corrected hybrid density functionals with damped atom–atom dispersion corrections. *Phys. Chem. Chem. Phys.* **2008**, *10*, 6615-6620.

[20] (a) Weigend, F.; Ahlrichs, R. Balanced basis sets of split valence, triple zeta valence and quadruple zeta valence quality for H to Rn: Design and assessment of accuracy. *Phys. Chem. Chem. Phys.* **2005**, *7*, 3297-3305. (b) Weigend, F. Accurate Coulomb-fitting basis sets for H to Rn. *Phys. Chem. Chem. Phys.* **2006**, *8*, 227-236.

[21] Marenich, A. V.; Cramer, C. J.; Truhlar, D. G. Universal Solvation Model Based on Solute Electron Density and on a Continuum Model of the Solvent Defined by the Bulk Dielectric Constant and Atomic Surface Tensions. *J. Phys. Chem. B.* **2009**, *113*, 6378-6396.

[22] Schlegel, H. B. Optimization of equilibrium geometries and transition structures. *J. Comput. Chem.* **1982**, *3*, 214-218.

[23] (a) González, C.; Schlegel, H. B. Reaction path following in mass-weighted internal coordinates. *J. Phys. Chem.* **1990**, *94*, 5523-5527. (b) González, C.; Schlegel, H. B. Improved algorithms for reaction path following: Higher-order implicit algorithms. *J. Chem. Phys.* **1991**, *95*, 5853-5860. (c) Hratchian, H. P.; Schlegel, H. B. Following Reaction Pathways Using a Damped Classical Trajectory Algorithm. *J. Phys. Chem. A* **2002**, *106*, 165-169.

[24] Tanaka, R.; Yamashita, M.; Chung, L. W.; Morokuma, K.; Nozaki, K. Mechanistic Studies on the Reversible Hydrogenation of Carbon Dioxide Catalyzed by an Ir-PNP Complex. *Organometallics* **2011**, *30*, 6742-6750.

[25] Legault, C. Y. CYLview, 1.0b. *Université de Sherbrooke* **2009**, http://www.cylview.org (visited Dec 1st, 2021).

[26] (a) Savin, A.; Becke, A. D.; Flad, J.; Nesper, R.; Preuss, H.; von Schnering, H. G. A New Look at Electron Localization. *Angew. Chem. Int. Ed.* **1991**, *30*, 409-412. (b) Savin, A.; Nesper, R.; Wengert, S.; Fässler, T. F. ELF: The Electron Localization Function. *Angew. Chem. Int. Ed.* **1997**, *36*, 1808-1832.

[27] (a) Silvi, B.; Savin, A. Classification of chemical bonds based on topological analysis of electron localization functions. *Nature* **1994**, *371*, 683-686. (b) Silvi, B.; Fourre, I.; Alikhani, M. E. The Topological Analysis of the Electron Localization Function. A Key for a Position Space Representation of Chemical Bonds. *Monats. Chem.* **2005**, *136*, 855-879.

[28] Noury, S.; Krokidis, X.; Fuster, F.; Silvi, B. Computational tools for the electron localization function topological analysis. *Comput. Chem.* **1999**, *23*, 597-604.

[29] (a) Capel, E.; Rodriguez-Rodriguez, M.; Uria, U.; Pedron, M.; Tejero, T.; Vicario, J. L.; Merino, P. Absence of Intermediates in the BINOL-derived Mg(II)/Phosphate-Catalyzed Desymmetrizative Ring Expansion of 1-Vinylcyclobutanols. *J. Org. Chem.* **2022**, *87*, 693-707. (b) Ortega, A.; Manzano, R.; Uria, U.; Carrillo, L.; Reyes, E.; Tejero, T.; Merino, P.; Vicario, J. L. Catalytic Enantioselective Cloke-Wilson Rearrangement. *Angew. Chem. Int. Ed.* **2018**, *57*, 8225-8229. (c) Merino, P.;

Chiacchio, M. A.; Legnani, L.; Delso, I.; Tejero, T. Introducing topology to assess the synchronicity of organic reactions. Dual reactivity of oximes with alkenes as a case study. *Org. Chem. Front.* **2017**, *4*, 1541-1554. (d) Bentabed-Ababsa, G.; Derdour, A.; Roisnel, T.; Saez, J. A.; Perez, P.; Chamorro, E.; Domingo, L. R.; Mongin, F. *J. Org. Chem.* **2009**, *74*, 2120-2133. (e) Polo, V.; Andres, J.; Castillo, R.; Berski, S.; Silvi, B. Understanding the Molecular Mechanism of the 1,3-Dipolar Cycloaddition between Fulminic Acid and Acetylene in Terms of the Electron Localization Function and Catastrophe Theory. *Chem. Eur. J.* **2004**, *10*, 5165-5172.

[30] (a) Johnson, E. R.; Keinan, S.; Mori-Sanchez, P.; Contreras-Garcia, J.; Cohen, A. J.; Yang, W. Revealing Noncovalent Interactions. *J. Am. Chem. Soc.* **2010**, *132*, 6498-6506. (b) Lane, J. R.; Contreras-Garcia, J.; Piquemal, J.-P.; Miller, B. J.; Kjaergaard, H. G. *J. Chem. Theory Comput.* **2013**, *9*, 3263-3266.

[31] Boto, R. A.; Peccati, F.; Laplaza, R.; Quan, C.; Carbone, A.; Piquemal, J.-P.; Maday, Y.; Contreras-García, J. NCIPLOT4: Fast, Robust, and Quantitative Analysis of Noncovalent Interactions. *J. Chem. Theory Comput.* **2020**, *16*, 4150-4158.

[32] Humphrey, W.; Dalke, A.; Schulten, K. VMD - Visual Molecular Dynamics. *J. Mol. Graph.* **1996**, *14*, 33-38.

[33] Williams, T.; Kelley, C. Gnuplot 4.5: an interactive plotting program. **2011**, http://gnuplot.info (visited Dec 1st, 2021).

[34] Case, D. A.; Belfon, K.; Ben-Shalom, I. Y.; Brozell, S. R.; Cerutti, D. S.; III, T. E. C.; Cruzeiro, V. W. D.; Darden, T. A.; Duke, R. E.; Giambasu, G.; Gilson, M. K.; Gohlke, H.; A.W. Goetz, R. H.; Izadi, S.; Izmailov, S. A.; Kasavajhala, K.; Kovalenko, A.; Krasny, R.; Kurtzman, T.; Lee, T. S.; LeGrand, S.; Li, P.; Lin, C.; Liu, J.; Luchko, T.; Luo, R.; Man, V.; Merz, K. M.; Miao, Y.; Mikhailovskii, O.; Monard, G.; Nguyen, H.; Onufriev, A.; Pan, F.; Pantano, S.; Qi, R.; Roe, D. R.; Roitberg, A.; Sagui, C.; Schott-Verdugo, S.; Shen, J.; Simmerling, C. L.; Skrynnikov, N. R.; Smith, J.; Swails, J.; Walker, R. C.; Wang, J.; Wilson, L.; Wolf, R. M.; Wu, X.; Xiong, Y.; Xue, Y.; York, D. M.; Kollman, P. A. *University of California, San Francisco* **AMBER 2020**.

[35] Wang, J.; Wolf, R. M.; Caldwell, J. W.; Kollman, P. A.; Case, D. A. Development and testing of a general AMBER force field. *J. Comput. Chem* **2004**, *25*, 1157-1174.

[36] Matubayasi, N.; Nakahara, M. Reversible molecular dynamics for rigid bodies and hybrid Monte Carlo. *J. Chem. Phys.* **1999**, *110*, 3291-3301.

[37] Darden, T.; York, D.; Pedersen, L. Particle mesh Ewald: An N·log(N) method for Ewald sums in large systems. *J. Chem. Phys.* **1993**, *98*, 10089-10092.

[38] Becke, A. D.; Edgecombe, K. E. A simple measure of electron localization in atomic and molecular systems. *J. Chem. Phys.* **1990**, *92*, 5397-5403.

12. NMR spectra of new compounds

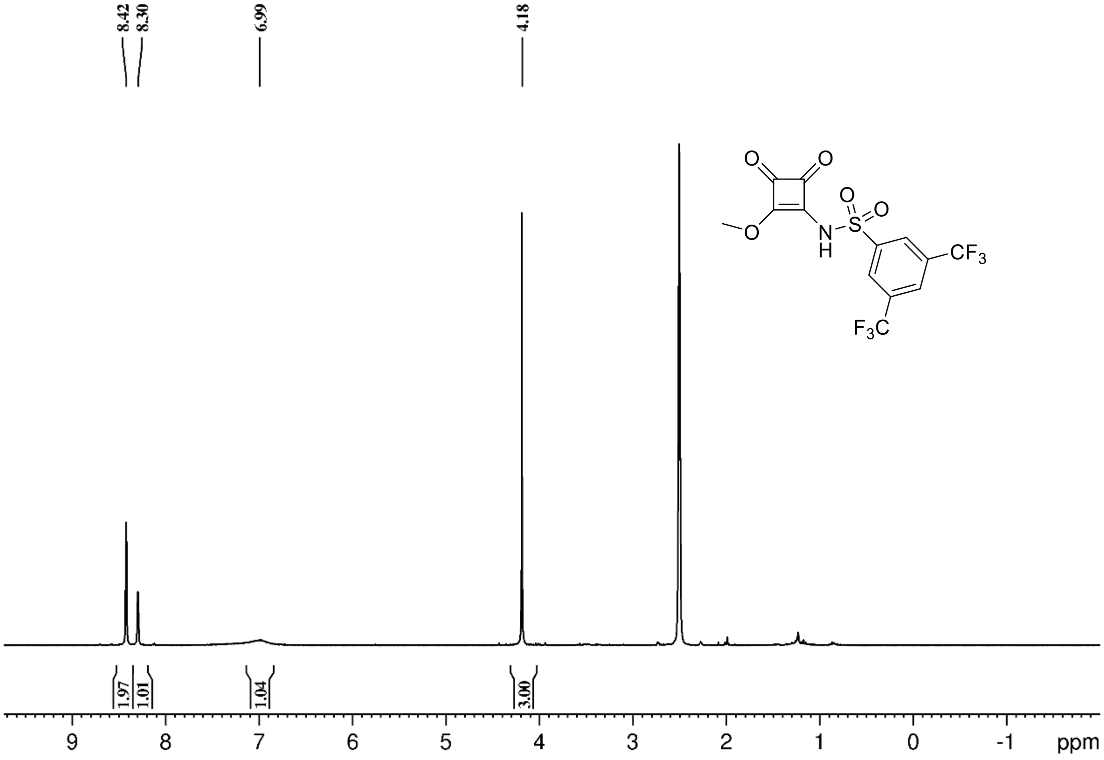

^{1}H NMR (300 MHz, DMSO-d_6) of **P2**

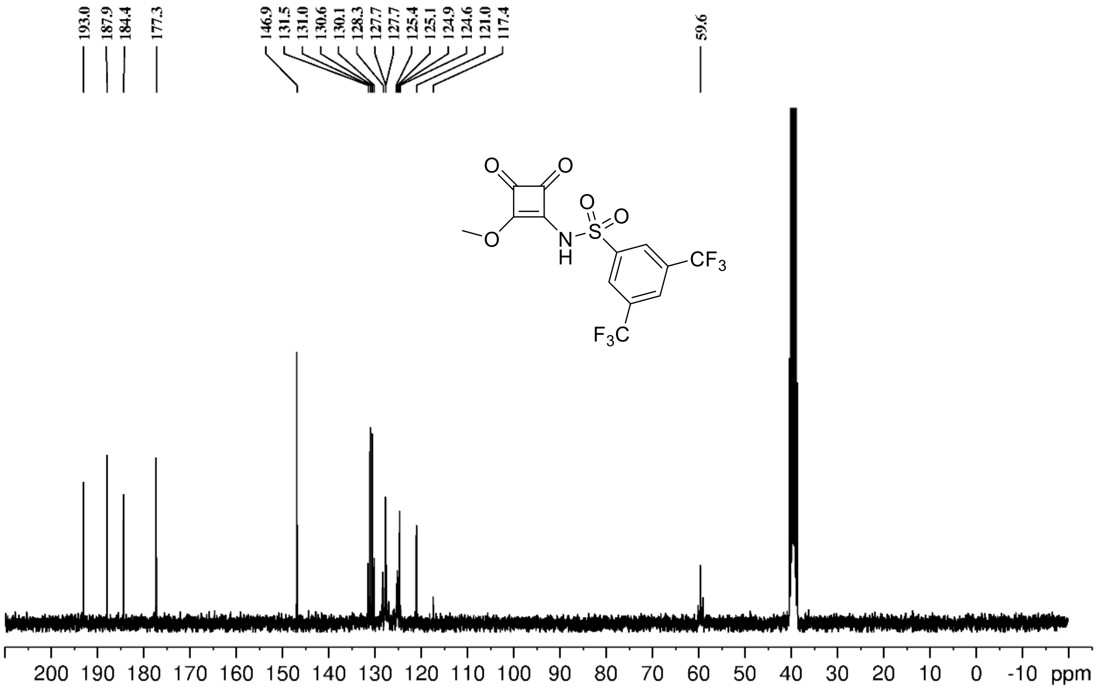

^{13}C{1H} NMR (75.5 MHz, DMSO-d_6) of **P2**

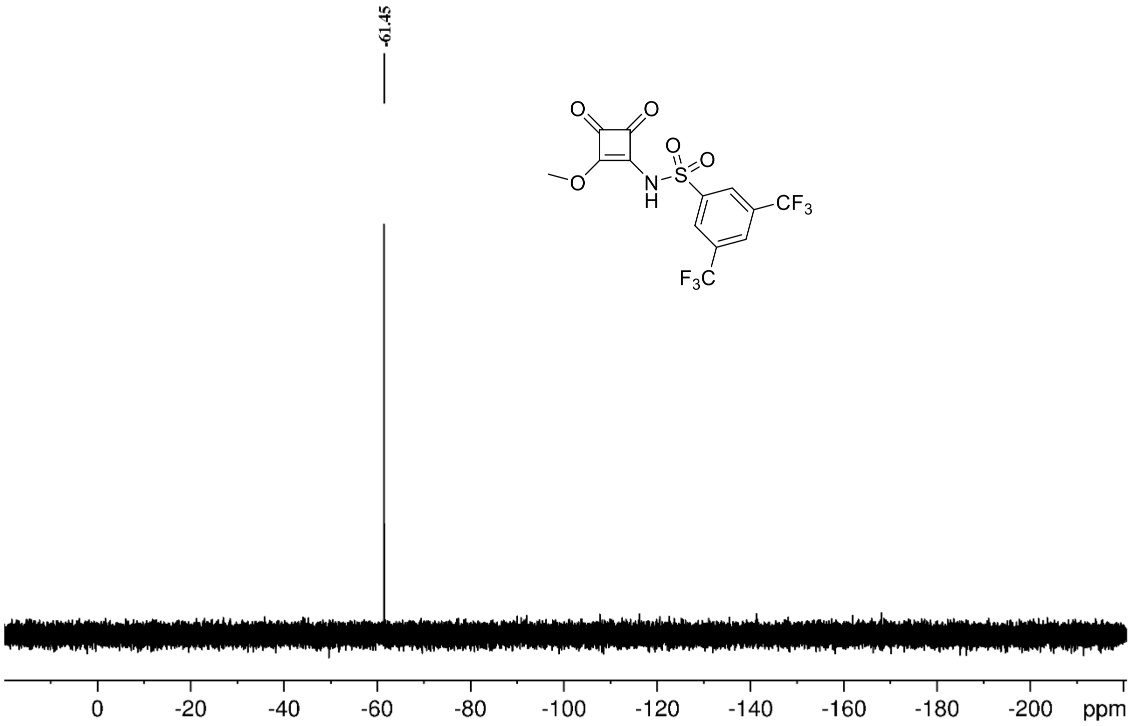

^{19}F NMR (471 MHz, DMSO-d_6) of **P2**

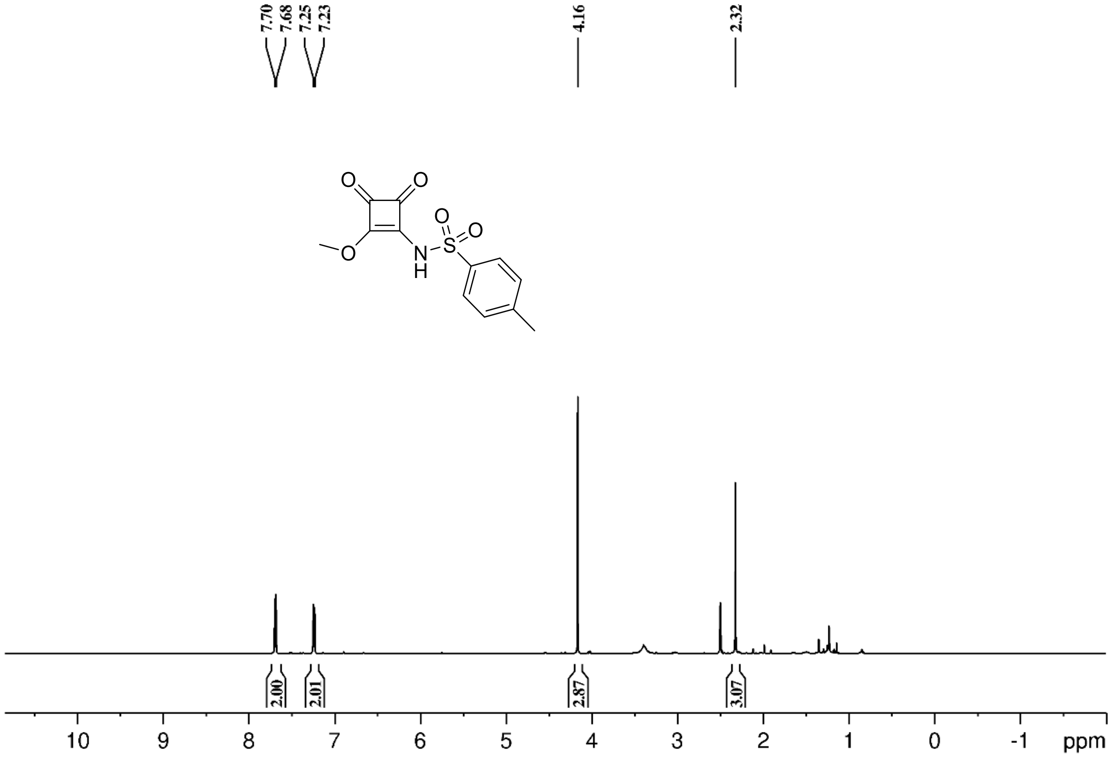

^{1}H NMR (500 MHz, DMSO-d_6) of **P3**

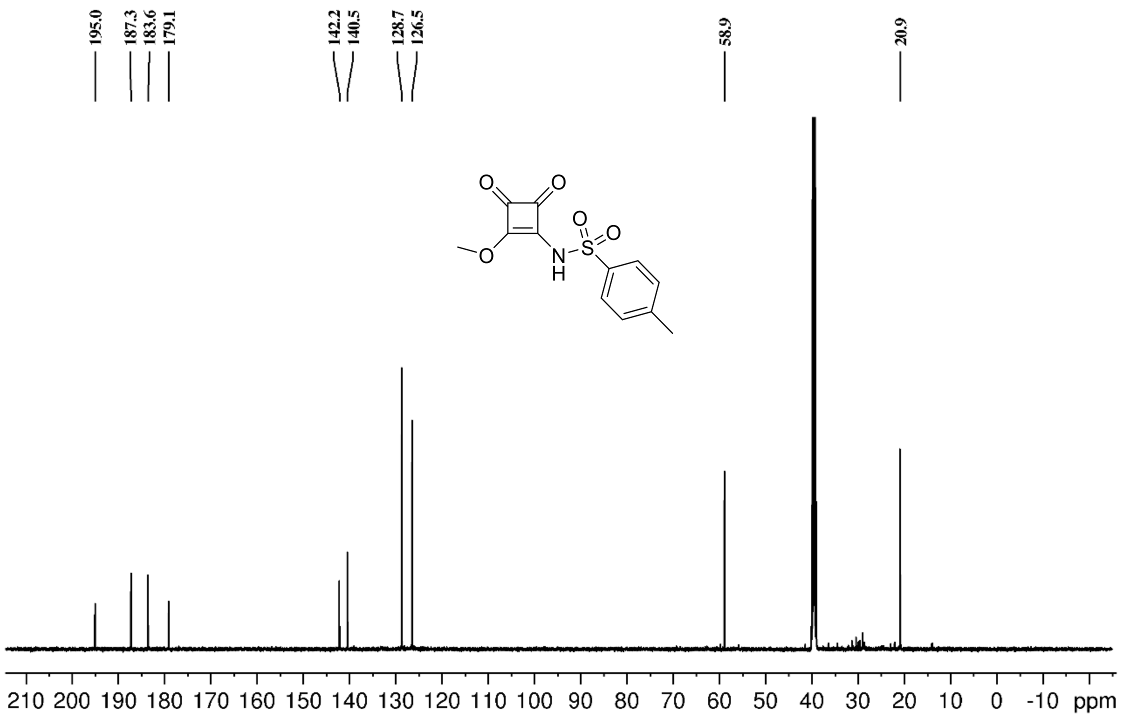

^{13}C{1H} NMR (126 MHz, DMSO-d_6) of **P3**

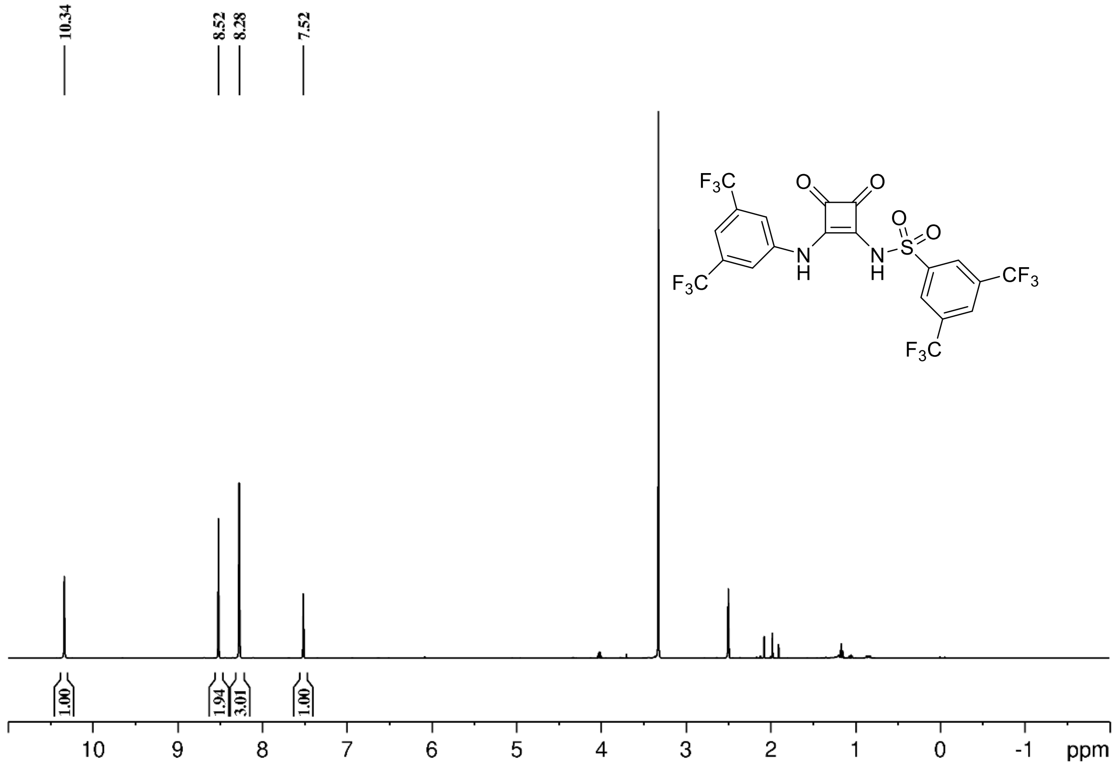

^{1}H NMR (500 MHz, DMSO-d_6) of **I**

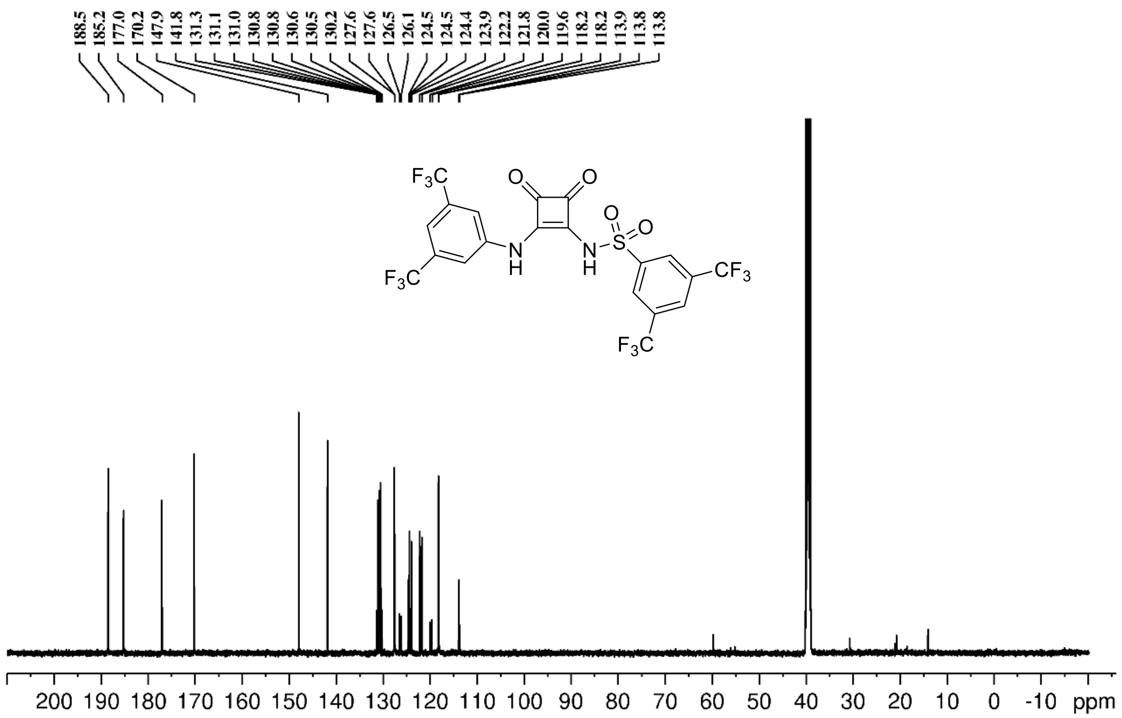

^{13}C{1H} NMR (126 MHz, DMSO-d_6) of **I**

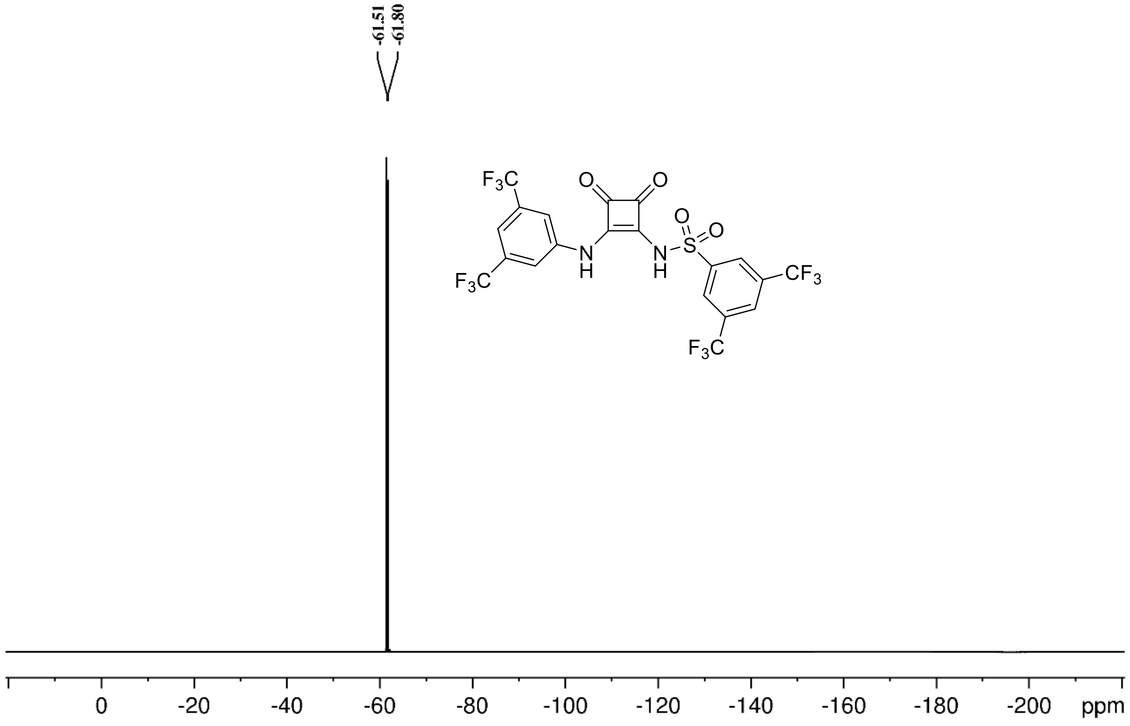

^{19}F NMR (471 MHz, DMSO-d_6) of **I**

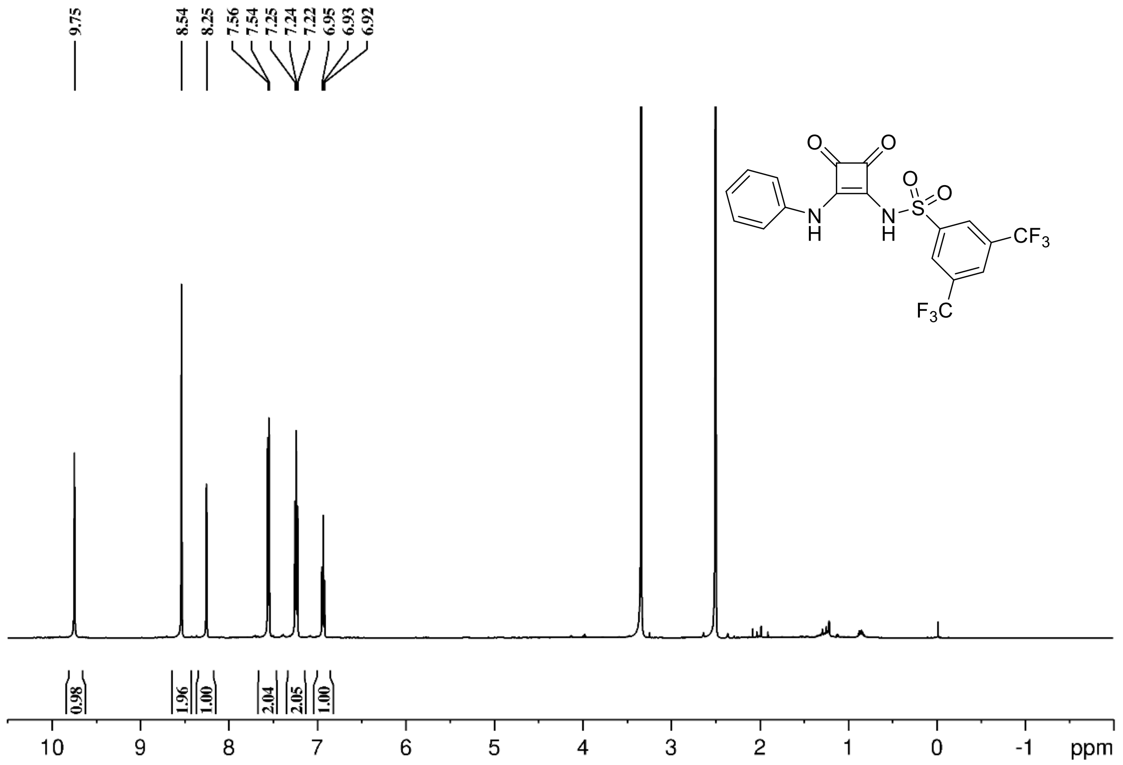

^{1}H NMR (500 MHz, DMSO-d_6) of **II**

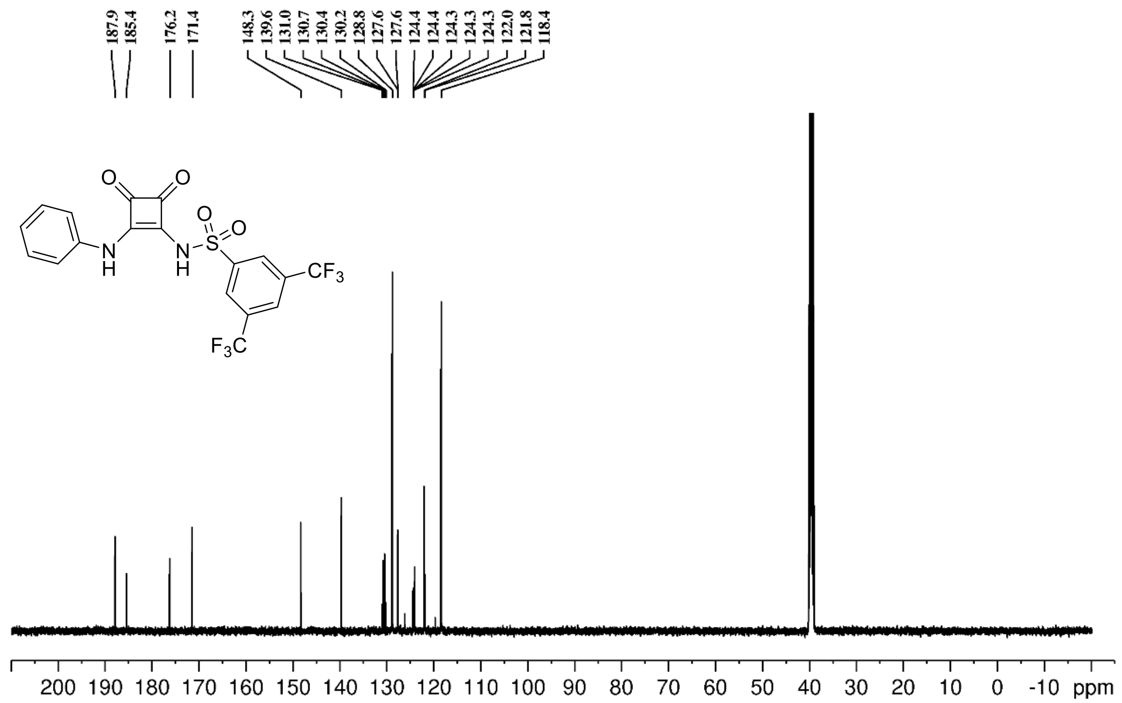

^{13}C{1H} NMR (75.5 MHz, DMSO-d_6) of **I**

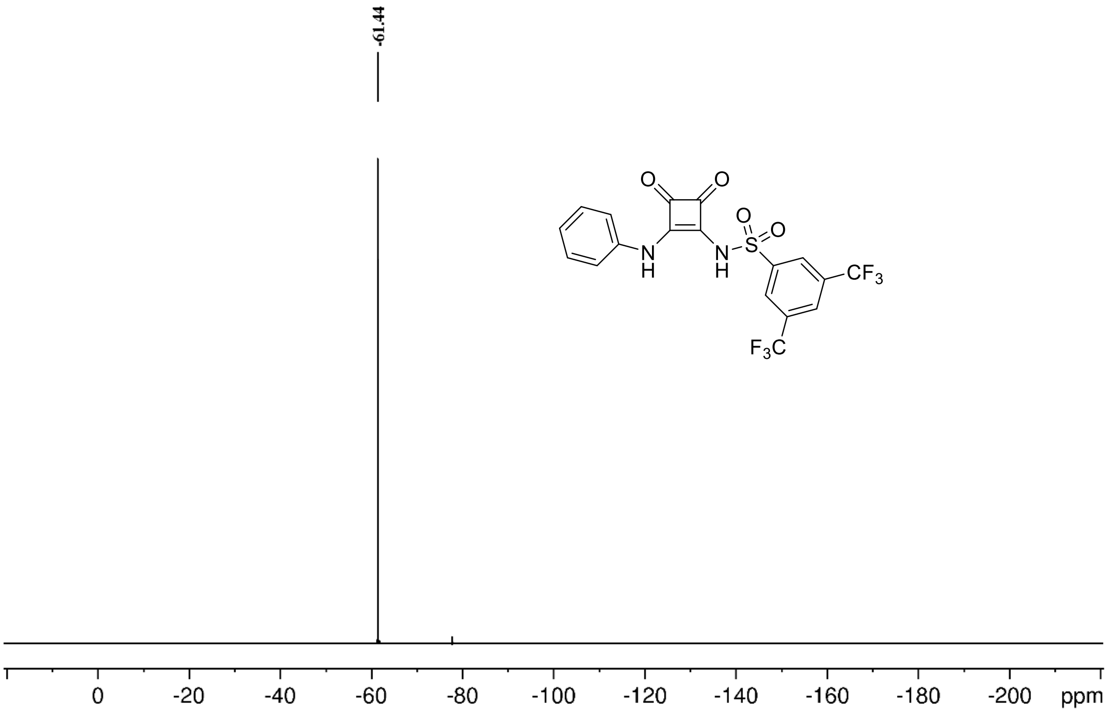

^{19}F NMR (471 MHz, DMSO-d_6) of **II**

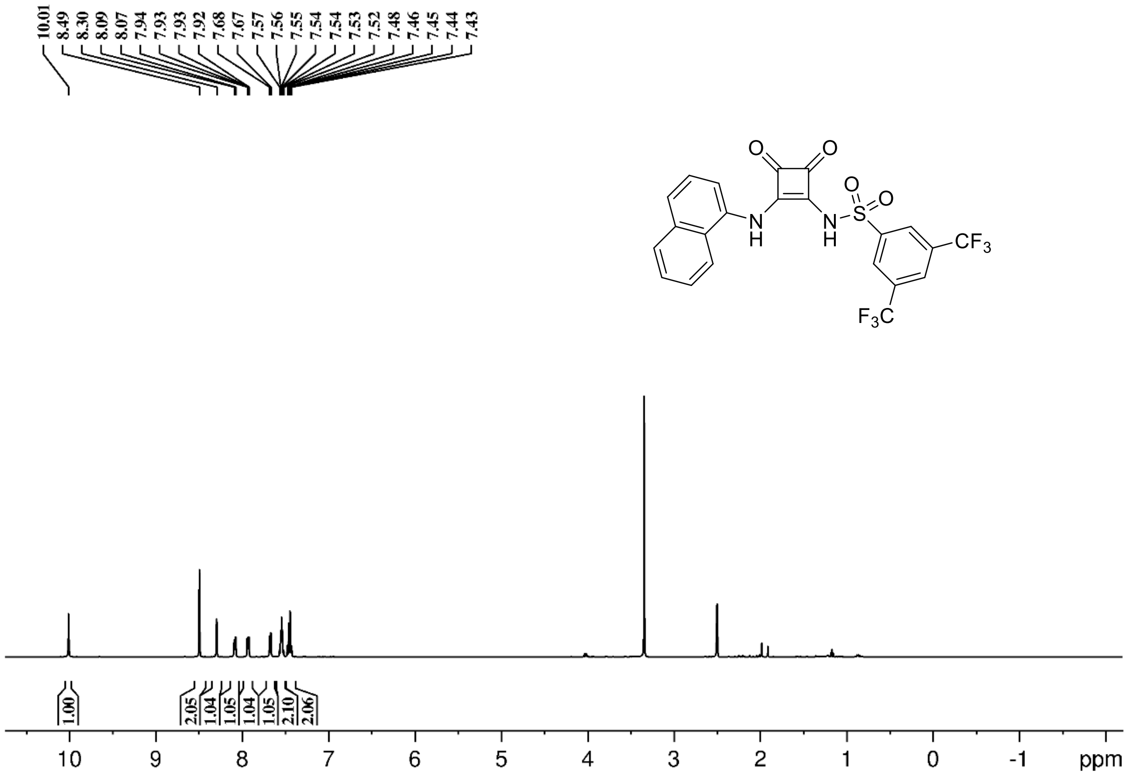

^{1}H NMR (500 MHz, DMSO-d_6) of **III**

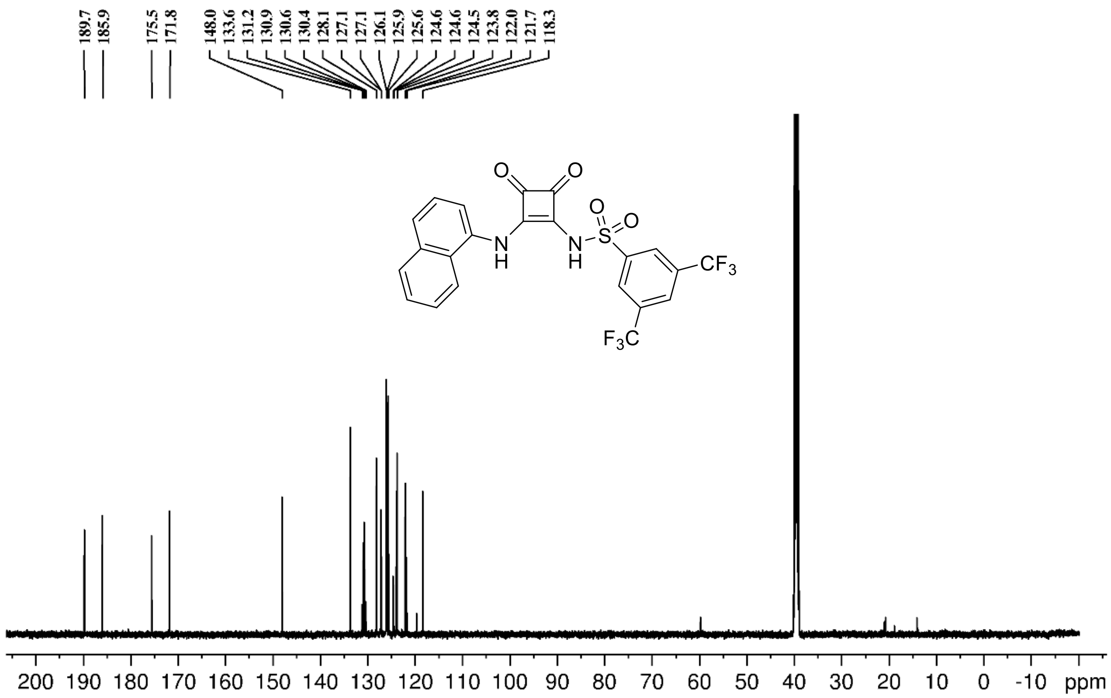

^{13}C{1H} NMR (126 MHz, DMSO-d_6) of **III**

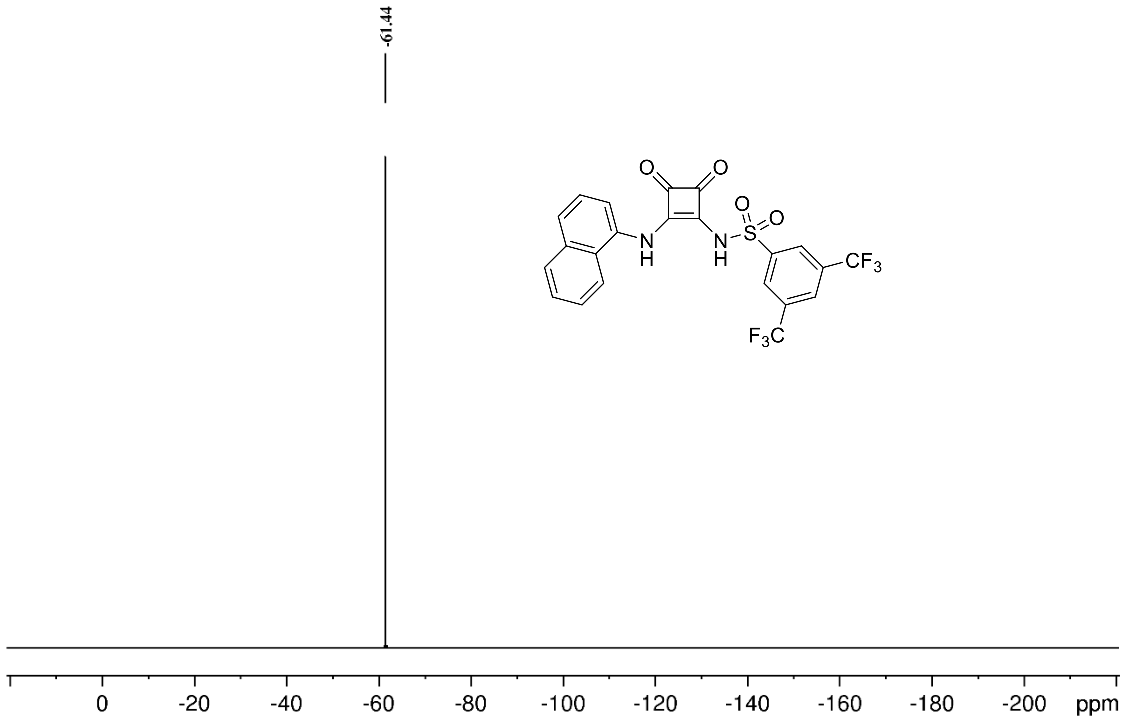

^{19}F NMR (471 MHz, DMSO-d_6) of **III**

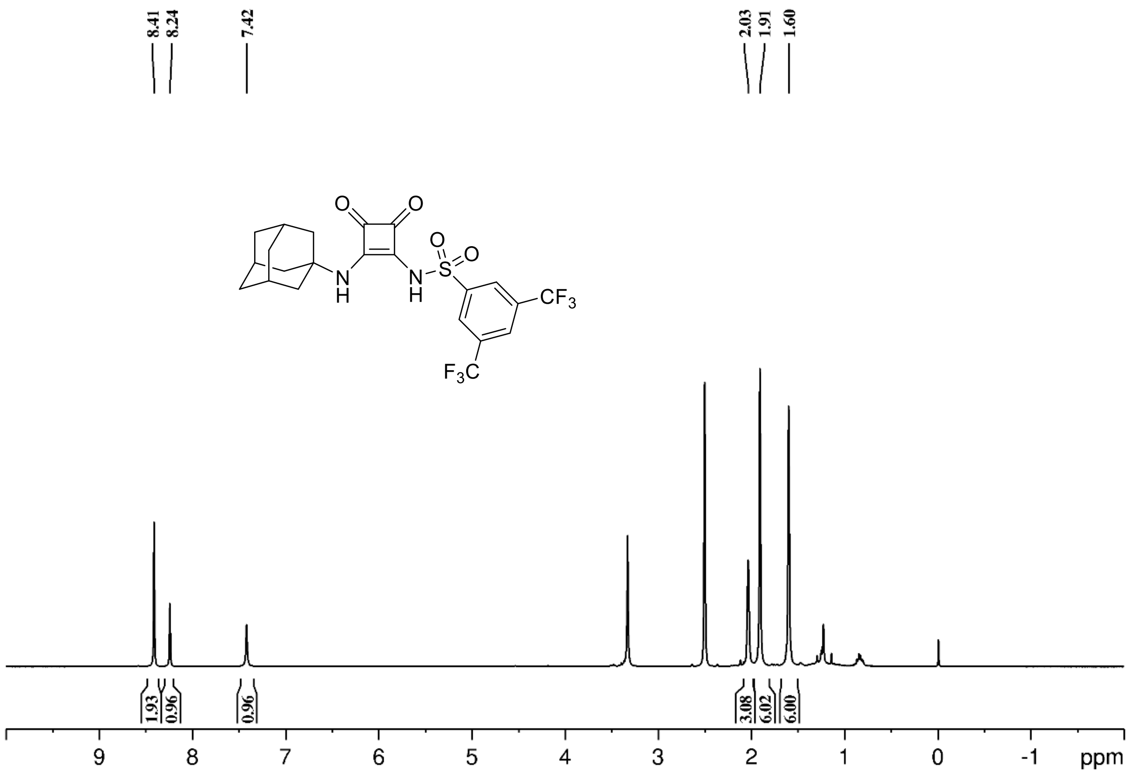

^{1}H NMR (500 MHz, DMSO-d_6) of **IV**

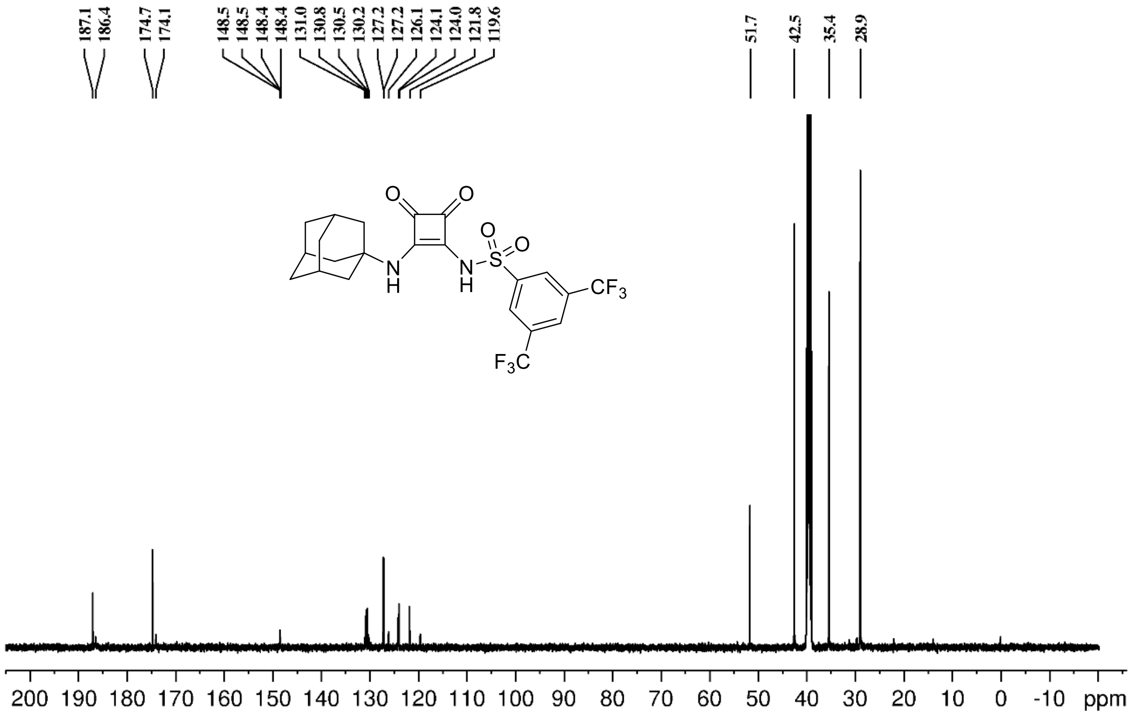

^{13}C{1H} NMR (126 MHz, DMSO-d_6) of **IV**

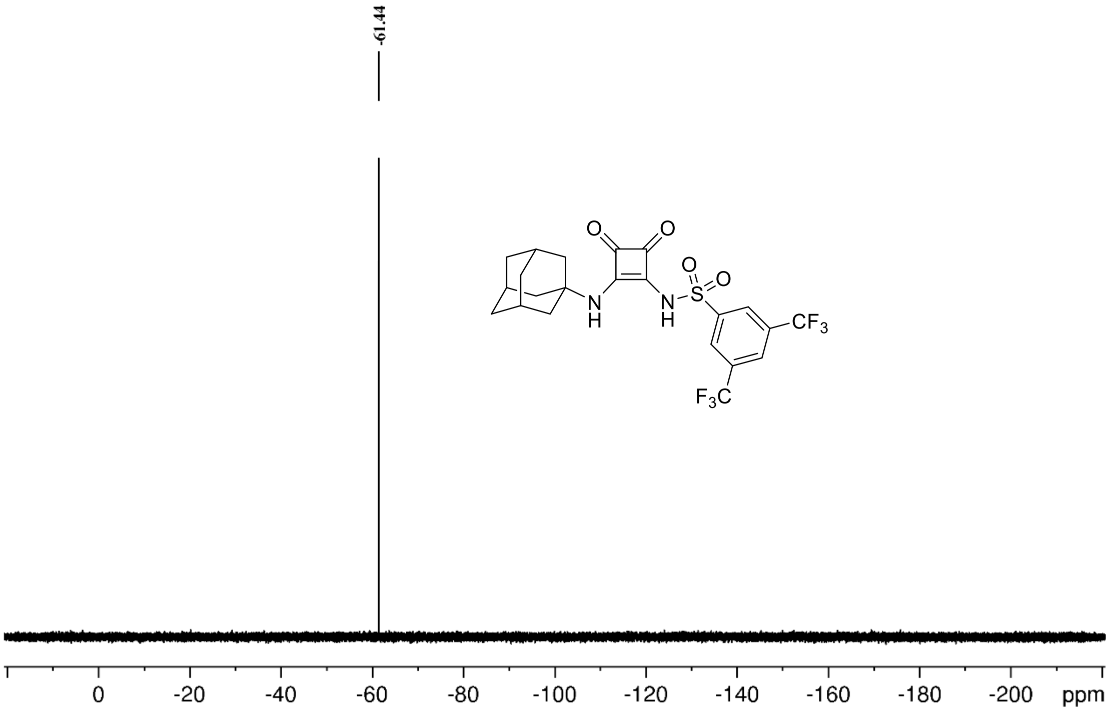

^{19}F NMR (471 MHz, DMSO-d_6) of **IV**

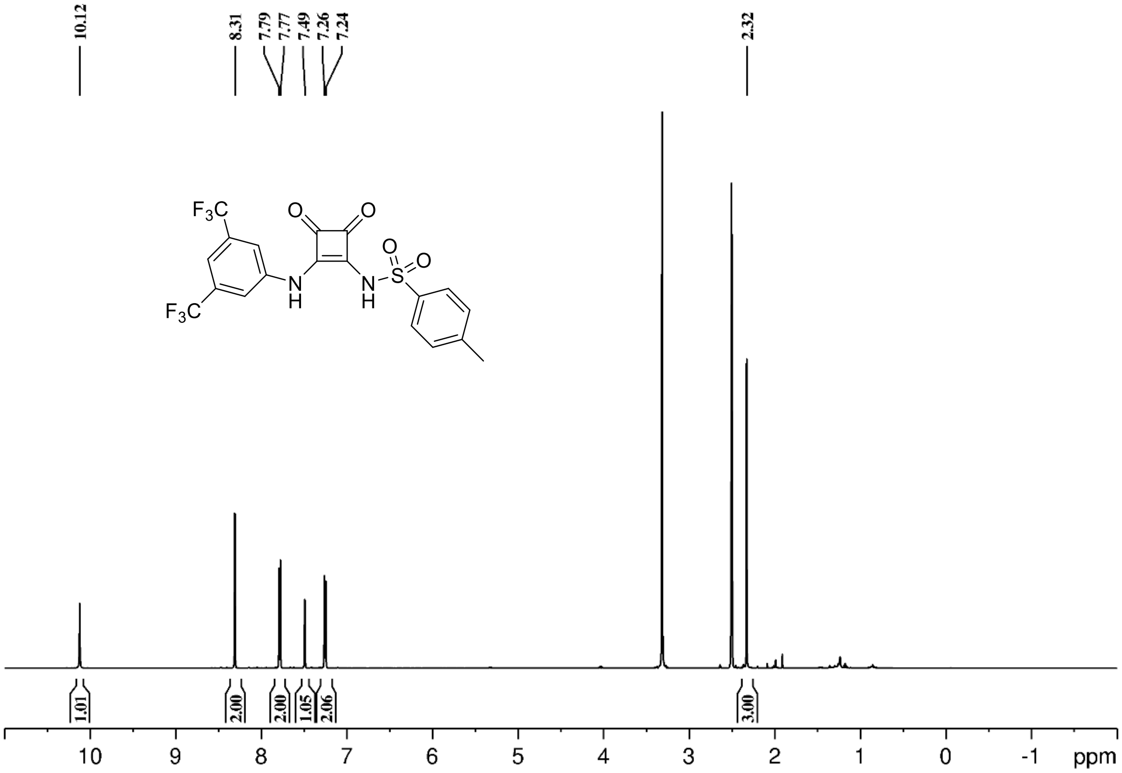

^{1}H NMR (500 MHz, DMSO-d_6) of **V**

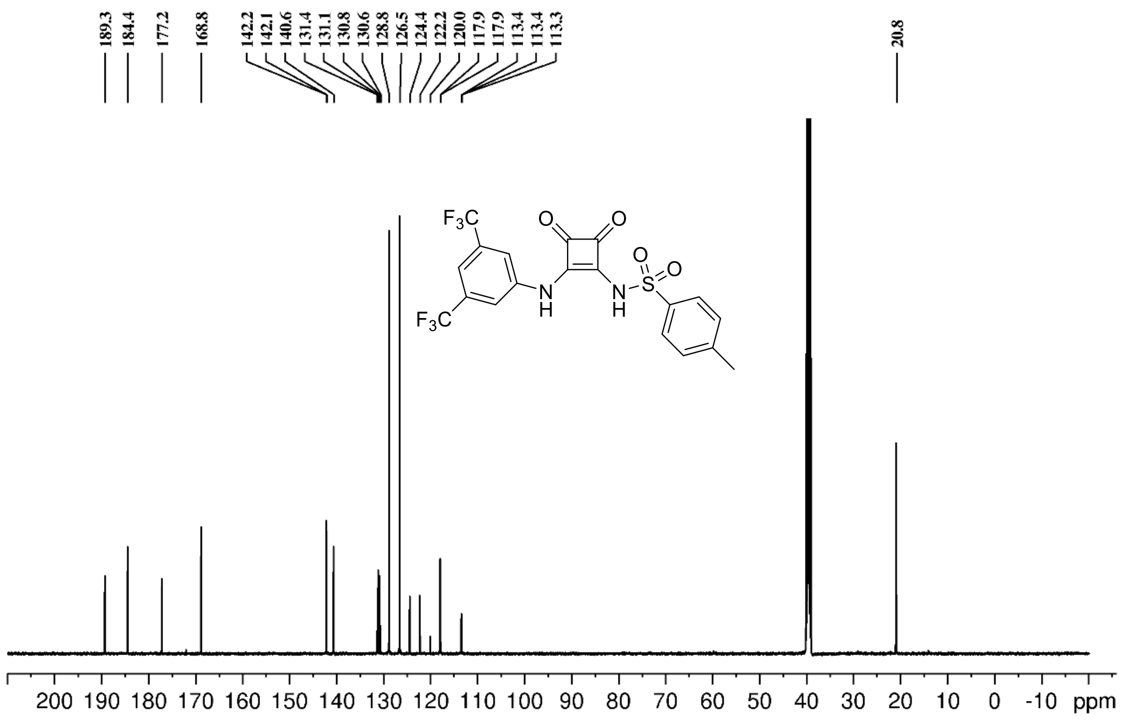

^{13}C{1H} NMR (126 MHz, DMSO-d_6) of **V**

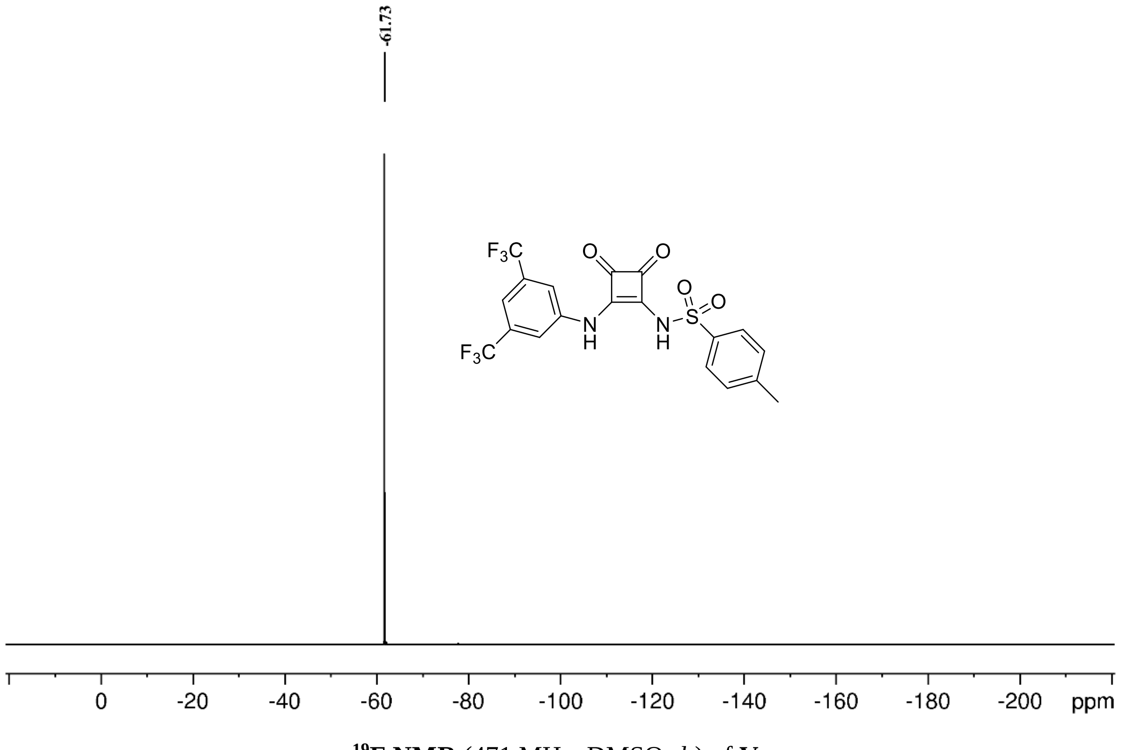

^{19}F NMR (471 MHz, DMSO-d_6) of **V**

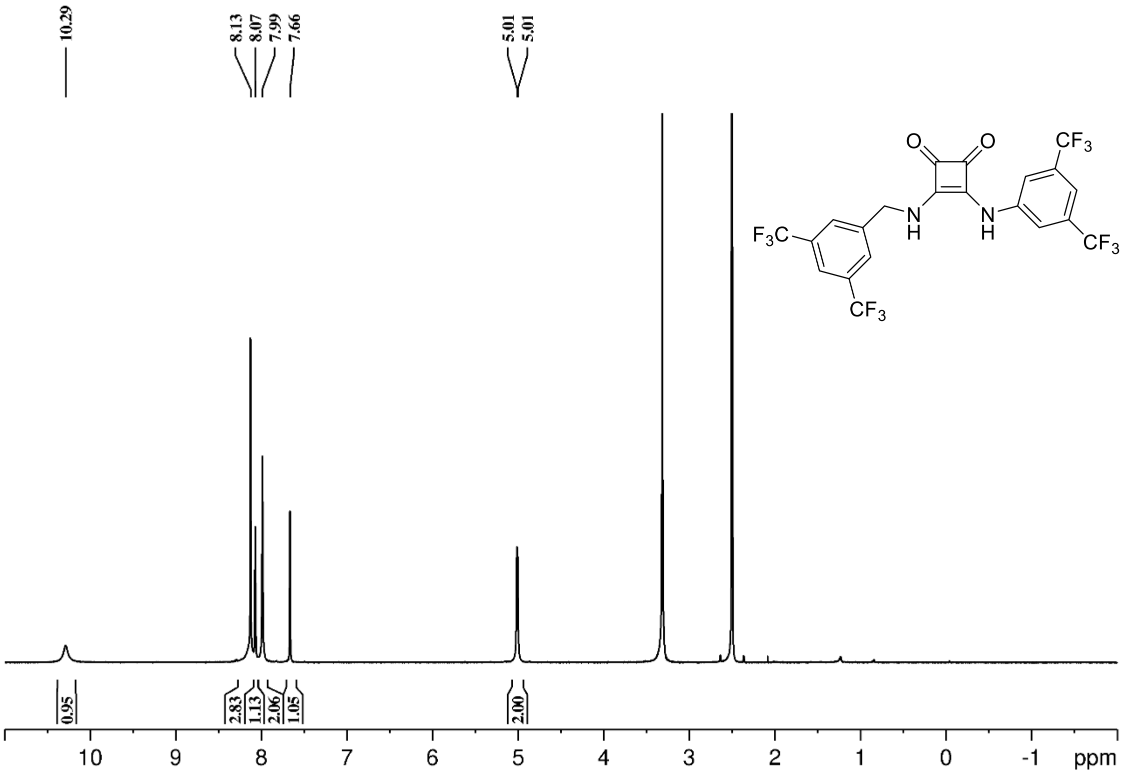

^{1}H NMR (500 MHz, DMSO-d_6) of **VII**

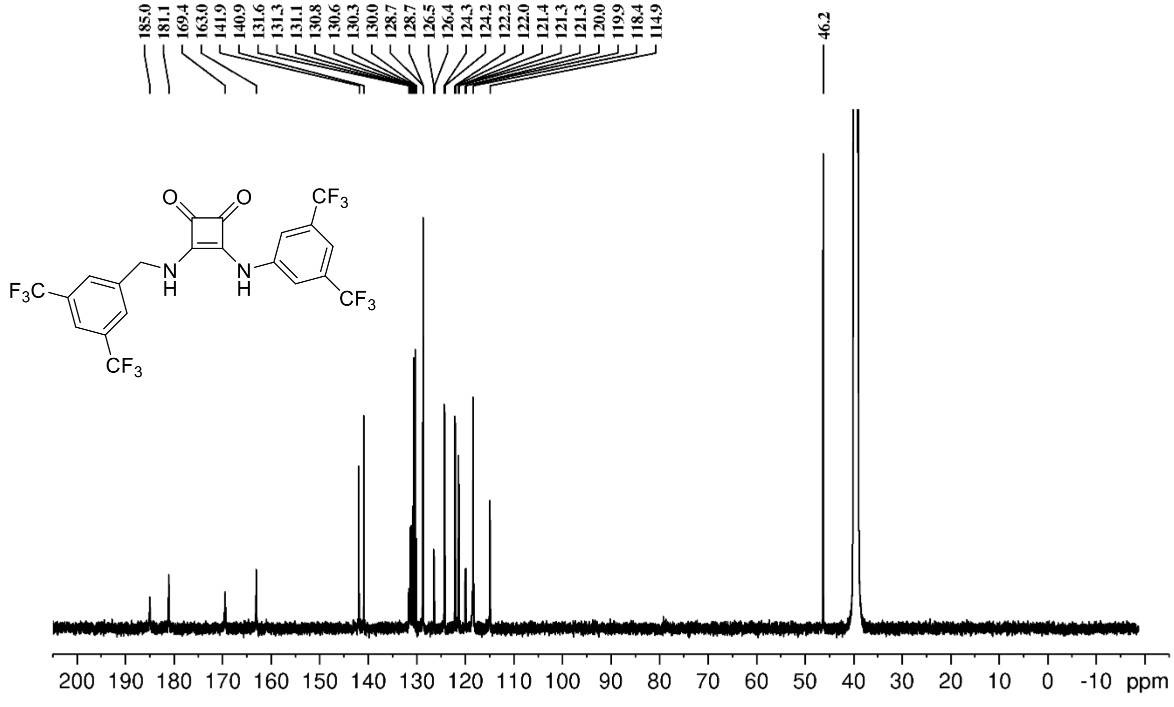

^{13}C{1H} NMR (126 MHz, DMSO-d_6) of **VII**

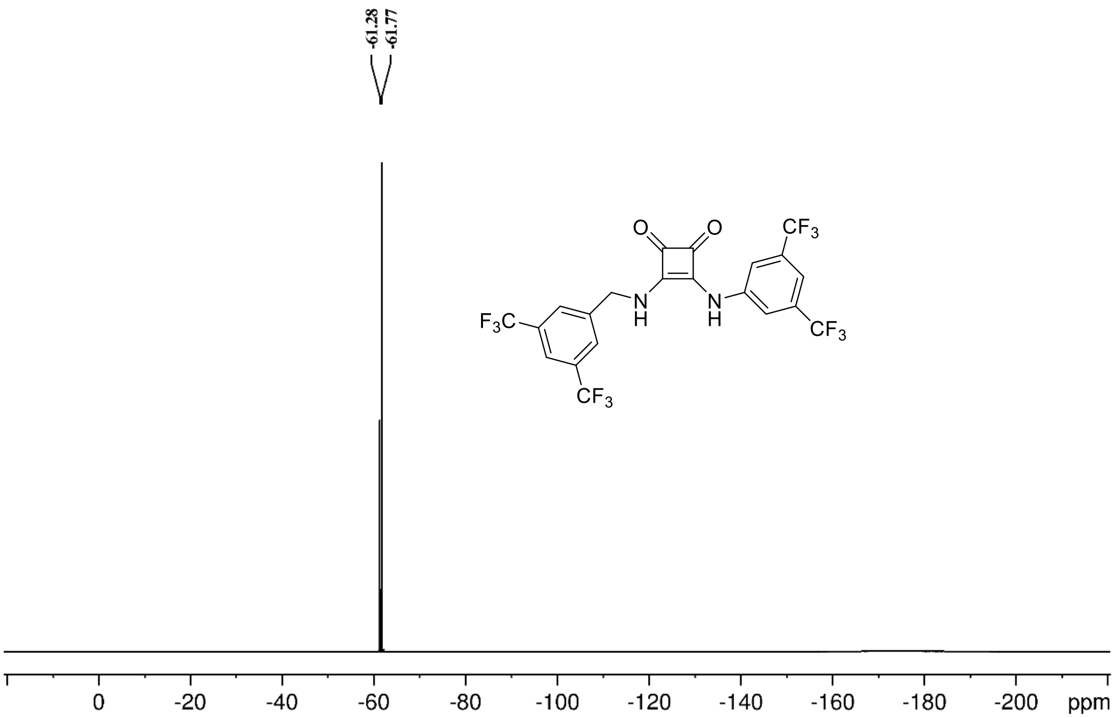

^{19}F NMR (471 MHz, DMSO-d_6) of **VII**
