# Supplementary figures and images for: Silver-Free Gold-Catalyzed Heterocyclizations through Intermolecular H-Bonding Activation
Source: J Org Chem. 2023 Jan 27;88(4):2487–92. doi: 10.1021/acs.joc.2c02932 (PMC9942198; doi:10.1021/acs.joc.2c02932)

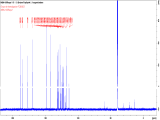

Supplement: Supplementary file 2 — jo2c02932_si_002.zip [file jo2c02932_si_002.zip › FID for publication/Compound I/13C 126 MHz/15/pdata/1/thumb.png]

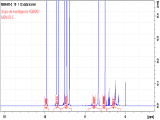

Supplement: Supplementary file 2 — jo2c02932_si_002.zip [file jo2c02932_si_002.zip › FID for publication/Compound II/1H 500 MHz/10/pdata/1/thumb.png]
