# Supplementary material for: Silver-Free Gold-Catalyzed Heterocyclizations through Intermolecular H-Bonding Activation
Source: J Org Chem. 2023 Jan 27;88(4):2487–92. doi: 10.1021/acs.joc.2c02932 (PMC9942198; doi:10.1021/acs.joc.2c02932)
Supplement: Supplementary file 2 — jo2c02932_si_002.zip [file jo2c02932_si_002.zip › FID for publication/NMR.pdf]

- **Manufacturer of the spectrometer used:** Bruker

- **Acquisition software:** Topspin 4.1.4

- **Processing program:** Topspin 4.1.1

- **Field strength:**
  **$^{1}$H:** 300 MHz (compound P2) and 500 MHz (compounds P3, I, II, III, IV, V, VII).
  **$^{13}$C:** 75.5 MHz (compound P2) and 126 MHz (compounds P3, I, II, III, IV, V, VII).
  **$^{19}$F:** 471 MHz (all compounds).
